# Two different epigenetic information channels in wild three-spined sticklebacks are involved in salinity adaptation

**DOI:** 10.1126/sciadv.aaz1138

**Published:** 2020-03-20

**Authors:** Melanie J. Heckwolf, Britta S. Meyer, Robert Häsler, Marc P. Höppner, Christophe Eizaguirre, Thorsten B. H. Reusch

**Affiliations:** 1 Marine Evolutionary Ecology, GEOMAR Helmholtz Centre for Ocean Research Kiel, Kiel, Germany.; 2 Institute of Clinical Molecular Biology, Kiel University, Kiel, Germany.; 3 School of Biological and Chemical Sciences, Queen Mary University of London, London, UK.

## Abstract

Epigenetic inheritance has been proposed to contribute to adaptation and acclimation via two information channels: (i) inducible epigenetic marks that enable transgenerational plasticity and (ii) noninducible epigenetic marks resulting from random epimutations shaped by selection. We studied both postulated channels by sequencing methylomes and genomes of Baltic three-spined sticklebacks (*Gasterosteus aculeatus*) along a salinity cline. Wild populations differing in salinity tolerance revealed differential methylation (pop-DMS) at genes enriched for osmoregulatory processes. A two-generation experiment demonstrated that 62% of these pop-DMS were noninducible by salinity manipulation, suggesting that they are the result of either direct selection or associated genomic divergence at cis- or trans-regulatory sites. Two-thirds of the remaining inducible pop-DMS increased in similarity to patterns detected in wild populations from corresponding salinities. The level of similarity accentuated over consecutive generations, indicating a mechanism of transgenerational plasticity. While we can attribute natural DNA methylation patterns to the two information channels, their interplay with genomic variation in salinity adaptation is still unresolved.

## Teaser

Detection of both inducible and stable DNA methylation indicates two different epigenetic modes of salinity adaptation.

## INTRODUCTION

Recent advances in epigenetics challenge our understanding of inheritance and adaptive evolution (*1*–*3*). It has been suggested that epigenetic modifications—for example, via DNA methylation, histone modification, or small RNAs—create phenotypic diversity and ultimately contribute to rapid evolutionary adaptation (*4*–*6*). Several theoretical models posit that the heritable proportion of these molecular modifications can be classified into two distinct information channels (*5*, *7*, *8*). Selection-based epigenetic marks emerge as spontaneous epimutations that remain stable across subsequent generations, although their overall stability is three to four orders of magnitude lower compared to DNA base changes (*7*, *9*). Similar to adaptation from DNA sequence–based variation, these epimutations may result in different phenotypes that become targets of natural selection and thereby carry information on past selection regimes without directly responding to the current environment (*5*, *8*, *10*). On the other hand, detection-based effects describe inducible epigenetic marks at defined genomic locations, which are under environmental control (*7*). Such transfer of parental information linked to environmental cues represents a rapid and reliable mechanism underlying transgenerational plasticity, which is hypothesized to buffer the extinction risk of populations under sudden environmental change until genetic adaptations can catch up (“genetic rescue”) (*7*, *11*). Distinguishing between these mechanisms is important because they have very different implications for the evolution of populations. Stable epigenetic marks follow evolutionary principles of DNA sequence–based inheritance with random variation shaped by selection. In contrast, directional processes via inducible epigenetic marks can be considered a transgenerational form of plasticity that involve previously evolved regulatory mechanisms targeting specific sites on the genome. While the principal differences between these two transmission channels are clear (*4*, *5*, *7*), empirical evidence for their presence in wild vertebrate populations is lacking.

Here, we assess whether these two epigenetic information channels can be detected in nature and test whether short-term acclimation responses match patterns of DNA methylation variation of locally adapted populations. Transgenerational experiments that yield DNA methylation profiles more similar to those of locally adapted natural populations would provide evidence that DNA methylation is mechanistically involved in adaptive transgenerational plasticity.

Studying adaptation to ocean salinity is particularly suited to identification of selection- and detection-based effects because spatiotemporal patterns in ocean salinity are more stable than other variables, for instance, temperature. Since salinity change imposes strong physiological stress with well-defined cellular effects (*12*), natural salinity gradients offer unparalleled opportunities to use local patterns of epigenetic variation as background against which direction and magnitude of results from experimental salinity manipulations can be tested. One suitable ecosystem to follow such a space-for-time approach is the Baltic Sea, which is a semi-enclosed marginal sea that has been dubbed a “time machine” to evaluate the predicted perturbations associated with global change (*13*).

Taking advantage of the Baltic Sea salinity gradient, we sequenced the methylomes [reduced representation bisulfite sequencing (RRBS)] and whole genomes of three-spined sticklebacks (*Gasterosteus aculeatus*) from three populations that are locally adapted to different salinities [6, 20, and 33 practical salinity units (PSU)] (*14*) in and outside the Baltic Sea. Specifically, we focus on the patterns of (epi)genomic variation, while transgenerational phenotypic effects have been described previously for the exact same populations (*15*).

**Fig. 1:**
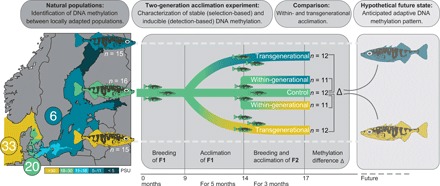
Experimental space-for-time approach. We characterized DNA methylation profiles (via RRBS) and whole genomes [whole-genome sequencing (WGS)] of fish from three populations of wild-caught three-spined sticklebacks locally adapted to 6 (blue; *n* = 15), 20 (green; *n* = 16), and 33 (yellow; *n* = 15) PSU. We also bred and acclimated sticklebacks from the mid-salinity location (20 PSU) within one (“within-generational”) or over two (“transgenerational”) generations to decreased (6 PSU) or increased (33 PSU) salinity while maintaining a control group at its original salinity (*n* = 11 to 12 per group; see details in the figure). Differential methylation within and across generations was assessed and compared to natural populations locally adapted to the corresponding salinity, serving as the hypothetical future DNA methylation state to capture long-term adaptation processes.

Baltic stickleback populations are genetically differentiated [genome-wide average pairwise *F*_ST_ = 0.028 (*14*)] and show patterns consistent with local adaptation to salinity regimes in controlled common garden experiments (*15*, *16*). Moreover, previous studies have revealed transgenerational plasticity in response to variation in temperature (*17*) and changes in DNA methylation levels at osmoregulatory genes in response to within-generational salinity manipulation (*18*, *19*). However, it remains unclear whether DNA methylation mediates transgenerational plasticity, a possible mechanism enabling adaptive phenotypes to rapidly emerge in the face of environmental change. In this study, we consider transgenerational effects to be adaptive if the preacclimation of the parents enhances the fitness of the offspring, sometimes referred to as intergenerational effects. To address this question, we complemented our field survey with a two-generation salinity acclimation experiment using the mid-salinity population (20 PSU). This experiment enabled us to quantify the proportion of noninducible (stable, potentially selection-based) and inducible (potentially detection-based) DNA methylation within and across generations (Fig. 1), acknowledging that we tested methylation mark stability only with respect to experimental salinity manipulation. We focused on the methylation of cytosines at cytosine-phosphate-guanine dinucleotides (CpG sites), the most common methylation motif in vertebrates (*20*), with partial inheritance potentially involved in adaptive evolution (*11*).

**Fig. 2:**
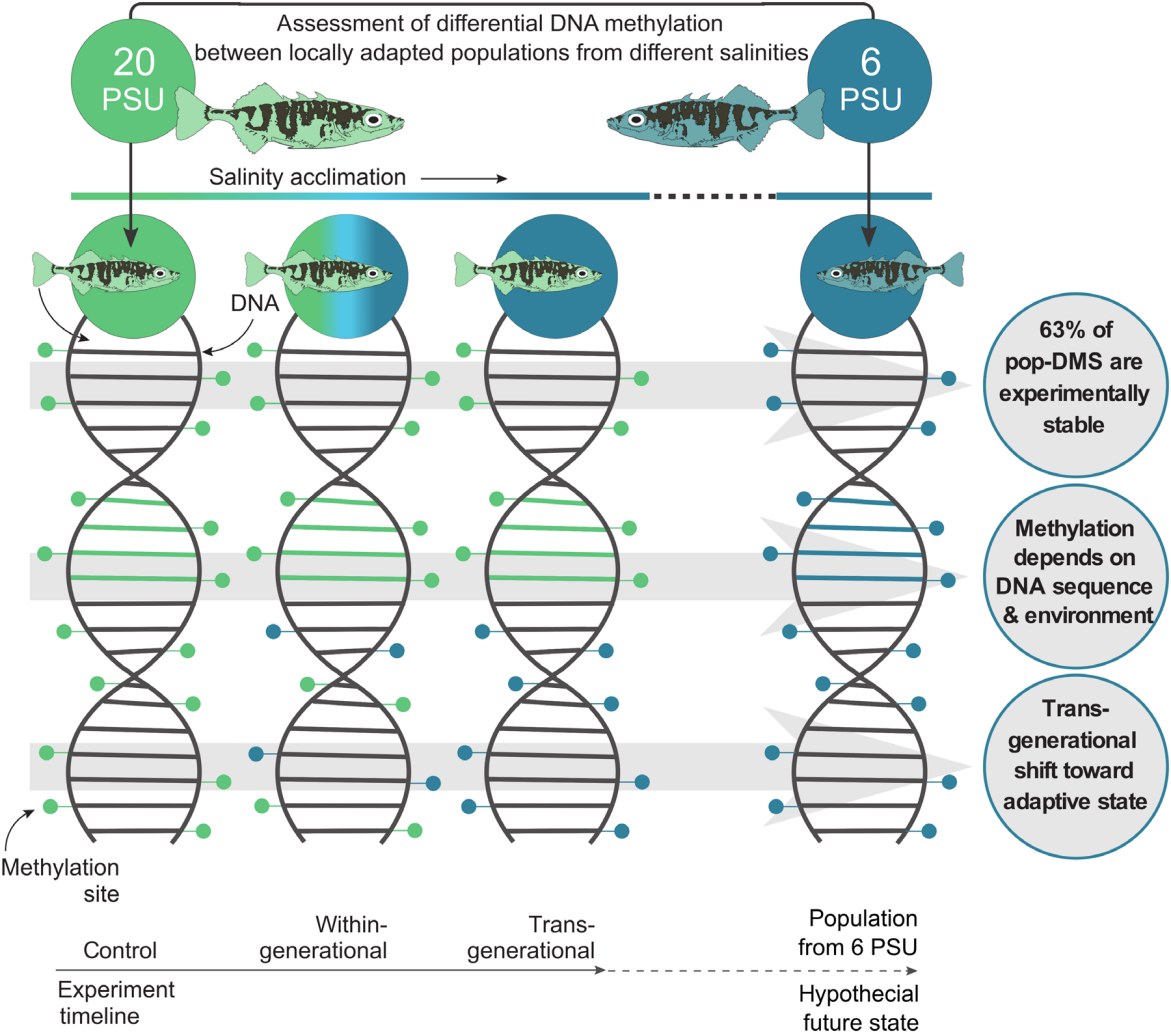
Graphical summary of the main results. We used the Baltic Sea salinity gradient to study the role of DNA methylation in local salinity adaptation and the response to salinity change in a space-for-time approach. To assess the potential future acclimatization and adaptation processes of the natural stickleback population from 20 PSU (KIE; green) to the predicted desalination (*63*), we compared differences in DNA methylation at CpG sites between wild-caught and laboratory-bred sticklebacks. Following the experiment timeline (bottom), we compared methylation levels of the experimental control group from 20 PSU to within- and transgenerational acclimation of 20 PSU sticklebacks to 6 PSU (DNA from left to right). The population locally adapted to 6 PSU serves as the hypothetical future state in which salinities will decrease (blue; DNA on the right). The three main results are written in the circles with schematically and horizontally corresponding DNA methylation changes. (i) Sixty-three percent of the DMS between the populations remained stable under experimental salinity change. (ii) The direction of experimental methylation change was dependent not only on the treatment but also on the degree of genetic differentiation between the populations [see Fig. 4 (A to D) for results]. (iii) Transgenerational salinity acclimation shifted DNA methylation patterns closer to the anticipated adaptive state found in the hypothetical future population [see Fig. 4 (E to H) for results]. For clarity, only one (6 PSU) of the two foreign salinity regimes tested (6 and 33 PSU) is shown. The results for the experimental fish acclimated to 33 PSU were very similar (see Fig. 1 for full experimental design and Fig. 4 for results).

We tested three nonexclusive hypotheses: (i) Stickleback populations from different salinities (6, 20, and 33 PSU) show differentially methylated CpG sites (hereafter referred to as pop-DMS). (ii) Such pop-DMS include both types of methylation sites: experimentally stable sites (potentially selection based) and experimentally inducible sites (potentially detection based). (iii) Upon transgenerational salinity acclimation, inducible DNA methylations become more similar to the patterns of natural populations at corresponding salinities. When associated with beneficial phenotypic effects and increased relative fitness, the latter would be evidence for a mechanism underlying adaptive transgenerational plasticity (overview in Fig. 2).

## RESULTS

### Identifying differentially methylated CpG sites between stickleback populations along a natural salinity cline

**Fig. 3:**
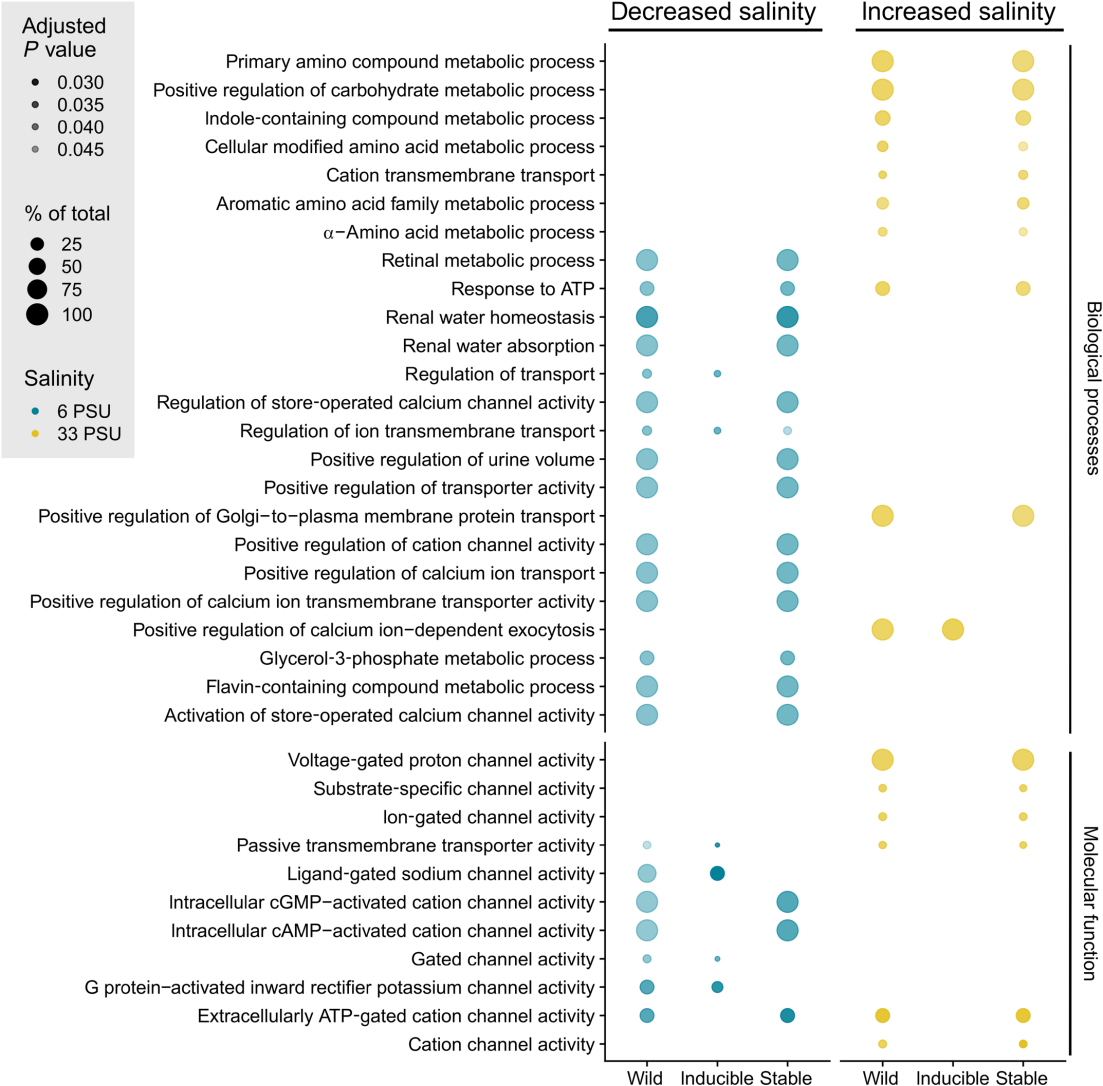
Gene Ontology terms for biological processes and molecular functions. Gene Ontology (GO) terms for biological processes and molecular functions under salinity increase (20 versus 33 PSU; yellow) and decrease (20 versus 6 PSU; blue) associated with pop-DMS are presented. The graph is split into GO terms associated with pop-DMS from natural stickleback populations across a salinity cline (wild) and their experimental inducibility (inducible and stable) in a two-generation acclimation experiment. The size of the circles refers to the number of genes of this term in the groups (in %), and the transparency refers to the false discovery rate–corrected *P* value (darker circles refer to a lower adjusted *P* value). This subset is filtered for GO terms including the following keywords: “channel,” “transport,” “water,” “chloride,” “potassium,” “homeostasis,” “ion-dependent,” “urine,” “ATP” (adenosine 5′-triphosphate), and “metabolic”; see fig. S2 for the full figure. cGMP, guanosine 3′,5′-monophosphate; cAMP, adenosine 3′,5′-monophosphate; G protein, heterotrimeric GTP-binding protein.

pop-DMS were determined via RRBS in 46 wild-caught sticklebacks from three different sites that varied in average salinity [Sylt (SYL), 33 PSU; Kiel (KIE), 20 PSU; Nynäshamn (NYN), 6 PSU; Fig. 1]. After quality and coverage filtering, we obtained 525,985 CpG sites present in all groups (*q* < 0.0125; methylation difference, ≥15%), corresponding to ~4% of all CpG sites in the stickleback genome. Among pairs of wild-caught populations, we detected 1470 (comparison of 20 versus 6 PSU) and 1158 (20 versus 33 PSU) pop-DMS. The distribution of these sites was random with regard to the genomic features (promoter, exon, intron, and intergenic; 20 versus 6 PSU: *X*^2^_3_ = 3.36, *P* = 0.340; 20 versus 33 PSU: *X*^2^_3_ = 1.61, *P* = 0.656; table S1) and chromosomal regions (fig. S1A). Among these pop-DMS, 1098 (20 versus 6 PSU) and 871 (20 versus 33 PSU) were located close to [<10 kb from transcription start sites (TSS)] or within genes and thereby associated with 655 and 510 genes, respectively. Many of these genes are involved in fundamental biological processes such as DNA repair and strand renaturation, as well as chromosome condensation and separation (fig. S2). Of particular relevance is the enrichment in genes associated with osmoregulatory processes such as ion transport and channel activity, renal water homeostasis and absorption, and urine volume regulation (Fig. 3). Genes associated with ≥10 pop-DMS are listed in Table 1 [for all genes, see table S2 (A and B)].

**Table 1:** Differentially methylated genes across natural populations along a salinity cline. Genes derived from DNA methylation comparisons between natural populations associated with ≥10 pop-DMS [decreased salinity: KIE (20 PSU) versus NYN (6 PSU); increased salinity: KIE (20 PSU) versus SYL (33 PSU)]. Ensembl gene ID and name as well as the position on the chromosome are listed. The numbers refer to the numbers of DMS in the population comparison (wild). These DMS were classified into inducible, inconclusive, and stable sites according to their behavior in a two-generation salinity acclimation experiment with laboratory-bred sticklebacks from the mid-salinity population (20 PSU) exposed to experimental salinity increase or decrease (33 and 6 PSU, respectively). Furthermore, inducible sites were distinguished whether they matched methylation levels of the locally adapted population (expected) or not (opposite). Genes written in bold vary in both population comparisons. We used a Fisher’s exact test to assess whether pop-DMS associated to the same gene are correlated in their response to experimental salinity change (nonrandom distribution among the categories stable, inducible, and inconclusive) and reported corresponding *P* values. For a full table on all genes associated with one or more pop-DMS, see table S2 (A and B).

**Ensembl gene ID**	**Chromosome**	**Start** **position**	**End** **position**	**Gene** **name**	**Wild**	**Inducible**	**Expected** **inducible**	**Opposite** **inducible**	**Stable**	**Inconclusive**	**Fisher’s** **exact (*P*)**
Salinity decrease:											
ENSGACG00000008328	Chr10	12860144	12863850	*si:dkey-166 k12.1*	24	0	0	0	9	15	0.005
**ENSGACG00000019416**	**Chr7**	**4451892**	**4453656**	***HMX1 ortholog***	**17**	**0**	**0**	**0**	**9**	**8**	**0.033**
**ENSGACG00000013229**	**Chr18**	**15327717**	**15352321**		**15**	**0**	**0**	**0**	**3**	**12**	**0.011**
ENSGACG00000017287	Chr3	13454527	13465167	*mmp16b*	12	0	0	0	12	0	0.001
ENSGACG00000017584	Chr3	14690814	14694448	*CCNY*	12	12	12	0	0	0	0.001
ENSGACG00000018249	Chr4	12141625	12143011	*si:ch211-153b23.5*	12	1	1	0	3	8	0.188
ENSGACG00000008034	Chr6	9368187	9380941		11	10	10	0	0	1	0.014
ENSGACG00000009469	Chr1	9166576	9173856	*egln2*	11	0	0	0	11	0	0.001
ENSGACG00000004433	Chr17	2127457	2211376	*igsf21a*	10	10	10	0	0	0	0.003
ENSGACG00000007343	Chr10	10666995	10679875	*col9a2*	10	0	0	0	6	4	0.227
ENSGACG00000018407	Chr4	13828336	13837518	*Sncb*	10	2	2	0	5	3	0.848
Salinity increase:											
ENSGACG00000020323	Chr7	17010160	17011176		23	0	0	0	22	1	<0.001
**ENSGACG00000013229**	**Chr18**	**15327717**	**15352321**		**15**	**10**	**10**	**0**	**1**	**4**	**0.125**
ENSGACG00000013359	Chr11	12960883	12968110	*sec14l1*	15	0	0	0	12	3	0.011
**ENSGACG00000019416**	**Chr7**	**4451892**	**4453656**	***HMX1 ortholog***	**15**	**3**	**3**	**0**	**5**	**7**	**0.745**
ENSGACG00000002948	Chr8	218240	221355	*ddx10*	14	0	0	0	6	8	0.077
ENSGACG00000016350	Chr14	3603545	3604923		14	1	0	1	7	6	0.277
ENSGACG00000006636	Chr18	4780893	4786820	*ZC3H12D*	13	0	0	0	3	10	0.034
ENSGACG00000004667	Chr12	4273498	4286193	*tti1*	12	0	0	0	12	0	0.001
ENSGACG00000015566	Chr2	9043062	9051779	*casc4*	10	0	0	0	10	0	0.003

### Characterizing stable and inducible DNA methylation in a two-generation salinity acclimation experiment

To assess the proportion of inducible DNA methylation, we conducted a two-generation salinity acclimation experiment with laboratory-bred sticklebacks from the mid-salinity population that was subjected to either increased or decreased salinity (Fig. 1). We considered pop-DMS to be noninducible (hereafter referred to as “stable”) when both the within-generational and the transgenerational acclimation groups were not differentially methylated compared to the control group (*q* ≥ 0.0125). On the other hand, if a pop-DMS was differentially methylated between at least one of the acclimation groups (within- and transgenerational) compared to the control group (*q* < 0.0125; methylation difference, ≥15%), then this site was considered inducible. Pop-DMS with a significant *q* value not exceeding the threshold of differential DNA methylation were treated as a separate category (hereafter referred to as inconclusive). After two generations of salinity acclimation, we found that most of the pop-DMS remained stable, regardless of the direction of salinity change (926 pop-DMS, 63% at decreased salinity; 694 pop-DMS, 60% at increased salinity). A smaller number of pop-DMS (13%) were inducible, as they showed a significant change in CpG methylation upon experimental salinity decrease (198 pop-DMS) or increase (148 pop-DMS). An additional 24 and 27% (346 and 316 pop-DMS, respectively) were inconclusive. The number of inducible pop-DMS (13%) derived from comparisons between natural populations was much higher than expected from a random subset of CpG sites across the genome (<1%; 1000 replicates; salinity decrease: *X*^2^_2_ = 1090.7, *P* < 0.001; salinity increase: *X*^2^_2_ = 967.7, *P* < 0.001). This means that pop-DMS are enriched for sites that plastically respond to salinity change, which is expected for populations from different salinities.

### Stable and inducible pop-DMS are associated with different functional gene categories

Gene functions associated with stable pop-DMS (452 and 329 under salinity decrease and increase, respectively) were enriched not only for a number of fundamental biological processes such as DNA repair and chromosome separation (fig. S2) but also for osmoregulatory functions (e.g., ion channel activity; Fig. 3). Furthermore, under increased salinity, many metabolic processes were found among the stable pop-DMS (Fig. 3). Inducible pop-DMS were associated with genes (100 and 82 under salinity decrease and increase, respectively) that were primarily enriched for other osmoregulatory functions regulating, for example, ion transmembrane transport (Fig. 3 and fig. S2). Therefore, stable and inducible pop-DMS affect not only different genes but also different gene ontologies with little overlap (Fig. 3 and fig. S2).

### Assessing the role of inducible DNA methylation in nature

We investigated whether multiple pop-DMS associated with the same gene showed a correlated response to experimental salinity acclimation, which would require that they are nonrandomly distributed among the three categories stable, “inducible,” and “inconclusive.” Accordingly, we found a correlated response for pop-DMS at 13 of 20 genes (genes with more than 10 pop-DMS; Fisher’s exact test, *P* < 0.05; Table 1), which suggests that inducible pop-DMS are predefined and directed.

We then tested whether inducible pop-DMS in the experimental fish became more similar to methylation levels found in natural populations. Of the 198 (decreased salinity) and 148 (increased salinity) inducible pop-DMS, 130 (66%) and 101 (68%), respectively, became more similar to methylation levels of wild population to the corresponding salinity (hereafter referred to as “expected” direction). Conversely, at 68 (34%; decreased salinity) and 47 (32%; increased salinity) inducible pop-DMS, experimental fish showed methylation changes in the opposite direction, reducing the similarity to methylation levels observed in the natural populations (hereafter referred to as “opposite” direction).

**Fig. 4:**
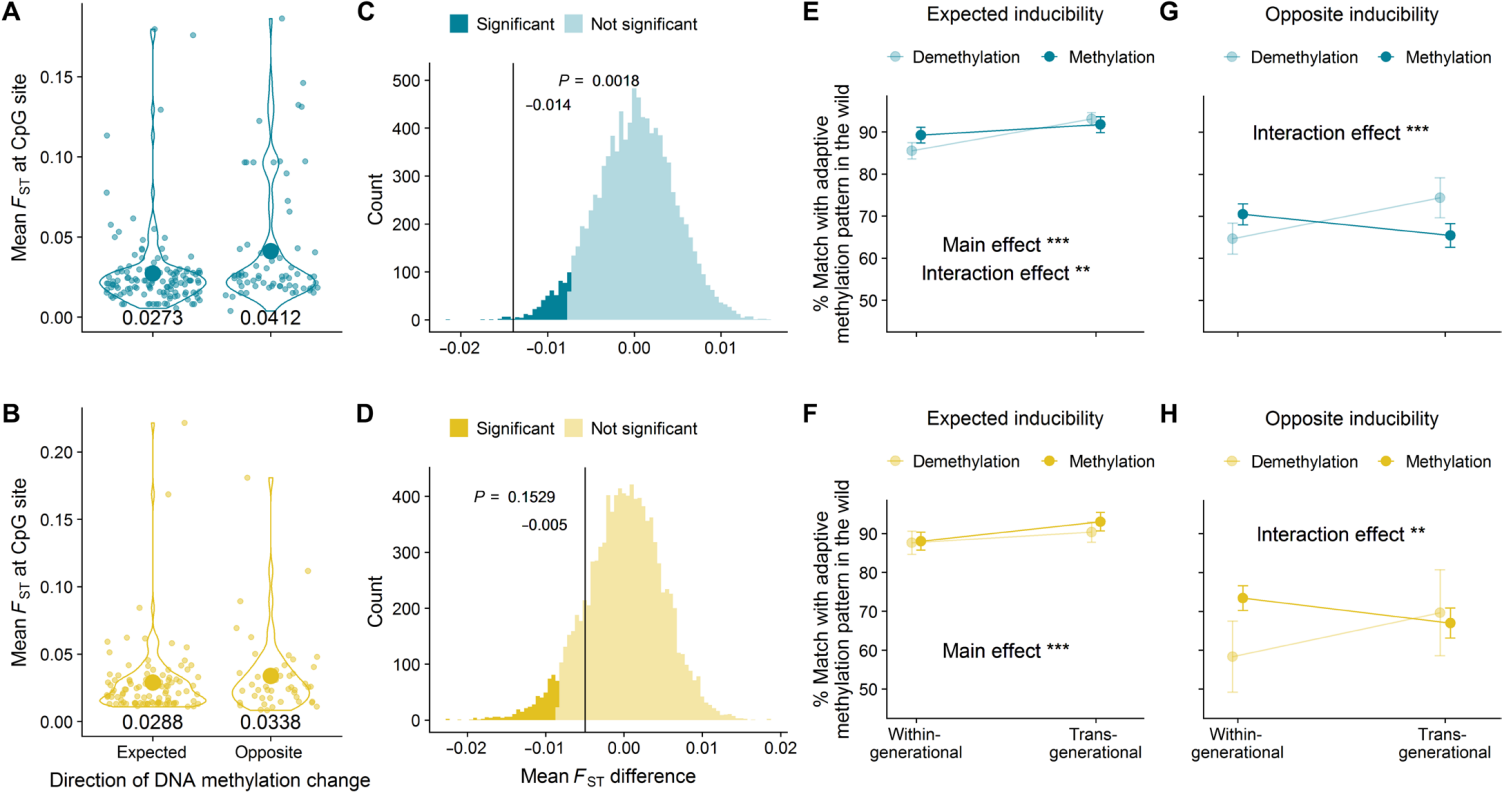
The duration of acclimation (within-generational versus transgenerational) and level of genomic differentiation between populations influence DNA methylation at inducible sites. (**A** and **B**) Mean *F*_ST_ values for inducible pop-DMS (with a ± 5-kb window) under experimental salinity decrease (top; blue) and increase (bottom; yellow) that shifted methylation levels toward the values observed in either the field (expected) or the opposite direction (opposite). A randomization test (with 10,000 bootstraps) was performed for the difference between expected and opposite mean *F*_ST_ value (δ.mean.F_ST_ = expected mean *F*_ST_ – opposite mean *F*_ST_) (**C** and **D**). Under the one-tailed hypothesis of increased genetic differentiation at opposite sites and an α of 0.05, the *P* value was calculated as values smaller than the true difference divided by 10,000 bootstraps. In (**E** to **H**), the *y* axis shows the percentage match between the within- and transgenerational acclimation groups in relation to the methylation differentiation level found in natural populations at inducible pop-DMS. This value was obtained by calculating the difference between the methylation change in the experiment (meth.diff.exp in %; control versus within-generational or control versus transgenerational) and the difference in methylation between natural populations (meth.diff.wild in %) as δ.meth.diff = 100 − (meth.diff.wild − meth.diff.exp). Mean values ± 95% confidence interval are shown for within- and transgenerational acclimation to decreased and increased salinity at expected and opposite inducible sites. Colors refer to the direction of DNA methylation change (hypomethylation or hypermethylation). Values closer to 100 indicate a shift in methylation pattern toward adaptive methylation levels found in natural populations, and asterisks indicate the significance level (***P ≤ 0.001 and **P ≤ 0.01) for the comparison between within- and transgenerational acclimation. “Main effect” refers to an effect of acclimation (within- or transgenerational), and “interaction effect” refers to an interaction of acclimation and methylation direction (hypo- or hypermethylation).

**Fig. 5:**
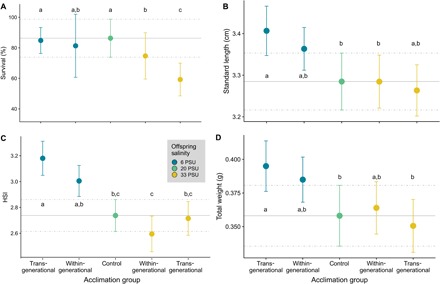
Effects of salinity acclimation on fitness-correlated factors. For all five acclimation groups [control group (20 PSU), within-generational, and transgenerational acclimation to 6 or 33 PSU], survival rates in percent (**A**), standard length in centimeters (**B**), hepatosomatic index (**C**), and total weight in grams (**D**) are displayed. Letters indicate significant differences resulting from Tukey post hoc tests (table S3). HSI, hepatosomatic index.

Why, in a proportion of inducible methylation marks, the similarity between experimental and natural methylation levels was reduced was puzzling. One explanation could be a high level of genomic differentiation between the populations at these sites since genomic variation can have a strong cis-regulatory impact on epigenomic variation and may alter direction and function of methylation marks together (*21*). Thus, we hypothesized that opposite inducible pop-DMS are more often occurring in regions with higher genomic (DNA sequence–based) differentiation, while we anticipated the reverse at expected inducible pop-DMS. Accordingly, we resequenced whole genomes of the same wild-caught individuals that we used for RRBS and calculated the degree of genomic differentiation per inducible pop-DMS as mean *F*_ST_ value (±5-kb window) between populations. In line with our hypothesis, the populations from KIE (20 PSU) and NYN (6 PSU) were genetically more differentiated at opposite inducible pop-DMS than at expected sites (decreased salinity: δ.mean.*F*_ST_ = −0.014, *P* = 0.002; Fig. 4, A and C). A similar, yet not significant, trend was found between the populations from KIE (20 PSU) and SYL (33 PSU) (increased salinity: δ.mean.*F*_ST_ = −0.005, *P* = 0.153; Fig. 4, B and D). An alternative explanation is that not only salinity but also, rather, a combination of environmental cues (i.e., temperature, predation, and food) resulted in the methylation patterns found in the SYL population, which we did not include in our experiment. To understand whether selection has shaped the differences between increased and decreased salinity exposure, we tracked survival rates from fertilized eggs to the 3-month-old offspring and compared them between treatment groups. Mortality differed significantly between the treatment groups [generalized linear mixed model (GLMM), *X*^2^_4_ = 66.159, *P* < 0.001; Fig. 5A and table S3A] with increased mortality under increased salinity, while mortality under decreased salinity was generally low and did not differ from the control group (Fig 5A and table S3A). Hence, while we cannot entirely disregard the effect of selection for increased experimental salinity, the patterns observed at pop-DMS upon reduced salinity are likely the sole result of tolerance mechanisms for salinity change.

### Comparing within- and transgenerational acclimation effects on inducible DNA methylation

To test for adaptive transgenerational plasticity, we evaluated whether salinity acclimation over two, instead of only one, consecutive generations enhances the similarity of inducible pop-DMS with patterns found among wild populations at corresponding salinities. To do so, we calculated the percentage match (δ.meth.diff; Fig. 4, E to H) between the experimental groups and the anticipated adaptive methylation levels of wild populations. In line with our hypothesis, we found that transgenerational compared to within-generational salinity manipulation increased the δ.meth.diff (for expected inducible methylation, decreased salinity: *F*_1,256_ = 30.42, *P* < 0.001; increased salinity: *F*_1,198_ = 10.39, *P* = 0.001; Fig. 4, E and F). Under decreased experimental salinity, we found an interaction of “methylation direction” (hyper- or hypomethylation) and “acclimation” (within- and transgenerational) affecting the δ.meth.diff [analysis of variance (ANOVA), δ.meth.diff ~ methylation direction × acclimation, *F*_1,256_ = 7.69, *P* = 0.006; Fig. 4E]. Specifically, transgenerational acclimation increased the similarity of hypomethylated sites to methylation levels found in natural populations, while hypermethylated sites showed similar values within and across generations (Fig. 4E). While, for expected inducible sites, this effect was only present under decreased salinity, at opposite inducible sites, transgenerational acclimation to decreased and increased salinity elevated the δ.meth.diff at hypomethylated sites (ANOVA, δ.meth.diff ~ methylation direction × acclimation, decreased salinity: *F*_1,132_ = 19.89, *P* < 0.001; increased salinity: *F*_1,90_ = 9.85, *P* = 0.002; Fig. 4, G and H).

To infer the effect of DNA methylation differences on offspring, we compared fitness proxies among control, within-generational, and transgenerational acclimation groups (*22*). Specifically, we assessed the total weight, standard length (SDL), and the hepatosomatic index (HSI) as a proxy for energy reserves in the form of liver glycogen storage. SDL (GLMM, *X*^2^_4_ = 9.965, *P* = 0.041; Fig. 5B and table S3B) and total weight (GLMM, *X*^2^_4_ = 11.518, *P* = 0.021; Fig. 5D and table S3D) differed between treatment groups. Highly significant differences were detected for the HSI (GLMM, *X*^2^_4_ = 22.688, *P* < 0.001; Fig. 5C and table S3C), with elevated HSI observed under decreased salinity compared to fish from the control group. This supports previous findings, showing that osmoregulation at 6 PSU is energetically less demanding than that at higher salinities (*15*, *16*). Under increased salinity, HSI was lower compared to that of fish exposed to decreased salinity in the within-generational acclimation group, while a transgenerational acclimation to increased salinity partially removed this difference. Although not significant, we observed a trend toward higher mean HSI in the transgenerational acclimation group compared to the within-generational acclimation group at the same salinity (Fig. 5C and table S3C).

## DISCUSSION

This study investigated whether two postulated channels of epigenetic inheritance (selection based and detection based) can be identified in natural populations, focusing on salinity adaptation among populations of three-spined sticklebacks. Consistent with expectations for selection-based DNA methylation sites (*7*), we identified pop-DMS between populations that were both enriched for osmoregulatory functions and stable with respect to two generations of experimental salinity manipulation. Phenotypic variation originating from selection-based DNA methylation sites that are expected to have high epimutation rates [~10^−4^ for *Arabidopsis thaliana* (*9*)] could allow populations to explore the fitness landscape faster than under DNA sequence–based genetic variation alone [mutation rate, ~10^−8^ (*5*, *23*)]. Whether and at which rate these randomly emerging epimutations, as predicted for selection-based DNA methylation (*5*), occur in vertebrates remains unresolved. Notwithstanding, the observed enrichment of osmoregulatory gene functions for stable methylation sites (Fig. 3) suggests that they were subject to divergent natural selection, possible in interaction with DNA sequence–based variation. Furthermore, since local adaptation is 10 times more likely to involve changes in gene expression than in amino acid sequence (*24*), it is conceivable that differential DNA methylation and, consequently, regulation of osmoregulatory genes may contribute to local salinity adaptation. In sticklebacks, for instance, immunological adaptation has been shown to be mediated by gene expression (*25*). One of the top candidate genes differentially methylated between populations from 20 and 6 PSU was *eda* (ectodysplasin A), a well-described gene involved in lateral plate formation (*26*). Salinity and calcium are significant drivers of plate morphology (*27*) in proposed conjunction with predation (*28*). Our findings suggest that repeated and parallel selection for the low plated *eda* allele in response to low saline habitats (*29*–*31*), including the Baltic Sea (*14*, *32*), may also involve methylation-related mechanisms. Previous studies have shown that energetic cost for Baltic sticklebacks increases with increasing difference between treatment and isosmotic salinity conditions [~11 PSU (*33*)] (*15*, *16*). In line with these findings, we observed many metabolic processes associated with stable pop-DMS under increased salinity, also reflected in the lower HSI of fish at that salinity. Together, our results on the noninducible fraction of differentially methylated genes are consistent with a role in local salinity adaptation across stickleback populations (Fig. 3; Table 1; fig. S2; and table S2, A and B). These patterns of local adaptation in DNA methylation can have a genomic basis in the form of cis- and trans-acting genomic loci (*21*, *34*). Whether the differential methylation patterns represent an independent mechanism for local adaptation or are rather a consequence of DNA sequence–based genetic differentiation needs further study. Since our experiment only manipulated salinity while keeping all other factors constant, it is possible that some pop-DMS that were stable under salinity change could be inducible by other changing parameters.

With respect to the second postulated information channel, detection-based epigenetic inheritance (*7*), we identified more experimentally inducible pop-DMS than expected by chance. Multiple DMS associated with the same gene showed synchronized responses (Table 1). Furthermore, inducible pop-DMS were associated with different osmoregulatory genes compared to stable pop-DMS. Thus, inducible sites reflect a salinity-mediated plastic response, allowing individuals to regulate their ion balance relative to the seawater medium instantaneously without requiring any further genetic adaptation. More than two-thirds of these inducible pop-DMS became more similar to methylation patterns found in wild population. The similarity of these pop-DMS methylation levels between naturally adapted and experimentally acclimated population increased across generations. Considering the corresponding beneficial phenotypic effects, this strongly suggests that adaptive transgenerational plasticity plays a role in salinity acclimation. Since we used a split-clutch design for the breeding experiment, we can assume that these groups have similar genomic backgrounds. Furthermore, as mortality levels at low salinity remained low and did not differ between treatment groups, we can rule out any effect of selection altering the genotype composition in the groups at decreased salinity.

The induction of methylation sites has been discussed as a potential buffer for environmental changes (*11*, *17*, *35*). We found that the potential for adaptive transgenerational effects, specifically the ability to establish the anticipated adaptive methylation pattern found in the wild, differed among methylation directions (hypo- and hypermethylated sites; Fig. 4, E, G, and H), with a higher potential for transgenerational plasticity at hypomethylated sites. In line with our finding, the spontaneous addition of a methyl group to a cytosine is 2.5 times more likely than the removal (*23*). Methylation reprogramming that includes extensive methylation removal and de novo methylation during gamete formation and zygote development could thus serve as mechanisms to demethylate CpG sites in the transgenerational acclimation group (*36*, *37*).

The genetic background is considered to be an important source for epigenomic variation via cis- and trans-regulatory mechanisms (*21*, *38*, *39*). Thus, we characterized the genomic region surrounding each inducible pop-DMS and quantified the level of population differentiation (*F*_ST_). This analysis revealed a negative correlation between population genetic differentiation and the propensity of the experimental population to approach the methylation level of the low salinity population (NYN) under salinity decrease (Fig. 4, A and C). Here, experimentally induced DNA methylation becomes more similar to the methylation in natural populations only in genomic regions with low genetic differentiation. On the other hand, when experimentally induced methylation differences to the low salinity population increase (Fig. 4, A and C), this occurs in a more divergent genomic background, suggesting that the genome has undergone selection leading to DNA-based local adaptation, rendering epigenetic modifications less relevant (*5*). Under increased salinity, a relationship between genomic differentiation (as *F*_ST_) and methylation direction was inconclusive, suggesting that a combination of environmental cues shaped DNA methylation levels among wild populations at these sites. Overall, these findings emphasize the importance of the genomic background for interpreting DNA methylation patterns.

Together, our study provides the first empirical evidence that stable and inducible DNA methylation in wild animal populations follows predictions from evolutionary theory of selection- and detection-based epigenetic information channels (Fig. 2) (*5*, *7*). While the selection-based information channel assumes random variation from epimutation that is subsequently shaped by selection or drift, the detection-based information channel allows a directional response in the form of transgenerational plasticity. Because the evolutionary implications of these two channels of inheritance are very different, future transgenerational or epigenetic studies should distinguish among both fundamentally different processes. Whether epigenetic marks, such as differentially methylated sites studied here, can permanently be attributed to one of the two categories or rather represent a continuum of stability levels and directionality will need further experimental testing over multiple generations.

## MATERIALS AND METHODS

### Animal welfare

All catches were performed under legal authorization issued by the German Ministry of Energy Transition, Agriculture, Environment, Nature and Digitalization in Schleswig-Holstein (MELUR: V242-7224.121-19), the Danish Ministry of Food, Agriculture and Fisheries of Denmark (case no: 14-7410-000227), the Estonian Ministry of the Environment (Keskkonnaministeerium - eripüügiluba nr 28/2014), and the Swedish Sea and Water Authority (Havs och Vattenmyndigheten). Ethical permission for the experiments required by German law was given by the MELUR: V312-7224.121-19, and the study is also in line with the Institutional Animal Care and Use Committee guidelines.

### Survey and experimental design

For the field survey, we collected juvenile three-spined sticklebacks (*G. aculeatus*; 31.68 ± 14.25 mm) from three different salinity regimes inside and outside the Baltic Sea [SYL, Germany (55°00′58.3″N, 8°26′22.0″E), 33 PSU (*n* = 16); KIE, Germany (54°26′11.8″N, 10°10′20.2″E), 20 PSU (*n* = 16); NYN, Sweden (58°52′44.7″N, 17°56′06.2″E), 6 PSU (*n* = 16)] in September 2014. Fish were immediately euthanized using tricaine methane sulfonate solution (MS222), photographed, measured (length and total weight), and stored in RNAlater solution (24 hours at 7°C, afterward at −20°C). A cut along the ventral side ensured that the RNAlater solution diffused into all tissues. Conserved specimens were later dissected in the laboratory, and gill tissue was separated as the main osmoregulatory organ in fishes. For the acclimation experiment, we collected adult fish from KIE (20 PSU), which were crossed in our facilities at GEOMAR to obtain 10 F1 laboratory-bred families, herein referred to as “parental generation”. At 9 months after hatch, we split each family into three salinity treatment groups of 10 fish each: one at 33 PSU, one at 6 PSU, and one control group at 20 PSU. The salinity transition was performed within 10 days by 3-PSU steps every second day. Over the entire time, each group was fed ad libitum and kept in a 20-liter aquarium connected to one of three filter tanks per salinity treatment. After 5 months under treatment conditions, six pure crosses per salinity treatment group were performed in vitro, herein referred to as “offspring generation” (F2). Offspring and parental generations were kept at 18°C water temperature and a 15:9 light/dark (L/D) cycle. During the past 8 weeks before the F2 crosses, the F1 generation underwent an artificial winter to trigger reproduction (2 weeks at 12°C, 12:12 L/D; 4 weeks at 6°C, 8:16 L/D; 2 weeks at 12°C, 12:12 L/D). Upon fertilization, clutches were split and separated into different treatments (Fig. 1). At 3 months after hatch, laboratory-bred F2 sticklebacks were euthanized using MS222, photographed, and dissected, and their gill tissue was stored in RNAlater solution. The age at sampling matched the estimated age of the wild-caught juveniles (3 months). In addition to the 48 wild-caught individuals from KIE, NYN, and SYL that were used in the above field survey, we sequenced whole genomes from gill tissue of an additional three populations of sticklebacks, namely, from Falsterbo, Sweden (55°24′46.6″N, 12°55′52.3″E; 10 PSU; *n* = 16), Letipea (59°33′07.6”N, 26°36′29.7″E; 4 PSU; *n* = 16), and Barsta (62°51′47.1″N, 18°23′51.0″E; 5 PSU; *n* = 16).

### Mortality and HSI

Mortality was monitored throughout the experiment to account for possible nonrandom effect of selection. Three months after hatch, we assessed the SDL, total weight, and liver weight of the experimental F2 generation and calculated the HSI (HSI = liver weight/total weight × 100), which is a proxy for energy reserves in the form of glycogen storage. We analyzed the effect of treatment (five treatment groups; Fig. 1) on the survival rate per family as a ratio of “alive” versus “dead” fish using glmer implemented in the R package “lme4” (*40*) with binomial error and “crossing” as well as “climate chamber” as random effects. The effect of treatment on HSI, SDL, and total weight was analyzed fitting three individual linear mixed-effect models using lmer in lme4 (*40*) with Gaussian error and crossing as well as tank nested within climate chamber as random effects. Tukey post hoc tests were run using the glht function implemented in the package multicomp (*41*) to identify significant differences between treatment groups.

### DNA extraction

For the field survey, DNA extraction of gill tissue (*n* = 16 individuals per population) was performed using the DNeasy Blood and Tissue Kit (QIAGEN). Further purification of the extracted DNA was done with NucleoSpin gDNA Clean-up (Macherey-Nagel). For laboratory-bred F2 offspring of the two-generation acclimation experiment, dual extraction of whole RNA and DNA was performed from gill tissue (*n* = 11 to 12 individuals per treatment group; Fig. 1) stored in RNAlater solution using the AllPrep DNA/RNA Mini Kit (QIAGEN). Purity and quality of the extracted DNA were estimated using a NanoDrop ND-1000 spectrophotometer (Thermo Fisher Scientific) and a standard agarose gel (1% agarose/tris-acetate-EDTA). DNA concentration was assessed using the Qubit 2.0 Fluorometer (Thermo Fisher Scientific). To obtain a balanced sex ratio (50:50), we determined the gender of the individuals using a sex-specific genetic polymorphism in isocitrate dehydrogenase with a modified protocol from Peichel *et al.* (*42*). For the polymerase chain reaction (PCR) (settings: once 94°C for 3 min; 30 cycles of 94°C for 30 s, 54°C for 20 s, and 72°C for 30 s; once 72°C for 5 min), 1 μl of forward and reverse primer (5 μM) was used with 4.9 μl of water, 1 μl of 10× buffer, 1 μl of deoxynucleotide triphosphate (0.5 μM), and 0.1 μl of DreamTaq (5 U/μl). The resulting PCR products were visualized with a capillary electrophoresis on the 3100 ABI sequencer and a 500 LIZ size standard. While males show a heterogametic signal with two bands [at approximately 300 and 270 base pairs (bp)], females lack the band at 270 bp.

### Library preparation and sequencing (whole-genome sequencing)

For whole-genome sequencing (WGS), the TruSeq Nano DNA (Illumina) library preparation kit was used according to the manufacturer’s protocol by the Sequencing Facility of the IKMB, University of Kiel. Ultrasonication was conducted with a Covaris E220 (Covaris) to shear the input DNA (100 ng per sample and 350-bp insert size). Before the enrichment with a PCR step (8 cycles), fragmented and bead-purified DNA was ligated with adenylate at the blunt 3′ ends (end repair and A-tailing) and with indexing adapters. Fragments were cleaned with MagSi-NGS^prep^ Plus Beads (Steinbrenner). Paired-end sequencing of the quality-controlled and multiplexed libraries was performed on the Illumina HiSeq 4000 platform (2 × 150–bp reads).

### Quality assessment, data filtering, and mapping (WGS)

The command line tools of Picard version 2.7.1 (Broad Institute 2016) was used to (i) reformat the Fastq to uBAM file format and to add further values (read group, etc.) to the SAM header using FastqToSam, (ii) mark the location of adapter sequences using MarkIlluminaAdapters, and (iii) reconvert the sequences to Fastq format with SamToFastq. The stickleback genome (Broad/gasAcu1) was indexed with bwa index and used as a reference for the mapping with bwa mem (*43*) version 07.12-r1044. To retain the meta-information from the uBAMs, we used MergeBamAlignment. Picard was also used to identify duplicates with MarkDuplicates. Basic statistics were generated with CollectWgsMetrics, CollectInsertSizeMetrics, and AlignmentSummaryMetrics and summarized with MultiQC version 1.0.dev0 (*44*). A total number of 4,463,070,154 high-quality reads (mapping quality, >Q20) was mapped resulting in a mean depth of 13.84× (sd. 2.02×) and a mean insert size of 383.07 bp (sd. 9.40 bp; table S3). GATK version 3.7 HaplotypeCaller (*45*) was run to determine the likelihoods of the haplotypes per sample, i.e., to call single-nucleotide polymorphisms (SNPs) and indels (insertion-deletion), which were then processed with GenotypeGVCFs for a joint genotyping. SNPs were selected using hard filters for quality and extracted from the raw genotypes with a combination of the SelectVariants, VariantsToTable, and VariantFiltration commands. VCFtools (*46*) was used in a next step, removing SNPs with a minimum quality score below 20 and a minor allele frequency greater than or equal to 0.0049.

### Library preparation and sequencing (RRBS)

The library preparation for methylation analyses followed the Smallwood and Kelsey RRBS protocol (*47*). A total of 100- to 250-ng purified DNA was digested with the methylation-insensitive Msp I restriction enzyme, which cuts at the “CCGG” motif and thereby enriches for CpG regions. DNA end-repair and A-tailing were conducted, and untailed CEGX spike-in controls (Cambridge Epigenetix) were added. These are DNA oligos of known sequence and with known cytosine modification, which can be used for downstream assessment of bisulfite conversion efficiency. After adapter ligation, bisulfite conversion was conducted using the EZ-96 DNA Methylation-Gold Kit (Zymo Research) according to the manufacturer’s protocol. PCR amplifications with 19 cycles were performed. Quality control of purified PCR products was performed on a 2200 TapeStation System (Agilent), and high-quality libraries were pooled and diversified with 15% PhiX. Single-end sequencing with 100-bp read length was conducted on a HiSeq 2500 sequencer (Illumina).

### Quality assessment, data filtering, and mapping (RRBS)

In total, 106 individuals (48 wild-caught and 58 experimental fish) of balanced sex ratio were DNA-sequenced at an average of 19.8 ± 3.5 million reads for experimental fish and 11.4 ± 2.1 million reads for wild-caught fish (table S4). Demultiplexed Fastq files were quality-checked using FastQC version 0.11.5 (*48*) and MultiQC version 1.3 (*44*). Adapters were removed with cutadapt version 1.9.1 (*49*) using multiple adapter sequences (NNAGATCGGAAGAGCACAC, AGATCGGAAGAGCACAC, and ATCGGAAGAGCACAC) with a minimum overlap of 1 bp between adapter and read. This was necessary to remove primer dimers and avoid false methylation calls systematically caused by the RRBS end-repair step during library preparation, if the end-repair step adds artificial cytosines. Simultaneously, cutadapt was used to trim low-quality bases (-q 20) from the 3′ end and remove trimmed reads shorter than 10 bases. An air bubble during sequencing caused the bases 66 to 72 of 10 tiles of one lane (affecting 12 individuals) to have low-quality values, which were removed in a custom awk script. Two poor-quality individuals (a SYL and a NYN female) did not meet our strict quality requirements (e.g., ≥5 million reads; mapping efficiency, >52%) and showed biases in the proportion of bases per position compared to other individuals (plot in FastQC “per base sequence content”). Therefore, we excluded these two libraries from downstream analysis resulting in 15 instead of 16 individuals from SYL and NYN (Fig. 1). Bisulfite conversion efficiency was assessed from the spike-in controls (Cambridge Epigenetix) using the cegxQC software (*50*). Overall, conversion levels were 2.4 ± 1.8% conversion of methylated cytosines and 99.6 ± 0.5% conversion of unmethylated cytosines, which is in line with expected conversion rates (table S4). We used Bismark version 0.17.0 (*51*) to index the University of California Santa Cruz stickleback reference genome (Broad/gasAcu1) and to generate the bisulfite alignments with Bowtie2 version 2.3.3 at default settings. Bismark was also used to extract the methylation calls. Average mapping efficiency was 63.7 ± 2.4% (table S4).

### Identification of differentially methylated sites

The methylation calls were analyzed in R version 3.4.1 (*52*) using the package methylKit version 1.3.8 (*53*). CpG loci were filtered for a minimum coverage of 10 reads per site. To account for potential PCR bias, we additionally excluded all sites in the 99.9th percentile of coverage. To improve the methylation estimates, we corrected for SNPs, which could have led to a wrong methylation call. The excluded positions were derived with custom-written Perl scripts from C-to-T and G-to-A SNPs with genotype quality of 20 and a minimum allele frequency of 0.005 (see above) from the 96 wild-caught individuals with a combination of custom-written Perl and R scripts using packages from methylKit (*53*) and GenomicRanges (*54*). After normalizing coverage values between samples, using normalizeCoverage implemented in methylKit, we excluded all sites that were present in fewer than nine individuals per treatment group from downstream analysis. As previously shown, sex-specific methylation affects <0.1% of CpG sites on autosomal chromosomes but >5% of CpGs on the sex chromosome (*18*). Therefore, to exclude a potential sex bias, we removed all CpG sites located on the sex chromosomes (chromosome 19), resulting in a high-quality dataset with 525,985 CpG sites. Last, by checking the first six principal components of the resulting principal components analysis and running an ANOVA on the filtered dataset, we confirmed the absence of an effect of sex on global methylation pattern (*F*_124,1_ = 2.611, *P* = 0.109). However, the principal components analysis revealed a bias in methylation pattern by families over all experimental groups. Therefore, to identify differentially methylated CpG sites (DMS) between treatment groups, we performed pairwise comparisons (table S5) fitting a logistic regression model per CpG site with calculateDiffMeth in methylKit using family as covariate for the experimental groups. A chi-square test was applied to assess significance levels of DMS, and *P* values were corrected to *q* values for multiple testing using the sliding linear model method (*55*). In addition, we accounted for multiple use of groups in pairwise comparisons and adjusted the α for the *q* value according to Bonferroni correction to 0.0125 (0.05/4). Ultimately, CpG sites were considered to be differentially methylated with a *q* < 0.0125 and a minimum weighted mean methylation difference of 15%. To ensure that the DMS obtained are not laboratory artifacts, we used calculateDiffMeth implemented in methylKit and compared the wild population from KIE to the experimental control group (KIE population from 20 PSU at 20 PSU). The resulting 11,828 DMS were excluded from the DMS obtained by the pairwise comparisons mentioned above (table S5). DMS were plotted across the genome for the comparison between KIE versus NYN (20 versus 6 PSU; blue fish) and KIE versus SYL (20 versus 33 PSU; yellow fish) using ggplot2 (*56*) and hypoimg (*57*) (fig. S1).

### Assessment of inducibility and gene association of DMS

By comparing wild-caught individuals from the mid-salinity population (20 PSU; KIE) to the populations sampled at low (6 PSU; NYN) and high (33 PSU; SYL) salinity in the field, we obtained 1470 (KIE-NYN) and 1158 (KIE-SYL) pairwise pop-DMS. We first tested whether these pop-DMS distinguishing natural populations are inducible or stable at the respective salinity in the experiment. A pop-DMS was considered stable when the within- and the transgenerational acclimation groups did not significantly differ in methylation to the control group (*q* ≥ 0.0125). On the other hand, pop-DMS were considered inducible when at least one of the acclimation groups was differentially methylated compared to the control group (*q* < 0.0125; methylation difference, ≥15%). pop-DMS with a significant *q* value not exceeding the threshold of differential DNA methylation (15%) will be referred to as inconclusive hereafter. We used a randomization test to ensure that the number of inducible sites obtained did not occur by chance. To this end, we randomly sampled 1470 (KIE-NYN) and 1158 (KIE-SYL) pop-DMS from the complete dataset (1000 replicates). A chi-square test was used to assess whether our observed number of inducible, stable, and inconclusive sites differs from a random set of sites (averaged over replicates). Last, we tested whether the weighted mean methylation difference (meth.diff, in percentage) between wild populations matches the inducible methylation difference by subtracting the “meth.diff” in the experiment (exp) from the meth.diff between wild-caught populations (wild)

δ.meth.diff=100−(meth.diff.wild − meth.diff.exp)

As we subtracted this difference from 100, values closer to 100 indicated higher similarity of experimentally inducible methylation with the postulated adaptive DNA methylation pattern in natural populations. By comparing the “δ.meth.diff” for within- and transgenerational acclimation using an ANOVA, we can assess whether there is a difference in inducibility of methylation to match patterns found in wild-caught populations. All analyses were run separately for decreased (6 PSU; KIE-NYN) and increased (33 PSU; KIE-SYL) salinity.

To detect potential functional associations of the observed changes in DNA methylation state, we classified the genomic region of a pop-DMS on the basis of their nearest TSS using annotateWithGeneParts and getAssociationWithTSS implemented in genomation version 1.4.2 (*58*). We distinguished between promoter (1500 bp upstream and 500 bp downstream of TSS), exon, intron, and intergenic regions. To be associated to a gene, the pop-DMS had to be either inside the gene or, if intergenic, not further than 10 kb away from the TSS. We excluded three pop-DMS that were on a different reference scaffold and then the gene they were associated to on the chrUn linkage group (that merges scaffolds into one large artificial chromosome). Using the genes with associated pop-DMS, we applied a conditional hypergeometric Gene Ontology (GO) term enrichment analysis (false discovery rate–corrected *P* ≤ 0.05) with the Ensembl stickleback annotation dataset “gaculeatus_gene_ensembl,” and all genes that were associated to any sequenced CpG site were used as universe. We identified overrepresented biological processes, molecular functions, and cellular components using the packages GOstats version 2.5 (*59*) and GSEABase version 1.46 (*60*) and corrected for multiple testing using the false discovery rate method implemented in goEnrichment version 1.0 (*61*) in R version 3.6 (*52*). Figures were produced using ggplot2 version 3.2 (*56*).

### Estimation of DNA sequence–based genetic differentiation at differentially methylated sites

To evaluate the genetic differentiation up- and downstream (in sum, 10 kb) of the pop-DMS position, we calculated the mean *F*_ST_ values (≤60% missing data and depth, ≥5) from WGS data of the exact same individuals with vcftools version 0.1.15 (*62*). We hypothesized that inducible CpG positions matching the methylation difference expected from the profile of the wild populations are genetically more similar between the populations than sites that changed in the opposite direction. To test this one-tailed hypothesis, we applied a randomization test (with 10,000 bootstraps) on the mean *F*_ST_ difference between the two groups (expected and opposite)

$$\delta .\text{mean}.F_{\text{ST}}=\text{expected mean}\;F_{\text{ST}}–\text{opposite mean}\;F_{\text{ST}}$$

We plotted the 10,000 delta mean *F*_ST_ values and calculated a *P* value by dividing the proportion of values smaller than the true difference by the number of bootstraps. Figures were produced using ggplot2 version 3.2 (*56*).

## Supplementary Material

aaz1138_SM.pdf

## SUPPLEMENTARY MATERIALS

Supplementary material for this article is available at http://advances.sciencemag.org/cgi/content/full/6/12/eaaz1138/DC1 Fig. S1. Significant DMS throughout the genome for comparison between KIE versus NYN (20 versus 6 PSU; blue fish) and KIE versus SYL (20 versus 33 PSU; yellow fish). Fig. S2. GO terms for biological processes, cellular components, and molecular functions under salinity increase (20 versus 33 PSU; yellow) and decrease (20 versus 6 PSU; blue) associated with pop-DMS. Table S1. Relative distribution of DMS among genomic features. Table S2A. Differentially methylated genes between populations from KIE (20 PSU) and NYN (6 PSU). Table S2B. Differentially methylated genes between populations from KIE (20 PSU) and SYL (33 PSU). Table S3A. Tukey post hoc test results for survival rate. Table S3B. Tukey post hoc test results for SDL. Table S3C. Tukey post hoc test results for HSI. Table S3D. Tukey post hoc test results for total weight. Table S4. Summary statistics for whole-genome resequencing of wild-caught sticklebacks. Table S5A. Summary statistics for the RRBS of experimental fish. Table S5B. Summary statistics for the RRBS of wild-caught fish. Table S6. The number of DMS for each of the two pairwise population comparisons (pop-DMS).

## Appendix

View/request a protocol for this paper from *Bio-protocol*.

## References

[1] Jablonka E., The evolutionary implications of epigenetic inheritance. Interface Focus 7, 20160135 (2017). 28839916 10.1098/rsfs.2016.0135 PMC5566804

[2] Laland K., Uller T., Feldman M., Sterelny K., Müller G. B., Moczek A., Jablonka E., Odling-Smee J., Wray G. A., Hoekstra H. E., Futuyma D. J., Lenski R. E., Mackay T. F. C., Schluter D., Strassmann J. E., Does evolutionary theory need a rethink? Nature 514, 161–164 (2014). 25297418 10.1038/514161a

[3] Lind M. I., Spagopoulou F., Evolutionary consequences of epigenetic inheritance. Heredity 121, 205–209 (2018). 29976958 10.1038/s41437-018-0113-y PMC6082883

[4] Bossdorf O., Richards C. L., Pigliucci M., Epigenetics for ecologists. Ecol. Lett. 11, 106–115 (2008). 18021243 10.1111/j.1461-0248.2007.01130.x

[5] Klironomos F. D., Berg J., Collins S., How epigenetic mutations can affect genetic evolution: Model and mechanism. Bioessays 35, 571–578 (2013). 23580343 10.1002/bies.201200169

[6] Jones P. A., Functions of DNA methylation: Islands, start sites, gene bodies and beyond. Nat. Rev. Genet. 13, 484–492 (2012). 22641018 10.1038/nrg3230

[7] Shea N., Pen I., Uller T., Three epigenetic information channels and their different roles in evolution. J. Evol. Biol. 24, 1178–1187 (2011). 21504495 10.1111/j.1420-9101.2011.02235.x PMC3116147

[8] Kronholm I., Collins S., Epigenetic mutations can both help and hinder adaptive evolution. Mol. Ecol. 25, 1856–1868 (2016). 26139359 10.1111/mec.13296

[9] Schmitz R. J., Schultz M. D., Lewsey M. G., O'Malley R. C., Urich M. A., Libiger O., Schork N. J., Ecker J. R., Transgenerational epigenetic instability is a source of novel methylation variants. Science 334, 369–373 (2011). 21921155 10.1126/science.1212959 PMC3210014

[10] Johannes F., Porcher E., Teixeira F. K., Saliba-Colombani V., Simon M., Agier N., Bulski A., Albuisson J., Heredia F., Audigier P., Bouchez D., Dillmann C., Guerche P., Hospital F., Colot V., Assessing the impact of transgenerational epigenetic variation on complex traits. PLOS Genet. 5, e1000530 (2009). 19557164 10.1371/journal.pgen.1000530 PMC2696037

[11] Ryu T., Veilleux H. D., Donelson J. M., Munday P. L., Ravasi T., The epigenetic landscape of transgenerational acclimation to ocean warming. Nat. Clim. Change 8, 504–509 (2018).

[12] Evans D. H., Cell signaling and ion transport across the fish gill epithelium. J. Exp. Zool. 293, 336–347 (2002). 12115905 10.1002/jez.10128

[13] Reusch T. B. H., Dierking J., Andersson H. C., Bonsdorff E., Carstensen J., Casini M., Czajkowski M., Hasler B., Hinsby K., Hyytiäinen K., Johannesson K., Jomaa S., Jormalainen V., Kuosa H., Kurland S., Laikre L., MacKenzie B. R., Margonski P., Melzner F., Oesterwind D., Ojaveer H., Refsgaard J. C., Sandström A., Schwarz G., Tonderski K., Winder M., Zandersen M., The baltic sea as a time machine for the future coastal ocean. Sci. Adv. 4, eaar8195 (2018). 29750199 10.1126/sciadv.aar8195 PMC5942908

[14] Guo B., DeFaveri J., Sotelo G., Nair A., Merilä J., Population genomic evidence for adaptive differentiation in baltic sea three-spined sticklebacks. BMC Biol. 13, 19 (2015). 25857931 10.1186/s12915-015-0130-8 PMC4410466

[15] Heckwolf M. J., Meyer B. S., Döring T., Eizaguirre C., Reusch T. B. H., Transgenerational plasticity and selection shape the adaptive potential of sticklebacks to salinity change. Evol. Appl. 11, 1873–1885 (2018). 30459835 10.1111/eva.12688 PMC6231470

[16] DeFaveri J., Merilä J., Local adaptation to salinity in the three-spined stickleback? J. Evol. Biol. 27, 290–302 (2014). 24330503 10.1111/jeb.12289

[17] Shama L. N. S., Mark F. C., Strobel A., Lokmer A., John U., Mathias Wegner K., Transgenerational effects persist down the maternal line in marine sticklebacks: Gene expression matches physiology in a warming ocean. Evol. Appl. 9, 1096–1111 (2016). 27695518 10.1111/eva.12370 PMC5039323

[18] Metzger D. C. H., Schulte P. M., The DNA methylation landscape of stickleback reveals patterns of sex chromosome evolution and effects of environmental salinity. Genome Biol. Evol. 10, 775–785 (2018). 29420714 10.1093/gbe/evy034 PMC5841383

[19] Artemov A. V., Mugue N. S., Rastorguev S. M., Zhenilo S., Mazur A. M., Tsygankova S. V., Boulygina E. S., Kaplun D., Nedoluzhko A. V., Medvedeva Y. A., Prokhortchouk E. B., Genome-wide DNA methylation profiling reveals epigenetic adaptation of stickleback to marine and freshwater conditions. Mol. Biol. Evol. 34, 2203–2213 (2017). 28873953 10.1093/molbev/msx156

[20] Bird A., Perceptions of epigenetics. Nature 447, 396–398 (2007). 17522671 10.1038/nature05913

[21] Taudt A., Colomé-Tatché M., Johannes F., Genetic sources of population epigenomic variation. Nat. Rev. Genet. 17, 319–332 (2016). 27156976 10.1038/nrg.2016.45

[22] Dufresne F., FitzGerald G. J., Lachance S., Age and size-related differences in reproductive success and reproductive costs in threespine sticklebacks (*Gasterosteus aculeatus*). Behav. Ecol. 1, 140–147 (1990).

[23] van der Graaf A., Wardenaar R., Neumann D. A., Taudt A., Shaw R. G., Jansen R. C., Schmitz R. J., Colomé-Tatché M., Johannes F., Rate, spectrum, and evolutionary dynamics of spontaneous epimutations. Proc. Natl. Acad. Sci. U.S.A. 112, 6676–6681 (2015). 25964364 10.1073/pnas.1424254112 PMC4450394

[24] Fraser H. B., Gene expression drives local adaptation in humans. Genome Res. 23, 1089–1096 (2013). 23539138 10.1101/gr.152710.112 PMC3698502

[25] Lenz T. L., Eizaguirre C., Rotter B., Kalbe M., Milinski M., Exploring local immunological adaptation of two stickleback ecotypes by experimental infection and transcriptome-wide digital gene expression analysis. Mol. Ecol. 22, 774–786 (2013). 22971109 10.1111/j.1365-294X.2012.05756.x PMC3579235

[26] Colosimo P. F., Hosemann K. E., Balabhadra S., Villarreal G. Jr., Dickson M., Grimwood J., Schmutz J., Myers R. M., Schluter D., Kingsley D. M., Widespread parallel evolution in sticklebacks by repeated fixation of ectodysplasin alleles. Science 307, 1928–1933 (2005). 15790847 10.1126/science.1107239

[27] Spence R., Wootton R. J., Przybylski M., Zięba G., Macdonald K., Smith C., Calcium and salinity as selective factors in plate morph evolution of the three-spined stickleback (*Gasterosteus aculeatus*). J. Evol. Biol. 25, 1965–1974 (2012). 22862551 10.1111/j.1420-9101.2012.02585.x

[28] Paccard A., Wasserman B. A., Hanson D., Astorg L., Durston D., Kurland S., Apgar T. M., El-Sabaawi R. W., Palkovacs E. P., Hendry A. P., Barrett R. D. H., Adaptation in temporally variable environments: Stickleback armor in periodically breaching bar-built estuaries. J. Evol. Biol. 31, 735–752 (2018). 29532568 10.1111/jeb.13264

[29] Ferchaud A.-L., Pedersen S. H., Bekkevold D., Jian J., Niu Y., Hansen M. M., A low-density SNP array for analyzing differential selection in freshwater and marine populations of threespine stickleback (*Gasterosteus aculeatus*). BMC Genomics 15, 867 (2014). 25286752 10.1186/1471-2164-15-867 PMC4196021

[30] Hohenlohe P. A., Bassham S., Etter P. D., Stiffler N., Johnson E. A., Cresko W. A., Population genomics of parallel adaptation in threespine stickleback using sequenced RAD tags. PLOS Genet. 6, e1000862 (2010). 20195501 10.1371/journal.pgen.1000862 PMC2829049

[31] Terekhanova N. V., Logacheva M. D., Penin A. A., Neretina T. V., Barmintseva A. E., Bazykin G. A., Kondrashov A. S., Mugue N. S., Fast evolution from precast bricks: Genomics of young freshwater populations of threespine stickleback *Gasterosteus aculeatus*. PLOS Genet. 10, e1004696 (2014). 25299485 10.1371/journal.pgen.1004696 PMC4191950

[32] DeFaveri J., Jonsson P. R., Merilä J., Heterogeneous genomic differentiation in marine threespine sticklebacks: Adaptation along an environmental gradient. Evolution 67, 2530–2546 (2013). 24033165 10.1111/evo.12097

[33] Schaarschmidt T., Meyer E., Jürss K., A comparison of transport-related gill enzyme activities and tissue-specific free amino acid concentrations of Baltic Sea (brackish water) and freshwater threespine sticklebacks, gasterosteus aculeatus, after salinity and temperature acclimation. Mar. Biol. 135, 689–697 (1999).

[34] Dubin M. J., Zhang P., Meng D., Remigereau M.-S., Osborne E. J., Paolo Casale F., Drewe P., Kahles A., Jean G., Vilhjálmsson B., Jagoda J., Irez S., Voronin V., Song Q., Long Q., Rätsch G., Stegle O., Clark R. M., Nordborg M., DNA methylation in *Arabidopsis* has a genetic basis and shows evidence of local adaptation. eLife 4, e05255 (2015). 25939354 10.7554/eLife.05255 PMC4413256

[35] Torda G., Donelson J. M., Aranda M., Barshis D. J., Bay L., Berumen M. L., Bourne D. G., Cantin N., Foret S., Matz M., Miller D. J., Moya A., Putnam H. M., Ravasi T., van Oppen M. J. H., Thurber R. V., Vidal-Dupiol J., Voolstra C. R., Watson S.-A., Whitelaw E., Willis B. L., Munday P. L., Rapid adaptive responses to climate change in corals. Nat. Clim. Change 7, 627–636 (2017).

[36] Potok M. E., Nix D. A., Parnell T. J., Cairns B. R., Reprogramming the maternal zebrafish genome after fertilization to match the paternal methylation pattern. Cell 153, 759–772 (2013). 23663776 10.1016/j.cell.2013.04.030 PMC4030421

[37] Labbé C., Robles V., Herraez M. P., Epigenetics in fish gametes and early embryo. Aquaculture 472, 93–106 (2017).

[38] Gertz J., Varley K. E., Reddy T. E., Bowling K. M., Pauli F., Parker S. L., Kucera K. S., Willard H. F., Myers R. M., Analysis of DNA methylation in a three-generation family reveals widespread genetic influence on epigenetic regulation. PLOS Genet. 7, e1002228 (2011). 21852959 10.1371/journal.pgen.1002228 PMC3154961

[39] Gibbs J. R., van der Brug M. P., Hernandez D. G., Traynor B. J., Nalls M. A., Lai S.-L., Arepalli S., Dillman A., Rafferty I. P., Troncoso J., Johnson R., Zielke H. R., Ferrucci L., Longo D. L., Cookson M. R., Singleton A. B., Abundant quantitative trait loci exist for DNA methylation and gene expression in human brain. PLOS Genet. 6, e1000952 (2010). 20485568 10.1371/journal.pgen.1000952 PMC2869317

[40] Bates D., Maechler M., Bolker B., Walker S., lme4: Linear mixed-effects models using Eigen and S4. R package version 1, 1–23 (2014).

[41] Hothorn T., Bretz F., Westfall P., Simultaneous inference in general parametric models. Biom. J. 50, 346–363 (2008). 18481363 10.1002/bimj.200810425

[42] Peichel C. L., Ross J. A., Matson C. K., Dickson M., Grimwood J., Schmutz J., Myers R. M., Mori S., Schluter D., Kingsley D. M., The master sex-determination locus in threespine sticklebacks is on a nascent Y chromosome. Curr. Biol. 14, 1416–1424 (2004). 15324658 10.1016/j.cub.2004.08.030

[43] Li H., Durbin R., Fast and accurate short read alignment with Burrows–Wheeler transform. Bioinformatics 25, 1754–1760 (2009). 19451168 10.1093/bioinformatics/btp324 PMC2705234

[44] Ewels P., Magnusson M., Lundin S., Käller M., MultiQC: Summarize analysis results for multiple tools and samples in a single report. Bioinformatics 32, 3047–3048 (2016). 27312411 10.1093/bioinformatics/btw354 PMC5039924

[45] McKenna A., Hanna M., Banks E., Sivachenko A., Cibulskis K., Kernytsky A., Garimella K., Altshuler D., Gabriel S., Daly M., DePristo M. A., The genome analysis toolkit: A MapReduce framework for analyzing next-generation DNA sequencing data. Genome Res. 20, 1297–1303 (2010). 20644199 10.1101/gr.107524.110 PMC2928508

[46] Danecek P., Auton A., Abecasis G., Albers C. A., Banks E., DePristo M. A., Handsaker R. E., Lunter G., Marth G. T., Sherry S. T., McVean G., Durbin R.; 1000 Genomes Project Analysis Group, The variant call format and VCFtools. Bioinformatics 27, 2156–2158 (2011). 21653522 10.1093/bioinformatics/btr330 PMC3137218

[47] S. A. Smallwood, G. Kelsey, Genome-wide analysis of DNA methylation in low cell numbers by reduced representation bisulfite sequencing, in *Genomic Imprinting: Methods and Protocols*, N. Engel, Ed. (Humana Press, 2012), pp. 187–197. 10.1007/978-1-62703-011-3_12 22907498

[48] S. Andrews, FastQC: A quality control tool for high throughput sequence data (2010); www.bioinformatics.babraham.ac.uk/projects/fastqc.

[49] Martin M., Cutadapt removes adapter sequences from high-throughput sequencing reads. EMBnet. Journal 17, 10–12 (2011).

[50] CEGX Bioinfomatics Team, *Cambridge Epigenetix (CEGX)* (Babraham Research Campus, Cambridge, 2015).

[51] Krueger F., Andrews S. R., Bismark: A flexible aligner and methylation caller for Bisulfite-Seq applications. Bioinformatics 27, 1571–1572 (2011). 21493656 10.1093/bioinformatics/btr167 PMC3102221

[52] R Core Team, *R: A Language and Environment for Statistical Computing* (R Foundation for Statistical Computing, Vienna, Austria, 2017).

[53] Akalin A., Kormaksson M., Li S., Garrett-Bakelman F. E., Figueroa M. E., Melnick A., Mason C. E., methylKit: A comprehensive R package for the analysis of genome-wide DNA methylation profiles. Genome Biol. 13, R87 (2012). 23034086 10.1186/gb-2012-13-10-r87 PMC3491415

[54] Lawrence M., Huber W., Pagès H., Aboyoun P., Carlson M., Gentleman R., Morgan M. T., Carey V. J., Software for computing and annotating genomic ranges. PLOS Comput. Biol. 9, e1003118 (2013). 23950696 10.1371/journal.pcbi.1003118 PMC3738458

[55] Wang H.-Q., Tuominen L. K., Tsai C.-J., SLIM: A sliding linear model for estimating the proportion of true null hypotheses in datasets with dependence structures. Bioinformatics 27, 225–231 (2011). 21098430 10.1093/bioinformatics/btq650

[56] H. Wickham, *ggplot2: Elegant Graphics for Data Analysis* (Springer, 2016).

[57] K. Hench, hypoimg (2019); https://github.com/k-hench/hypoimg.

[58] Akalin A., Franke V., Vlahoviček K., Mason C. E., Schübeler D., Genomation: A toolkit to summarize, annotate and visualize genomic intervals. Bioinformatics 31, 1127–1129 (2014). 25417204 10.1093/bioinformatics/btu775

[59] Falcon S., Gentleman R., Using GOstats to test gene lists for GO term association. Bioinformatics 23, 257–258 (2006). 17098774 10.1093/bioinformatics/btl567

[60] M. Morgan, S. Falcon, R. Gentleman, GSEABase: Gene set enrichment data structures and methods (2019).

[61] A. Hallab, goEnrichment: Helper functions to compute GO enrichment tests using GOstats and GSEABase (2015).

[62] Weir B. S., Cockerham C. C., Estimating *F*-statistics for the analysis of population structure. Evolution 38, 1358–1370 (1984). 28563791 10.1111/j.1558-5646.1984.tb05657.x

[63] Meier H. E. M., Baltic sea climate in the late twenty-first century: A dynamical downscaling approach using two global models and two emission scenarios. Clim. Dynam. 27, 39–68 (2006).

