# Supplementary material for: Two different epigenetic information channels in wild three-spined sticklebacks are involved in salinity adaptation
Source: Sci Adv. 2020 Mar 20;6(12):eaaz1138. doi: 10.1126/sciadv.aaz1138 (PMC7083608; doi:10.1126/sciadv.aaz1138)
Supplement: aaz1138_SM.pdf [file aaz1138_SM.pdf]

Science Advances
AAAS

advances.sciencemag.org/cgi/content/full/6/12/eaaz1138/DC1

Supplementary Materials for

Two different epigenetic information channels in wild three-spined sticklebacks are involved in salinity adaptation

Melanie J. Heckwolf*, Britta S. Meyer, Robert Häsler, Marc P. Höppner, Christophe Eizaguirre, Thorsten B. H. Reusch

*Corresponding author. Email: mheckwolf@geomar.de

Published 20 March 2020, *Sci. Adv.* **6**, eaaz1138 (2020)
DOI: 10.1126/sciadv.aaz1138

This PDF file includes:

Fig. S1. Significant DMS throughout the genome for comparison between KIE versus NYN (20 versus 6 PSU; blue fish) and KIE versus SYL (20 versus 33 PSU; yellow fish).
Fig. S2. GO terms for biological processes, cellular components, and molecular functions under salinity increase (20 versus 33 PSU; yellow) and decrease (20 versus 6 PSU; blue) associated with pop-DMS.
Table S1. Relative distribution of DMS among genomic features.
Table S2A. Differentially methylated genes between populations from KIE (20 PSU) and NYN (6 PSU).
Table S2B. Differentially methylated genes between populations from KIE (20 PSU) and SYL (33 PSU).
Table S3A. Tukey post hoc test results for survival rate.
Table S3B. Tukey post hoc test results for SDL.
Table S3C. Tukey post hoc test results for HSI.
Table S3D. Tukey post hoc test results for total weight.
Table S4. Summary statistics for whole-genome resequencing of wild-caught sticklebacks.
Table S5A. Summary statistics for the RRBS of experimental fish.
Table S5B. Summary statistics for the RRBS of wild-caught fish.
Table S6. The number of DMS for each of the two pairwise population comparisons (pop-DMS).

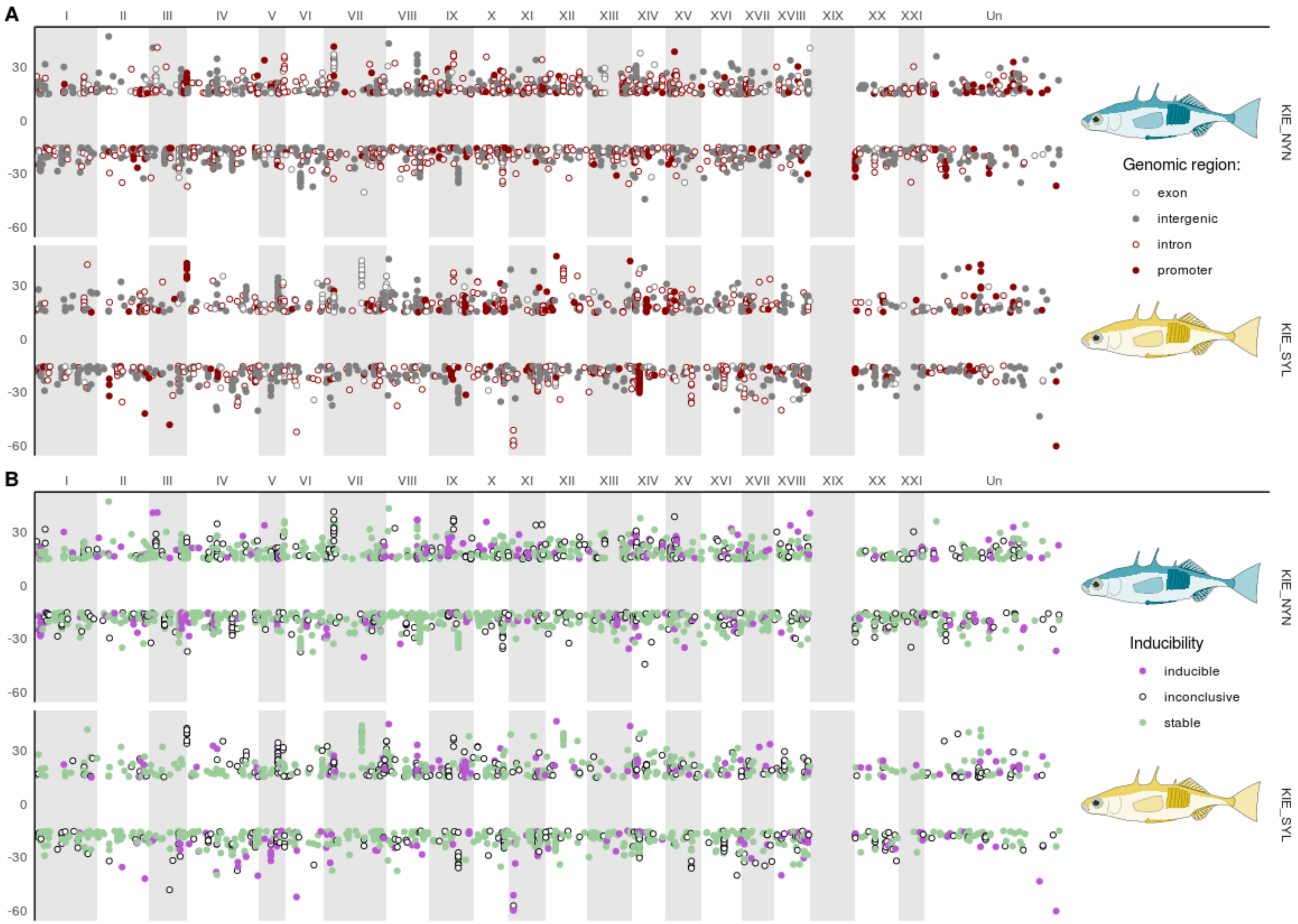

Fig. S1. Significant DMS throughout the genome for comparison between KIE versus NYN (20 versus 6 PSU; blue fish) and KIE versus

SYL (20 versus 33 PSU; yellow fish). Each DMS is one dot and the y axis represents the methylation difference in percent (%). DMS > 0 are hypermethylated in Nynäshamn / Sylt compared to Kiel and DMS < 0 are hypomethylated in Nynäshamn / Sylt compared to Kiel. No DMS are shown on the sex chromosome (number 19), since this was filtered out to reduce the sex bias (see Methods for details). The “chromosome unknown” (Un) represents a size-sorted collection of un-assembled scaffolds. Figure S1A and S1B show the same DMS. In S1A the DMS are colored according to their genomic location (exon, intergenic, intron, promoter) and in S1B according to their stability (stable, inconclusive, experimentally inducible).

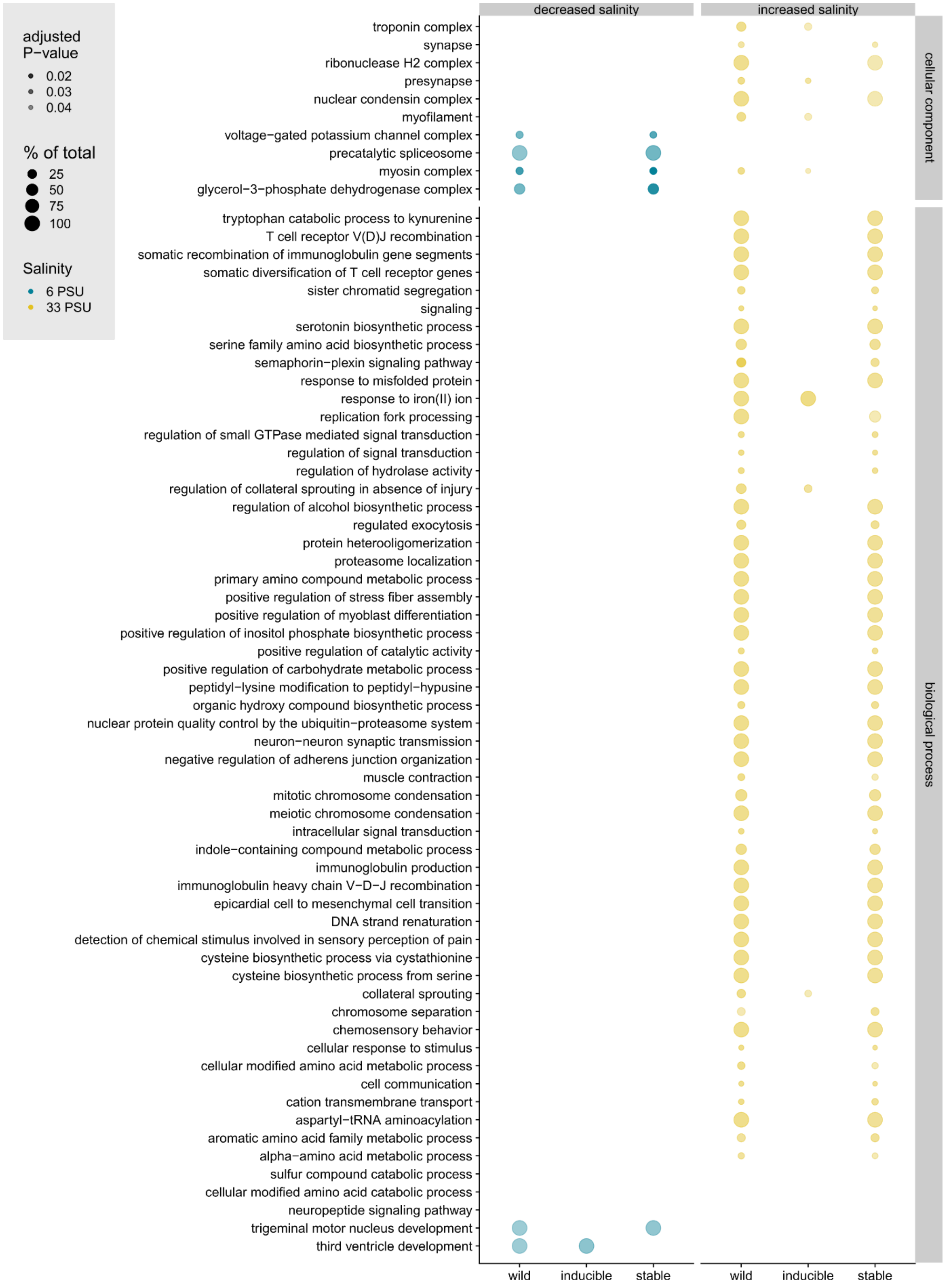
adjusted P−value
0.02
0.03
0.04
% of total
25
50
75
100
Salinity
6 PSU
33 PSU
decreased salinity
increased salinity
cellular component
biological process
troponin complex
synapse
ribonuclease H2 complex
presynapse
nuclear condensin complex
myofilament
voltage−gated potassium channel complex
precatalytic spliceosome
myosin complex
glycerol−3−phosphate dehydrogenase complex
tryptophan catabolic process to kynurenine
T cell receptor V(D)J recombination
somatic recombination of immunoglobulin gene segments
somatic diversification of T cell receptor genes
sister chromatid segregation
signaling
serotonin biosynthetic process
serine family amino acid biosynthetic process
semaphorin−plexin signaling pathway
response to misfolded protein
response to iron(II) ion
replication fork processing
regulation of small GTPase mediated signal transduction
regulation of signal transduction
regulation of hydrolase activity
regulation of collateral sprouting in absence of injury
regulation of alcohol biosynthetic process
regulated exocytosis
protein heterooligomerization
proteasome localization
primary amino compound metabolic process
positive regulation of stress fiber assembly
positive regulation of myoblast differentiation
positive regulation of inositol phosphate biosynthetic process
positive regulation of catalytic activity
positive regulation of carbohydrate metabolic process
peptidyl−lysine modification to peptidyl−hypusine
organic hydroxy compound biosynthetic process
nuclear protein quality control by the ubiquitin−proteasome system
neuron−neuron synaptic transmission
negative regulation of adherens junction organization
muscle contraction
mitotic chromosome condensation
meiotic chromosome condensation
intracellular signal transduction
indole−containing compound metabolic process
immunoglobulin production
immunoglobulin heavy chain V−D−J recombination
epicardial cell to mesenchymal cell transition
DNA strand renaturation
detection of chemical stimulus involved in sensory perception of pain
cysteine biosynthetic process via cystathionine
cysteine biosynthetic process from serine
collateral sprouting
chromosome separation
chemosensory behavior
cellular response to stimulus
cellular modified amino acid metabolic process
cell communication
cation transmembrane transport
aspartyl−tRNA aminoacylation
aromatic amino acid family metabolic process
alpha−amino acid metabolic process
sulfur compound catabolic process
cellular modified amino acid catabolic process
neuropeptide signaling pathway
trigeminal motor nucleus development
third ventricle development
wild
inducible
stable
wild
inducible
stable

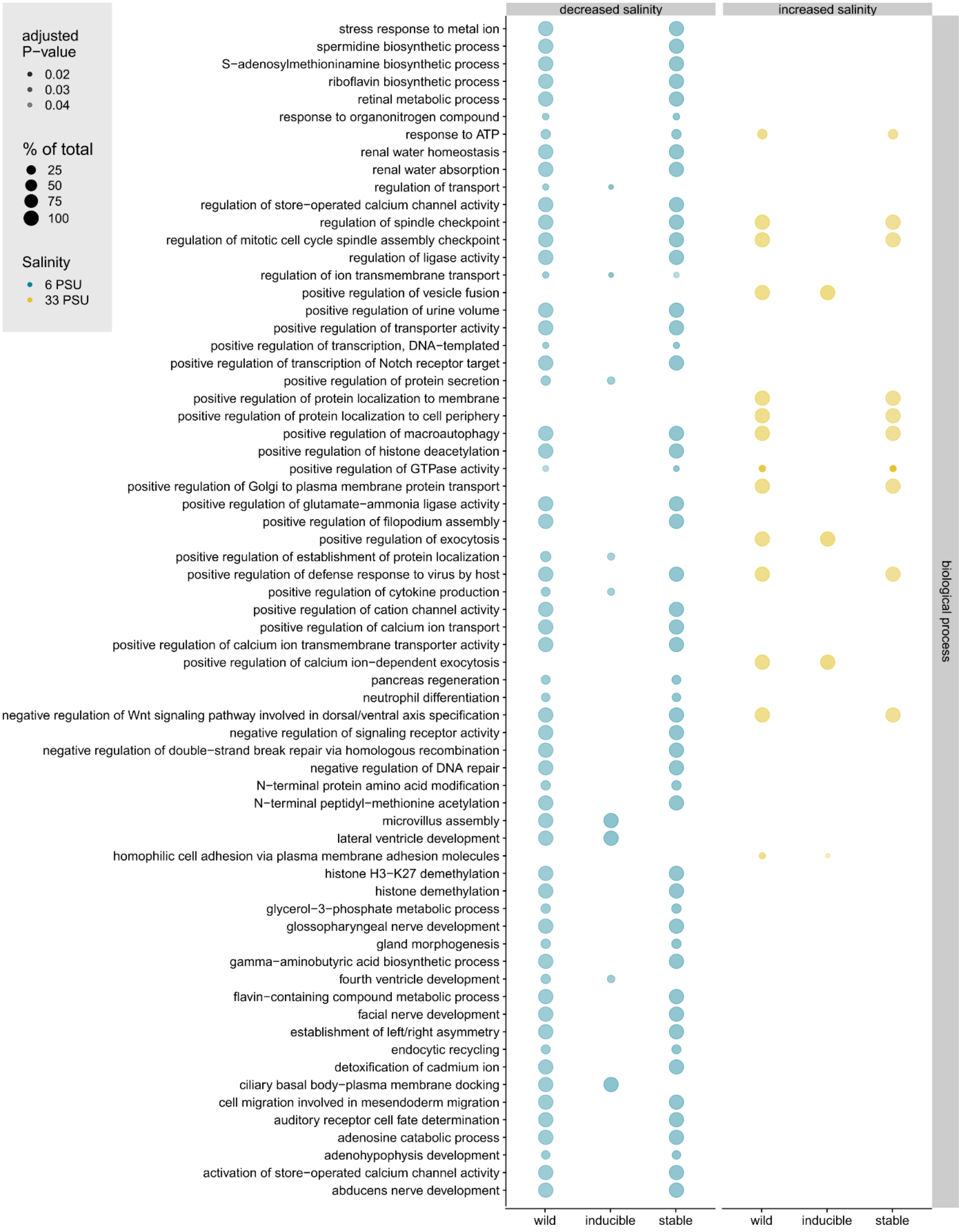

adjusted P−value
0.02
0.03
0.04
% of total
25
50
75
100
Salinity
6 PSU
33 PSU
decreased salinity
increased salinity
stress response to metal ion
spermidine biosynthetic process
S−adenosylmethioninamine biosynthetic process
riboflavin biosynthetic process
retinal metabolic process
response to organonitrogen compound
response to ATP
renal water homeostasis
renal water absorption
regulation of transport
regulation of store−operated calcium channel activity
regulation of spindle checkpoint
regulation of mitotic cell cycle spindle assembly checkpoint
regulation of ligase activity
regulation of ion transmembrane transport
positive regulation of vesicle fusion
positive regulation of urine volume
positive regulation of transporter activity
positive regulation of transcription, DNA−templated
positive regulation of transcription of Notch receptor target
positive regulation of protein secretion
positive regulation of protein localization to membrane
positive regulation of protein localization to cell periphery
positive regulation of macroautophagy
positive regulation of histone deacetylation
positive regulation of GTPase activity
positive regulation of Golgi to plasma membrane protein transport
positive regulation of glutamate−ammonia ligase activity
positive regulation of filopodium assembly
positive regulation of exocytosis
positive regulation of establishment of protein localization
positive regulation of defense response to virus by host
positive regulation of cytokine production
positive regulation of cation channel activity
positive regulation of calcium ion transport
positive regulation of calcium ion transmembrane transporter activity
positive regulation of calcium ion−dependent exocytosis
pancreas regeneration
neutrophil differentiation
negative regulation of Wnt signaling pathway involved in dorsal/ventral axis specification
negative regulation of signaling receptor activity
negative regulation of double−strand break repair via homologous recombination
negative regulation of DNA repair
N−terminal protein amino acid modification
N−terminal peptidyl−methionine acetylation
microvillus assembly
lateral ventricle development
homophilic cell adhesion via plasma membrane adhesion molecules
histone H3−K27 demethylation
histone demethylation
glycerol−3−phosphate metabolic process
glossopharyngeal nerve development
gland morphogenesis
gamma−aminobutyric acid biosynthetic process
fourth ventricle development
flavin−containing compound metabolic process
facial nerve development
establishment of left/right asymmetry
endocytic recycling
detoxification of cadmium ion
ciliary basal body−plasma membrane docking
cell migration involved in mesendoderm migration
auditory receptor cell fate determination
adenosine catabolic process
adenohypophysis development
activation of store−operated calcium channel activity
abducens nerve development
wild
inducible
stable
wild
inducible
stable
biological process

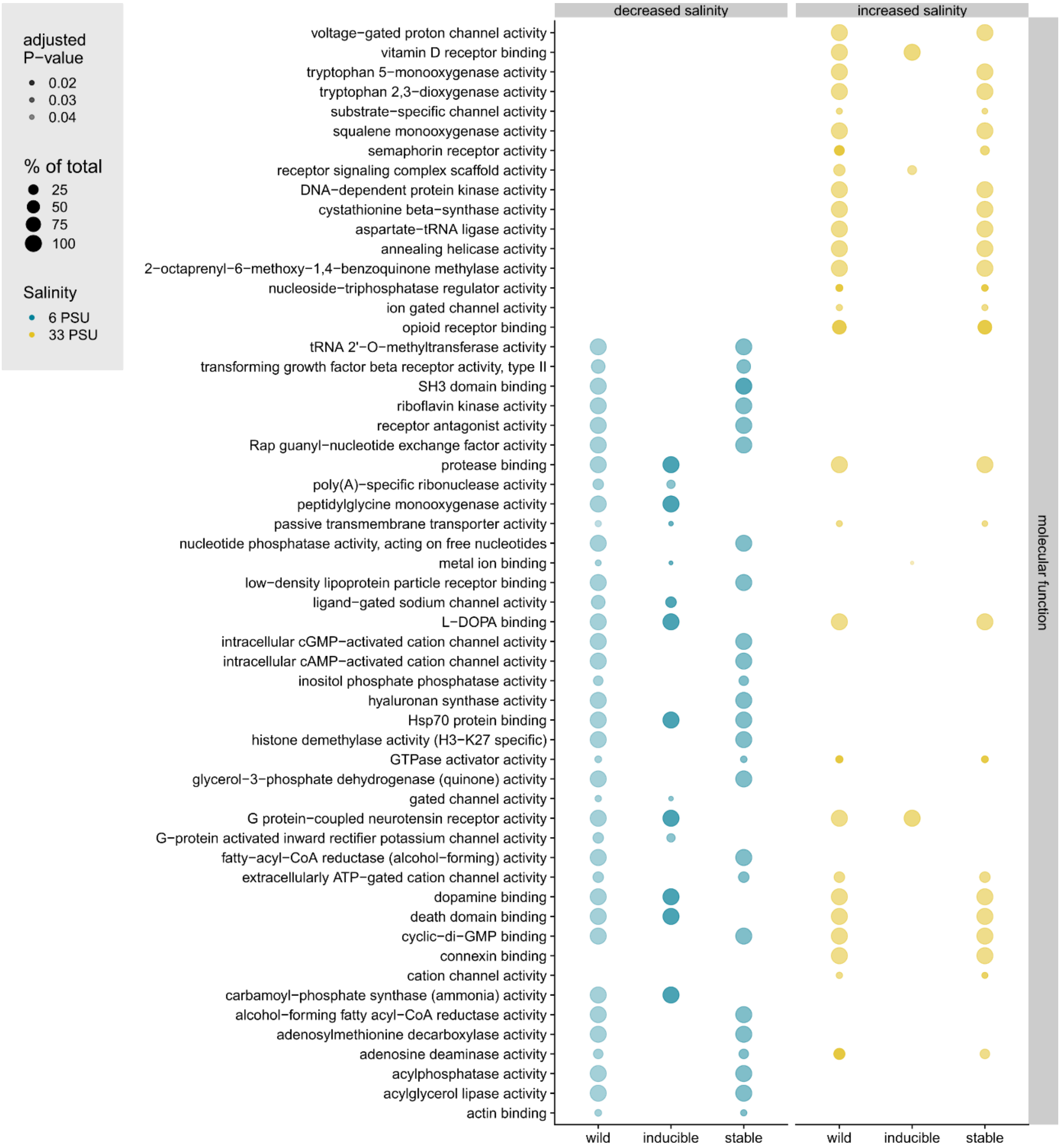

Fig. S2. GO terms for biological processes, cellular components, and molecular functions under salinity increase (20 versus 33 PSU; yellow) and decrease (20 versus 6 PSU; blue) associated with pop-DMS. The graph is split into GO terms associated with pop-DMS from natural stickleback populations across a salinity cline (wild) and their experimental inducibility (inducible and stable) in a two-generation acclimation experiment. The size of the circles refers to the number of genes of this term present in our groups (in %) and the transparency to the *P*-value (darker circles refer to a lower *P*-value).

Table S1. Relative distribution of DMS among genomic features. Given are DMS between field populations (KIE vs. NYN for 20 vs. 6 PSU and KIE vs. SYL for 20 vs. 33 PSU), as well as experimentally inducible and stable sites for increased and decreased salinity separately. 525,985 CpGs were sequenced.

Comparison		Genomic feature (%)			
		exon	intergenic	intron	promoter
DMS under salinity decrease	KIE vs. NYN	11.2	45.9	31.5	11.4
	inducible	9.1	49.5	30.3	11.1
	stable	11.8	44.8	33.5	9.9
DMS under salinity increase	KIE vs. SYL	13.6	43.3	30.1	13.0
	inducible	9.5	42.5	31.8	16.2
	stable	13.0	42.9	32.6	11.5
CpGs sequenced in total		15.1	40.7	25.4	18.8

Table S2A. Differentially methylated genes between populations from KIE (20 PSU) and NYN (6 PSU). For genes associated with DMS, Ensembl gene ID and gene name as well as the position on the chromosome are listed. The numbers refer to the numbers of DMS in the population comparison (wild), these DMS were classified into ‘inducible’, ‘inconclusive’ and ‘stable’ sites according to their behavior in a two-generation salinity acclimation experiment with laboratory bred sticklebacks from Kiel (20 PSU) exposed to experimental salinity decrease (6 PSU) (see Methods for details). Further, inducible sites were distinguished whether they matched methylation levels of the locally adapted population (‘*expected*’) or not (‘*opposite*’).

Ensembl gene ID	chromosome	start position	end position	gene name	wild	inducible	expected inducible	opposite inducible	stable	inconclusive
ENSGACG00000008328	ChrX	12860144	12863850	si:dkey-166k12.1	24	0	0	0	9	15
ENSGACG00000019416	ChrVII	4451892	4453656	HMX1 orthologue	17	0	0	0	9	8
ENSGACG00000013229	ChrXVIII	15327717	15352321		15	0	0	0	3	12
ENSGACG00000017287	ChrIII	13454527	13465167	mmp16b	12	0	0	0	12	0
ENSGACG00000017584	ChrIII	14690814	14694448	CCNY (1 of many)	12	12	12	0	0	0
ENSGACG00000018249	ChrIV	12141625	12143011	si:ch211-153b23.5 (1 of many)	12	1	1	0	3	8
ENSGACG00000008034	ChrVI	9368187	9380941		11	10	10	0	0	1
ENSGACG00000009469	ChrI	9166576	9173856	egln2	11	0	0	0	11	0
ENSGACG00000004433	ChrXVII	2127457	2211376	igsf21a	10	10	10	0	0	0
ENSGACG00000007343	ChrX	10666995	10679875	col9a2	10	0	0	0	6	4
ENSGACG00000018407	ChrIV	13828336	13837518	sncb	10	2	2	0	5	3
ENSGACG00000011118	ChrI	12219197	12219822		9	0	0	0	6	3
ENSGACG00000011999	ChrXIII	13539291	13591502	pbx3b	9	6	0	6	2	1
ENSGACG00000016734	ChrIV	3512927	3533277	ANKRD50	9	0	0	0	9	0
ENSGACG00000002963	ChrXX	130883	136797		8	0	0	0	2	6
ENSGACG00000014253	ChrIII	3157734	3197547	carmil3	8	0	0	0	3	5

ENSGACG00000015837	ChrXIV	1372274	1375579		8	0	0	0	8	0
ENSGACG00000017653	ChrIX	8567630	8572176		8	6	0	6	1	1
ENSGACG00000003164	ChrV	2512372	2514082		7	0	0	0	7	0
ENSGACG00000016341	ChrIV	229120	240408	si:dkeyp-110e4.11	7	0	0	0	3	4
ENSGACG00000006040	ChrXV	2368176	2420656	RYR3 (1 of many)	6	0	0	0	1	5
ENSGACG00000007557	ChrXX	8805057	8875965		6	0	0	0	3	3
ENSGACG00000008805	ChrX	13142212	13171897	ST3GAL1 (1 of many)	6	0	0	0	6	0
ENSGACG00000013273	ChrXIII	16131688	16140819	arhgap25	6	0	0	0	6	0
ENSGACG00000017845	ChrIX	8726948	8730779	lgi2b	6	0	0	0	0	6
ENSGACG00000018576	ChrIX	13131695	13177741	tusc3	6	0	0	0	6	0
ENSGACG00000001150	scaffold_237	44323	48391		5	0	0	0	2	3
ENSGACG00000007077	ChrXII	9114925	9116176	cort	5	0	0	0	0	5
ENSGACG00000009737	ChrV	11530314	11534646	WIPI1 (1 of many)	5	0	0	0	5	0
ENSGACG00000010295	ChrXI	8105454	8118846		5	0	0	0	3	2
ENSGACG00000011405	ChrI	13139185	13152967	stim1a	5	0	0	0	4	1
ENSGACG00000014351	ChrXI	14886837	14931008		5	0	0	0	4	1
ENSGACG00000017710	ChrIII	15790255	15791829		5	0	0	0	5	0
ENSGACG00000018311	ChrIV	12800220	12810446	eda	5	3	3	0	0	2
ENSGACG00000001679	ChrXXI	614128	678875	mpp7a	4	0	0	0	4	0
ENSGACG00000003086	ChrV	2254186	2259462	trim8b	4	0	0	0	4	0
ENSGACG00000004201	ChrXXI	9588132	9594424	LPIN2 (1 of many)	4	0	0	0	4	0
ENSGACG00000004882	ChrXXI	10624323	10627058		4	2	2	0	1	1
ENSGACG00000004909	ChrXIII	2375287	2400699	si:dkey-84j12,1	4	0	0	0	4	0
ENSGACG00000005913	ChrX	8577242	8596962	elp2	4	0	0	0	3	1
ENSGACG00000006038	ChrXVII	4531155	4544248	znfx1	4	0	0	0	3	1
ENSGACG00000006851	ChrV	9125837	9132564		4	4	0	4	0	0
ENSGACG00000008558	ChrVIII	9695227	9702494	si:ch211-43f4.1	4	0	0	0	3	1
ENSGACG00000010182	ChrXIII	9952936	9956281	zgc:110353	4	0	0	0	4	0

ENSGACG00000010247	ChrVI	12459328	12462277	si:ch211-248a14.8	4	0	0	0	4	0
ENSGACG00000013411	ChrIII	1381685	1420334		4	0	0	0	4	0
ENSGACG00000013470	ChrXX	16251960	16257880	CTSS	4	0	0	0	4	0
ENSGACG00000017082	ChrII	19781208	19783294	csrp3	4	0	0	0	3	1
ENSGACG00000017422	ChrXIV	7780909	7787080	ambp	4	0	0	0	4	0
ENSGACG00000018444	ChrIV	14030866	14039363	adam19b	4	0	0	0	4	0
ENSGACG00000018465	ChrIX	12301295	12303829	NOCT (1 of many)	4	0	0	0	1	3
ENSGACG00000019155	ChrIV	22038773	22045261	mcm10	4	0	0	0	4	0
ENSGACG00000020636	ChrVII	21643034	21651293	cfb	4	2	2	0	1	1
ENSGACG00000001586	scaffold_27	4643827	4660572	wnk2	3	0	0	0	1	2
ENSGACG00000003140	ChrX	3938771	3955496	TGFBR2	3	0	0	0	2	1
ENSGACG00000003570	ChrXVI	7947036	8001612	myo16	3	0	0	0	3	0
ENSGACG00000004301	ChrVIII	2431974	2441330	brdt	3	0	0	0	3	0
ENSGACG00000004534	ChrX	6955694	6967472	ef01a	3	0	0	0	3	0
ENSGACG00000004850	ChrXVII	2873238	2885900	slc2a1b	3	0	0	0	3	0
ENSGACG00000006314	ChrXVI	12033164	12044262		3	2	2	0	0	1
ENSGACG00000007093	ChrI	4607682	4609548	tiparp	3	0	0	0	3	0
ENSGACG00000007383	ChrXII	9777368	9778303		3	0	0	0	3	0
ENSGACG00000007722	ChrXVIII	6627166	6639844	rab15	3	0	0	0	0	3
ENSGACG00000007746	ChrXV	4848926	4850768	tmed8	3	0	0	0	0	3
ENSGACG00000009731	ChrX	14983618	14987412	mhc1zea	3	0	0	0	3	0
ENSGACG00000010537	ChrXVII	10768218	10769153	inka1b	3	0	0	0	3	0
ENSGACG00000010563	scaffold_210	56747	62418	si:dkey-190l8.2	3	1	0	1	1	1
ENSGACG00000010565	ChrXVIII	10469304	10475665		3	0	0	0	2	1
ENSGACG00000011263	ChrXIII	12814874	12817907		3	0	0	0	2	1
ENSGACG00000012318	ChrXVIII	13638248	13642046	clic5b	3	3	3	0	0	0
ENSGACG00000013121	ChrXVIII	15160892	15161878		3	0	0	0	2	1
ENSGACG00000013254	ChrXVIII	15436938	15444996	tpd52l1	3	0	0	0	3	0
ENSGACG00000014260	ChrXIII	18434585	18440058	hspb8	3	0	0	0	3	0

ENSGACG00000014962	scaffold_74	309652	319392		3	0	0	0	3	0
ENSGACG00000016010	ChrIII	9180647	9183385		3	0	0	0	0	3
ENSGACG00000016619	ChrXIV	5212300	5279453	rgs3b	3	0	0	0	3	0
ENSGACG00000017087	ChrII	19884513	19943913	0v2a	3	0	0	0	3	0
ENSGACG00000018250	ChrIV	12158019	12160272	irg1l (1 of many)	3	0	0	0	3	0
ENSGACG00000018330	ChrIX	10996164	11001903	KCNIP2	3	0	0	0	0	3
ENSGACG00000019294	ChrVII	3216567	3220967	CCKAR	3	0	0	0	0	3
ENSGACG00000019897	ChrIV	31023275	31026361	ada2b	3	0	0	0	1	2
ENSGACG00000020654	ChrVII	21884931	21941223	nrg2a	3	0	0	0	2	1
ENSGACG00000022752	scaffold_90	72943	73071	RF00263	3	0	0	0	3	0
ENSGACG00000000086	scaffold_99	339701	347704	brpf1	2	0	0	0	2	0
ENSGACG00000000307	scaffold_27	48405	52111		2	0	0	0	2	0
ENSGACG00000000972	scaffold_37	1733384	1743429	lancl2	2	0	0	0	2	0
ENSGACG00000000969	scaffold_47	1687223	1699812	zcchc4	2	0	0	0	0	2
ENSGACG00000001304	scaffold_156	64555	69127	desi2	2	0	0	0	1	1
ENSGACG00000001438	ChrXVI	125393	139423	rrp1	2	0	0	0	2	0
ENSGACG00000002344	ChrX	1751605	1763088	nedd9	2	0	0	0	2	0
ENSGACG00000002370	ChrV	793362	803990	SPOCK2	2	0	0	0	2	0
ENSGACG00000003413	ChrXVII	569558	572420		2	0	0	0	1	1
ENSGACG00000003522	ChrXVII	805182	813396	ywhaba	2	0	0	0	2	0
ENSGACG00000003564	ChrXVI	7915270	7918611	irs2b	2	0	0	0	2	0
ENSGACG00000003851	ChrXVI	8803555	8808879	gpr143	2	2	1	1	0	0
ENSGACG00000003865	ChrXXI	8925778	8930097	insig1	2	0	0	0	2	0
ENSGACG00000004450	ChrV	5466348	5479796	ubald1b	2	0	0	0	2	0
ENSGACG00000004657	ChrXVIII	1241770	1243679		2	0	0	0	0	2
ENSGACG00000004780	ChrXII	4743053	4896679	plx01a	2	0	0	0	1	1
ENSGACG00000005146	ChrXXI	11006245	11014630	gad2	2	0	0	0	2	0
ENSGACG00000005222	ChrXVII	3437445	3438929	kc02a	2	1	1	0	0	1
ENSGACG00000005316	ChrXII	5810740	5816656	fam131c	2	0	0	0	1	1

ENSGACG00000005369	ChrXV	1628452	1643542	galnt16	2	0	0	0	2	0
ENSGACG00000005488	ChrXXI	11359656	11367398	phex	2	2	2	0	0	0
ENSGACG00000005778	ChrXVI	11091546	11093393	dap1b	2	1	1	0	1	0
ENSGACG00000006075	ChrXVII	4589002	4596182	cbfa2t2	2	0	0	0	2	0
ENSGACG00000006148	ChrI	2319386	2323033		2	0	0	0	1	1
ENSGACG00000006187	ChrXVII	4907492	4911313	timm17a	2	0	0	0	2	0
ENSGACG00000006193	ChrVI	7611306	7689548	DST	2	0	0	0	2	0
ENSGACG00000006321	ChrI	3060604	3065797	p2rx8	2	0	0	0	2	0
ENSGACG00000006570	ChrXVIII	4669062	4680200	amd1	2	0	0	0	2	0
ENSGACG00000007232	ChrXV	4137556	4147913	lck	2	0	0	0	2	0
ENSGACG00000007241	ChrXV	4150772	4153424	fam167b	2	0	0	0	0	2
ENSGACG00000007668	ChrXX	8962606	8987449	aplp1	2	0	0	0	2	0
ENSGACG00000007915	ChrXV	5068567	5089769	tmem260	2	1	0	1	0	1
ENSGACG00000008836	ChrXV	6070712	6073477	klf11b	2	0	0	0	2	0
ENSGACG00000008991	ChrXII	11144529	11145968	sp5l	2	0	0	0	2	0
ENSGACG00000009263	ChrXII	11345449	11369829	fmnl3	2	0	0	0	2	0
ENSGACG00000009558	ChrXI	6851020	6922054	asic2	2	2	2	0	0	0
ENSGACG00000009925	ChrXVIII	9577490	9600773	lpgat1	2	0	0	0	2	0
ENSGACG00000010040	ChrVIII	12761712	12774504	ccdc50	2	0	0	0	0	2
ENSGACG00000010381	ChrX	15527868	15528362		2	0	0	0	0	2
ENSGACG00000010475	ChrVIII	13174745	13231603	insrb	2	0	0	0	2	0
ENSGACG00000010791	ChrXVIII	10774342	10785642	fndc4a	2	1	1	0	1	0
ENSGACG00000011302	ChrXII	15080122	15082735	mul1b	2	0	0	0	2	0
ENSGACG00000011602	ChrXVII	12652011	12662065	LAMB3	2	0	0	0	2	0
ENSGACG00000011732	ChrXVIII	12338480	12366063	myo6a	2	0	0	0	2	0
ENSGACG00000011811	ChrXVIII	12406545	12408354	COX7A2	2	0	0	0	2	0
ENSGACG00000012139	ChrI	14774869	14796093	yap1	2	0	0	0	2	0
ENSGACG00000012773	ChrXV	13276935	13293594	pdss2	2	1	1	0	0	1
ENSGACG00000013429	ChrXI	13025988	13026614		2	1	0	1	1	0

ENSGACG00000013428	ChrVIII	17395611	17400555	trmt13	2	0	0	0	2	0
ENSGACG00000013595	ChrXIII	16645839	16651528	vamp8	2	0	0	0	2	0
ENSGACG00000013744	ChrVIII	17762827	17768426		2	0	0	0	2	0
ENSGACG00000013743	ChrIII	1852860	1900173	tnr	2	0	0	0	2	0
ENSGACG00000014053	ChrI	20793942	20794313	nhlh2	2	0	0	0	2	0
ENSGACG00000014454	ChrXI	15389422	15396323	elac2	2	0	0	0	1	1
ENSGACG00000014902	ChrII	5427953	5521857	itfg1	2	0	0	0	1	1
ENSGACG00000016477	ChrXIV	3999374	4006176	agxt2 (1 of many)	2	0	0	0	2	0
ENSGACG00000016694	ChrII	17411270	17445836	chd9	2	0	0	0	1	1
ENSGACG00000017537	ChrXIV	8099548	8156144	bcr	2	0	0	0	2	0
ENSGACG00000018722	scaffold_208	83156	90702	iffo1a	2	0	0	0	2	0
ENSGACG00000019481	ChrIV	25424476	25433284	krr1	2	1	1	0	1	0
ENSGACG00000020034	ChrIV	31999298	32000748	tnnt2c	2	0	0	0	1	1
ENSGACG00000020040	ChrIV	32012155	32084033	btbd11a	2	0	0	0	2	0
ENSGACG00000020073	ChrIV	32350582	32355303	dclre1c	2	0	0	0	2	0
ENSGACG00000020706	ChrVII	22666912	22672421		2	0	0	0	1	1
ENSGACG00000020808	ChrVII	25496687	25519233	grk6	2	0	0	0	2	0
ENSGACG00000022198	ChrII	15100637	15100747		2	0	0	0	2	0
ENSGACG00000000048	scaffold_757	6140	6811		1	0	0	0	1	0
ENSGACG00000000162	scaffold_216	55071	61253	ACTR2	1	0	0	0	0	1
ENSGACG00000000381	scaffold_131	232303	234144	rbm46	1	1	1	0	0	0
ENSGACG00000000429	scaffold_80	140050	141102		1	0	0	0	1	0
ENSGACG00000000434	scaffold_80	184463	204053		1	0	0	0	1	0
ENSGACG00000000504	scaffold_139	130345	136698	DRC7	1	0	0	0	1	0
ENSGACG00000000525	scaffold_397	1558	15029		1	0	0	0	1	0
ENSGACG00000000634	scaffold_90	226420	229275	cradd	1	1	0	1	0	0
ENSGACG00000000795	scaffold_141	12636	18456	fgd1	1	0	0	0	0	1
ENSGACG00000000935	scaffold_1182	4446	4961		1	1	0	1	0	0
ENSGACG00000000950	scaffold_157	146047	149073	si:dkey-201i6.2	1	0	0	0	1	0

ENSGACG00000001006	scaffold_37	2177494	2216214	arhgap12b	1	0	0	0	1	0
ENSGACG00000001021	scaffold_37	2247366	2268896	si:ch211-276f18.2	1	0	0	0	1	0
ENSGACG00000001050	scaffold_37	2338551	2342806	aoc1	1	0	0	0	1	0
ENSGACG00000001087	scaffold_150	77433	80873		1	0	0	0	1	0
ENSGACG00000001219	scaffold_27	3491387	3517587	skib	1	0	0	0	1	0
ENSGACG00000001233	scaffold_181	61484	71960	adcy5	1	0	0	0	1	0
ENSGACG00000001282	scaffold_249	6147	18792	EHD4	1	0	0	0	1	0
ENSGACG00000001284	scaffold_130	39785	63580	sema4ab	1	0	0	0	1	0
ENSGACG00000001458	scaffold_273	15193	18488		1	0	0	0	1	0
ENSGACG00000001467	scaffold_273	23860	30175	sec24a	1	1	1	0	0	0
ENSGACG00000001644	scaffold_27	4804545	4808600	tmcc1b	1	1	1	0	0	0
ENSGACG00000001842	scaffold_600	385	1591		1	0	0	0	0	1
ENSGACG00000001883	ChrXVI	2628609	2645144	klf12a	1	0	0	0	1	0
ENSGACG00000001886	ChrXVI	2686951	2764612	dachd	1	0	0	0	1	0
ENSGACG00000001897	scaffold_326	46372	48199	mrps24	1	1	0	1	0	0
ENSGACG00000001979	ChrX	1148457	1148636		1	0	0	0	1	0
ENSGACG00000001980	scaffold_149	22071	23010		1	0	0	0	0	1
ENSGACG00000002013	scaffold_56	24216	25922		1	0	0	0	1	0
ENSGACG00000002128	ChrXXI	2737163	2755917	cngb3.1	1	0	0	0	1	0
ENSGACG00000002216	ChrXVI	4428375	4457157	ADARB1	1	0	0	0	1	0
ENSGACG00000002270	ChrX	1627559	1629451	tekt1	1	0	0	0	0	1
ENSGACG00000002326	ChrXXI	3947748	3952816	slc35b3	1	0	0	0	1	0
ENSGACG00000002374	ChrXXI	4343544	4350834	bco1	1	0	0	0	0	1
ENSGACG00000002418	ChrXXI	4917563	4971561	zfhx4	1	0	0	0	1	0
ENSGACG00000002439	ChrVI	624617	631220	degs1	1	0	0	0	1	0
ENSGACG00000002469	ChrXXI	5440575	5443026		1	0	0	0	0	1
ENSGACG00000002501	ChrXVI	5863099	5885871	slc4a3	1	0	0	0	1	0
ENSGACG00000002545	ChrX	2269766	2289402	nfatc1	1	0	0	0	1	0
ENSGACG00000002663	ChrV	1626313	1664933	ptprea	1	0	0	0	1	0

ENSGACG00000002670	ChrXXI	6065576	6128487	sema5a	1	0	0	0	0	1
ENSGACG00000002757	ChrXXI	6579026	6642145	stau2	1	0	0	0	0	1
ENSGACG00000002788	ChrXXI	6671169	6674187	SBSPON	1	0	0	0	1	0
ENSGACG00000002861	ChrV	1990736	1995983		1	0	0	0	0	1
ENSGACG00000002882	ChrXVI	7062666	7077323	ATP11A (1 of many)	1	0	0	0	1	0
ENSGACG00000002912	ChrXVI	7222380	7225324	txndc9	1	0	0	0	1	0
ENSGACG00000002982	ChrXII	1118299	1128392	TMCC1 (1 of many)	1	0	0	0	0	1
ENSGACG00000003032	ChrVI	2464760	2466700	fignl1	1	0	0	0	0	1
ENSGACG00000003031	ChrXVI	7391481	7394255	ecrg4a	1	0	0	0	1	0
ENSGACG00000003212	ChrVI	2759500	2773283	fam204a	1	0	0	0	1	0
ENSGACG00000003235	ChrXX	629982	640598	tmem145	1	0	0	0	1	0
ENSGACG00000003244	ChrX	4431116	4431815		1	1	0	1	0	0
ENSGACG00000003270	ChrV	2667010	2669113	si:ch73-127m5.2	1	0	0	0	1	0
ENSGACG00000003327	ChrX	4521465	4540223	ash1l	1	0	0	0	1	0
ENSGACG00000003323	ChrXII	1813168	1898170		1	0	0	0	1	0
ENSGACG00000003383	ChrVIII	1540683	1540880		1	0	0	0	1	0
ENSGACG00000003395	ChrXVI	7711922	7714172	asb11	1	0	0	0	1	0
ENSGACG00000003425	ChrXII	2053482	2071894	mitfb	1	0	0	0	1	0
ENSGACG00000003495	ChrXVII	699787	719603	GRIP2	1	0	0	0	1	0
ENSGACG00000003515	ChrXXI	8073271	8088883		1	0	0	0	0	1
ENSGACG00000003552	ChrX	5093819	5117753	pard6gb	1	0	0	0	1	0
ENSGACG00000003582	ChrXVI	8023067	8024372	tnfsf13b	1	0	0	0	1	0
ENSGACG00000003600	ChrXXI	8136415	8143194		1	1	1	0	0	0
ENSGACG00000003640	ChrXVII	1072273	1073132		1	0	0	0	1	0
ENSGACG00000003722	ChrV	4090150	4096264	meox1	1	0	0	0	1	0
ENSGACG00000003781	ChrXX	1859763	1890241		1	0	0	0	1	0
ENSGACG00000003807	ChrV	4374700	4425195	si:ch211-94l19.4	1	0	0	0	1	0
ENSGACG00000003932	ChrX	5372498	5374253		1	0	0	0	1	0

ENSGACG00000003937	ChrX	5419651	5422992	tshz1	1	1	1	0	0	0
ENSGACG00000003958	ChrVI	4091981	4093294	sertad2b	1	0	0	0	1	0
ENSGACG00000003974	ChrVI	4172491	4201696	b3gat2	1	0	0	0	1	0
ENSGACG00000004014	ChrXVI	8929582	8950765	tlr7	1	0	0	0	0	1
ENSGACG00000004100	ChrXVIII	313994	349188	e0h	1	0	0	0	0	1
ENSGACG00000004101	ChrXVI	9224446	9228563	dct	1	0	0	0	0	1
ENSGACG00000004118	ChrVI	4433273	4465659		1	0	0	0	0	1
ENSGACG00000004149	ChrVI	4667811	4669850	ntpcr	1	0	0	0	1	0
ENSGACG00000004339	ChrVIII	2537139	2541472		1	1	1	0	0	0
ENSGACG00000004294	ChrXX	2209827	2216716	sacm1lb	1	1	1	0	0	0
ENSGACG00000004352	ChrXVII	1895568	1903148		1	1	1	0	0	0
ENSGACG00000004352	ChrXVII	1895568	1903148		1	0	0	0	1	0
ENSGACG00000004383	ChrVIII	2891959	2898703	lmo4b	1	0	0	0	1	0
ENSGACG00000004402	ChrV	5177690	5178772	ccr10	1	0	0	0	1	0
ENSGACG00000004412	ChrXVII	1957372	2014039	arhgef10la	1	0	0	0	1	0
ENSGACG00000004431	ChrXII	3958137	3978532	ntsr1	1	1	0	1	0	0
ENSGACG00000004474	ChrV	5578449	5580362		1	0	0	0	1	0
ENSGACG00000004508	ChrXVIII	1155689	1171739	emilin1a	1	0	0	0	1	0
ENSGACG00000004559	ChrV	5619682	5624540	tmem184ba	1	0	0	0	1	0
ENSGACG00000004636	ChrI	372834	374001		1	1	1	0	0	0
ENSGACG00000004738	ChrXVII	2595419	2644876		1	0	0	0	1	0
ENSGACG00000004770	ChrXVI	10045160	10049909	SP3	1	0	0	0	1	0
ENSGACG00000004870	ChrXXI	10440079	10446151	dsela	1	1	1	0	0	0
ENSGACG00000004873	ChrXXI	10500632	10511464	CDH19	1	1	1	0	0	0
ENSGACG00000004924	ChrXII	5500786	5508872	brpf3b	1	0	0	0	1	0
ENSGACG00000004937	ChrV	5987158	5991150	zdhhc16b	1	0	0	0	1	0
ENSGACG00000004954	ChrXVII	3053327	3129303	grm4	1	0	0	0	1	0
ENSGACG00000005016	ChrVIII	3619176	3623161		1	0	0	0	1	0
ENSGACG00000005087	ChrXXI	10930916	10935699	mastl	1	0	0	0	0	1

ENSGACG00000005081	ChrXV	1425353	1432246		1	0	0	0	0	1
ENSGACG00000005129	ChrVIII	3952798	3985032	si:ch211-242b18.1	1	0	0	0	0	1
ENSGACG00000005157	ChrXVI	10427406	10429718	ccdc173	1	0	0	0	0	1
ENSGACG00000005173	ChrI	931955	937054	hmbsa	1	0	0	0	0	1
ENSGACG00000005197	ChrX	7842276	7843919	ggcta	1	0	0	0	0	1
ENSGACG00000005264	ChrXVI	10507462	10514195	rdh1	1	0	0	0	1	0
ENSGACG00000005270	ChrXI	1017948	1036084		1	0	0	0	1	0
ENSGACG00000005280	ChrXVIII	2211718	2416099	nrxn3b	1	0	0	0	1	0
ENSGACG00000005319	ChrXIII	3168194	3174770		1	0	0	0	1	0
ENSGACG00000005284	ChrXVII	3503858	3508516	slc5a8l	1	0	0	0	1	0
ENSGACG00000005332	ChrVIII	4566579	4571023	atpaf1	1	0	0	0	1	0
ENSGACG00000005353	ChrVIII	4667337	4693951		1	0	0	0	1	0
ENSGACG00000005493	ChrXV	1689391	1695853	metap1	1	1	1	0	0	0
ENSGACG00000005584	ChrI	1585298	1585992		1	0	0	0	1	0
ENSGACG00000005603	ChrXX	4857164	4860534	has2	1	0	0	0	1	0
ENSGACG00000005647	ChrXV	1810555	1848494	sntg2	1	0	0	0	1	0
ENSGACG00000005688	ChrVIII	5057122	5059646		1	0	0	0	1	0
ENSGACG00000005678	ChrXVII	3951822	3964914	cers5	1	0	0	0	0	1
ENSGACG00000005740	ChrV	7636119	7639426		1	0	0	0	0	1
ENSGACG00000005765	ChrXVII	3986584	4098651	si:dkey-57c15.1	1	0	0	0	1	0
ENSGACG00000005753	ChrXII	6969334	7035500	celsr3	1	0	0	0	0	1
ENSGACG00000005814	ChrXV	2022468	2079686	LTBP2	1	0	0	0	1	0
ENSGACG00000005851	ChrXVI	11375726	11391780	kcnj3a	1	0	0	0	1	0
ENSGACG00000005869	ChrI	2075764	2085083	numbl	1	1	1	0	0	0
ENSGACG00000005912	ChrXVII	4162499	4184565	eya2	1	0	0	0	1	0
ENSGACG00000005959	ChrXIII	4441375	4446248	CKMT2	1	0	0	0	0	1
ENSGACG00000006022	ChrI	2206772	2213401	bace1	1	0	0	0	1	0
ENSGACG00000006044	ChrVIII	6015397	6017317		1	0	0	0	1	0
ENSGACG00000006136	ChrI	2281697	2283639		1	0	0	0	1	0

ENSGACG00000006168	ChrXVIII	3331871	3333721	col10a1b	1	1	1	0	0	0
ENSGACG00000006183	ChrXV	2631748	2632214	gchfr	1	0	0	0	1	0
ENSGACG00000006180	ChrVIII	6394455	6397463		1	1	1	0	0	0
ENSGACG00000006245	ChrXVIII	3473616	3494661	bach2b	1	0	0	0	0	1
ENSGACG00000006309	ChrXV	2723196	2735169	cep170b	1	0	0	0	1	0
ENSGACG00000006345	ChrXX	6003650	6006502	eomesb	1	0	0	0	1	0
ENSGACG00000006380	ChrXIII	5034648	5038273		1	0	0	0	0	1
ENSGACG00000006398	ChrXV	2806798	2812667	pak6a	1	0	0	0	1	0
ENSGACG00000006421	ChrX	9120079	9120495	ferd3l	1	0	0	0	1	0
ENSGACG00000006443	ChrXII	7864313	7874314	ppih	1	0	0	0	1	0
ENSGACG00000006458	ChrXVII	5292055	5300667	gli1	1	0	0	0	1	0
ENSGACG00000006465	ChrXVI	12261048	12264385	pofut2	1	0	0	0	0	1
ENSGACG00000006528	ChrXVI	12761910	12797556	cps1	1	1	1	0	0	0
ENSGACG00000006533	ChrXV	2891400	2894701		1	0	0	0	1	0
ENSGACG00000006537	ChrVI	7898542	7919293	mcph1	1	0	0	0	1	0
ENSGACG00000006563	ChrV	8413714	8419499	dclre1a	1	0	0	0	1	0
ENSGACG00000006591	ChrI	3486252	3490154		1	0	0	0	1	0
ENSGACG00000006594	ChrXVI	12865337	12867816		1	0	0	0	0	1
ENSGACG00000006597	ChrXVI	12870903	12877502		1	0	0	0	1	0
ENSGACG00000006604	ChrVIII	6966044	6979032	WDR63	1	0	0	0	1	0
ENSGACG00000006606	ChrXI	3172985	3193999	cpt1cb	1	0	0	0	1	0
ENSGACG00000006693	ChrXV	2973149	2977521		1	0	0	0	1	0
ENSGACG00000006736	ChrXII	8388213	8475879	KAZN (1 of many)	1	0	0	0	0	1
ENSGACG00000006837	ChrV	9060530	9065297	nmt1b	1	0	0	0	1	0
ENSGACG00000006800	ChrXV	3469650	3516066	col12a1a	1	0	0	0	1	0
ENSGACG00000006884	ChrXV	3547262	3568975	tfb1m	1	0	0	0	1	0
ENSGACG00000006896	ChrXI	3652039	3673572	grb2b	1	1	1	0	0	0
ENSGACG00000006931	ChrXV	3674888	3699659		1	0	0	0	1	0
ENSGACG00000006980	ChrXV	3714812	3741586		1	1	1	0	0	0

ENSGACG00000007218	ChrXIII	6430504	6435884	plk2b	1	0	0	0	1	0
ENSGACG00000007276	ChrI	5465852	5488747	mecom	1	0	0	0	0	1
ENSGACG00000007280	ChrXV	4171868	4184504	clic4	1	1	1	0	0	0
ENSGACG00000007293	ChrX	10477197	10572975	adgrb2	1	0	0	0	1	0
ENSGACG00000007320	ChrI	5512892	5515273		1	0	0	0	1	0
ENSGACG00000007342	ChrXIII	6570975	6572197	atoh1a	1	0	0	0	1	0
ENSGACG00000007372	ChrX	10807926	10961030	csmd2	1	1	0	1	0	0
ENSGACG00000007372	ChrX	10807926	10961030	csmd2	1	0	0	0	1	0
ENSGACG00000007422	ChrXX	8591407	8593694	prss1 (1 of many)	1	0	0	0	0	1
ENSGACG00000007449	ChrXI	4378241	4381177		1	1	1	0	0	0
ENSGACG00000007492	ChrX	11267031	11270381	heyl	1	0	0	0	1	0
ENSGACG00000007529	ChrVI	8795607	8802574	CNNM1 (1 of many)	1	0	0	0	1	0
ENSGACG00000007503	ChrXVI	14554631	14687685	lrp1bb	1	0	0	0	1	0
ENSGACG00000007578	ChrXVI	14827472	14849263	myo1b	1	0	0	0	1	0
ENSGACG00000007610	ChrX	11394452	11398811	slc6a18	1	0	0	0	0	1
ENSGACG00000007653	ChrXIII	7397696	7405305	0a35	1	0	0	0	1	0
ENSGACG00000007695	ChrI	5944178	5955498	porb	1	0	0	0	0	1
ENSGACG00000007724	ChrXI	4748362	4752125	cbx7a	1	0	0	0	1	0
ENSGACG00000007745	ChrXIII	7495399	7509368	EGFLAM	1	1	1	0	0	0
ENSGACG00000007773	ChrX	11796739	11880195	ptprua	1	0	0	0	1	0
ENSGACG00000007826	ChrX	12126255	12133705	lin28a	1	0	0	0	1	0
ENSGACG00000007825	ChrI	6236909	6237643	a0pc15	1	0	0	0	1	0
ENSGACG00000007869	ChrXV	4989079	4992230	ap5m1	1	0	0	0	1	0
ENSGACG00000007893	ChrXX	9249276	9251026		1	1	0	1	0	0
ENSGACG00000007889	ChrXVI	15735794	15745166	mpp4a	1	1	1	0	0	0
ENSGACG00000008000	ChrXIII	8084061	8086494	rfk	1	0	0	0	1	0
ENSGACG00000008041	ChrVIII	9282717	9296560		1	0	0	0	0	1
ENSGACG00000008073	ChrV	9942067	9963447	kcnc3a	1	0	0	0	1	0

ENSGACG00000008081	ChrV	9974960	9977703		1	0	0	0	1	0
ENSGACG00000008079	ChrXII	10283054	10285128	dclre1b	1	0	0	0	1	0
ENSGACG00000008092	ChrX	12446407	12460395	eepd1	1	0	0	0	1	0
ENSGACG00000008121	ChrI	6554830	6569989	pdgfd	1	0	0	0	0	1
ENSGACG00000008082	ChrXII	10316175	10365141	cpne5a	1	0	0	0	0	1
ENSGACG00000008174	ChrX	12578667	12611207	TRIO	1	0	0	0	1	0
ENSGACG00000008194	ChrVIII	9448685	9451926	si:dkey-197i20.6	1	0	0	0	1	0
ENSGACG00000008187	ChrV	10034443	10040862		1	0	0	0	1	0
ENSGACG00000008213	ChrV	10078945	10079847		1	0	0	0	0	1
ENSGACG00000008282	ChrXV	5566137	5574768	FGFRL1 (1 of many)	1	0	0	0	1	0
ENSGACG00000008305	ChrXVI	16460421	16461727		1	0	0	0	1	0
ENSGACG00000008509	ChrXX	9883924	9885147	NHLRC1	1	0	0	0	0	1
ENSGACG00000008542	ChrXV	5811952	5817096	tpp1	1	0	0	0	1	0
ENSGACG00000008613	ChrXV	5827640	5855519	evla	1	0	0	0	1	0
ENSGACG00000008642	ChrXVI	17305099	17306341		1	0	0	0	1	0
ENSGACG00000008710	ChrXVI	17405163	17405973		1	0	0	0	1	0
ENSGACG00000008728	ChrV	10795397	10797367	znf668	1	0	0	0	0	1
ENSGACG00000008764	ChrVI	9973371	9977148	ppifa	1	0	0	0	0	1
ENSGACG00000008862	ChrX	13440280	13453314		1	0	0	0	0	1
ENSGACG00000008937	ChrXI	6189394	6191153	zgc:91968	1	0	0	0	0	1
ENSGACG00000009012	ChrX	13753836	13755884		1	0	0	0	1	0
ENSGACG00000009025	ChrV	10893482	10895023	zgc:112496	1	0	0	0	1	0
ENSGACG00000009033	ChrV	10926848	10927459	h1f0	1	0	0	0	0	1
ENSGACG00000009037	ChrXVI	17622563	17645255	satb2	1	0	0	0	0	1
ENSGACG00000009043	ChrVI	10538527	10559377	pcsk2	1	0	0	0	0	1
ENSGACG00000009105	ChrV	10961013	10969417	nlrc3	1	0	0	0	1	0
ENSGACG00000009086	ChrVIII	11312464	11328558	rubcn	1	0	0	0	1	0
ENSGACG00000009135	ChrVI	10670867	10727803	pcdh15a	1	0	0	0	0	1

ENSGACG00000009211	ChrXVIII	8793962	8796629	si:dkey-65b13.13	1	0	0	0	1	0
ENSGACG00000009292	ChrXVII	8863605	8869133	slc2a9l1 (1 of many)	1	0	0	0	1	0
ENSGACG00000009317	ChrXVII	8948722	8981993	adamts9	1	0	0	0	1	0
ENSGACG00000009482	ChrXV	7346370	7363708	rcan3	1	0	0	0	0	1
ENSGACG00000009483	ChrVIII	11917528	11996766	negr1	1	0	0	0	1	0
ENSGACG00000009569	ChrXII	11864095	11913150		1	0	0	0	1	0
ENSGACG00000009658	ChrVI	11660215	11671041	ZRANB1 (1 of many)	1	0	0	0	1	0
ENSGACG00000009650	ChrXV	7669664	7725769	qkia	1	1	1	0	0	0
ENSGACG00000009678	ChrXV	7921527	7922252		1	0	0	0	1	0
ENSGACG00000009836	ChrXII	12234412	12236332		1	0	0	0	1	0
ENSGACG00000009889	ChrX	15258511	15263294	psmb4	1	0	0	0	0	1
ENSGACG00000009945	ChrXVII	9984936	9992528	BMP7 (1 of many)	1	0	0	0	1	0
ENSGACG00000009991	ChrXVIII	9665614	9671172	tagapb	1	0	0	0	0	1
ENSGACG00000009996	ChrXVII	10262746	10278516	dido1	1	0	0	0	1	0
ENSGACG00000010079	ChrXVII	10406369	10422223	zgc:92107	1	0	0	0	1	0
ENSGACG00000010337	ChrI	10680875	10691269	v2ra18	1	0	0	0	1	0
ENSGACG00000010320	ChrXVII	10630815	10635514	gnl3l	1	0	0	0	1	0
ENSGACG00000010352	ChrV	12242543	12244965		1	0	0	0	1	0
ENSGACG00000010363	ChrVI	12867904	12868960	sf3b5	1	0	0	0	1	0
ENSGACG00000010489	ChrVIII	13247354	13252601	use1	1	0	0	0	1	0
ENSGACG00000010572	ChrXX	12085828	12089631	foxj2	1	0	0	0	1	0
ENSGACG00000010616	ChrVIII	13406915	13410357	loxl5a	1	0	0	0	1	0
ENSGACG00000010598	ChrXI	8347830	8352415	rsad1	1	1	0	1	0	0
ENSGACG00000010688	ChrXV	9729005	9736264	zgc:101744	1	0	0	0	1	0
ENSGACG00000010643	ChrXVII	10920133	10925973		1	1	1	0	0	0
ENSGACG00000010716	ChrXIII	11838527	11847542	gda	1	0	0	0	0	1
ENSGACG00000010874	ChrXVIII	10910812	10916238	ccm2	1	0	0	0	1	0
ENSGACG00000010882	ChrXVIII	10919991	10923357	fam167ab	1	0	0	0	1	0

ENSGACG00000010897	ChrXIII	12056151	12080196	bmp1a	1	0	0	0	1	0
ENSGACG00000010930	ChrXV	9957811	9977956	ppp2r3a	1	0	0	0	1	0
ENSGACG00000011145	ChrVIII	13948367	13950370	dmrta2	1	0	0	0	1	0
ENSGACG00000011152	ChrVIII	13997640	14027578	elavl4	1	1	1	0	0	0
ENSGACG00000011194	ChrXII	14916639	14926699	syt6a	1	1	0	1	0	0
ENSGACG00000011202	ChrVI	14449274	14452562		1	0	0	0	1	0
ENSGACG00000011285	ChrVI	14524837	14528348		1	0	0	0	1	0
ENSGACG00000011353	ChrXII	15159858	15171195	mical1	1	0	0	0	0	1
ENSGACG00000011386	ChrXV	11043345	11061560	dtnbb	1	0	0	0	1	0
ENSGACG00000011404	ChrXIII	12942202	12948571		1	0	0	0	1	0
ENSGACG00000011408	ChrXI	10141661	10156859	pdgfab	1	0	0	0	1	0
ENSGACG00000011418	ChrXVII	12394671	12396051	mrps33	1	1	1	0	0	0
ENSGACG00000011472	ChrVIII	14527394	14529615		1	1	0	1	0	0
ENSGACG00000011496	ChrI	13367609	13368755		1	0	0	0	1	0
ENSGACG00000011517	ChrXVIII	11751680	11752669	gpr6	1	1	1	0	0	0
ENSGACG00000011596	ChrI	13626289	13631703	spartb	1	0	0	0	1	0
ENSGACG00000011621	ChrXX	12578998	12584703		1	0	0	0	1	0
ENSGACG00000011657	ChrXX	12630689	12639183	cyp11c1	1	1	0	1	0	0
ENSGACG00000011694	ChrXI	10642924	10653747	axin2	1	0	0	0	1	0
ENSGACG00000011737	ChrXI	10793354	10795700	cbx8a	1	0	0	0	1	0
ENSGACG00000011809	ChrXVII	13822213	13835193	rybpa	1	0	0	0	1	0
ENSGACG00000011892	ChrXVII	14100397	14121542	frmd4ba	1	0	0	0	1	0
ENSGACG00000011900	ChrXVIII	12613115	12627157	PRKD3	1	0	0	0	1	0
ENSGACG00000011935	ChrXVIII	12659540	12662849	r0seh1	1	0	0	0	0	1
ENSGACG00000012038	ChrXX	13088418	13095119	ef03b	1	0	0	0	1	0
ENSGACG00000012043	ChrXX	13166502	13166753	si:dkey-246i14.3	1	0	0	0	0	1
ENSGACG00000012063	ChrXV	11951816	11991325	actn1	1	0	0	0	0	1
ENSGACG00000012160	ChrVI	16702488	16708055	abhd12	1	0	0	0	1	0
ENSGACG00000012162	ChrI	14817967	14822383	pgr	1	0	0	0	0	1

ENSGACG00000012118	ChrVIII	15437418	15442478	elovl8b (1 of many)	1	0	0	0	1	0
ENSGACG00000012320	ChrXI	11499498	11522052	tnrc6c1	1	0	0	0	1	0
ENSGACG00000012326	ChrVIII	15624530	15629529	abhd17aa	1	0	0	0	1	0
ENSGACG00000012401	ChrXVIII	13839871	13882401	klhl29	1	0	0	0	1	0
ENSGACG00000012413	ChrXX	13626490	13629746	si:dkey-199f5.8	1	0	0	0	1	0
ENSGACG00000012443	ChrXX	13677919	13684983	ago1	1	0	0	0	1	0
ENSGACG00000012473	ChrXX	13875776	13876264	hamp (1 of many)	1	0	0	0	1	0
ENSGACG00000012649	ChrVIII	15997577	15998999		1	0	0	0	1	0
ENSGACG00000012757	ChrXI	12294325	12298638	0CC1	1	0	0	0	1	0
ENSGACG00000012760	ChrVIII	16214608	16225148	si:dkey-110c1.10	1	0	0	0	1	0
ENSGACG00000012754	ChrXX	14531859	14563311	agmo	1	0	0	0	0	1
ENSGACG00000012786	ChrXX	14572594	14617822	dgkb	1	0	0	0	1	0
ENSGACG00000012863	ChrIII	320213	322935	CXorf38	1	0	0	0	1	0
ENSGACG00000012876	ChrVIII	16469522	16475075	rgmd	1	0	0	0	1	0
ENSGACG00000012938	ChrXVIII	14983710	14985734	chac1	1	0	0	0	1	0
ENSGACG00000012944	ChrIII	448107	459938		1	0	0	0	1	0
ENSGACG00000013008	ChrXX	15116693	15118431		1	0	0	0	1	0
ENSGACG00000013036	ChrVIII	16820744	16827630	cilp2	1	0	0	0	1	0
ENSGACG00000013069	ChrIII	565595	593939	adcy1b	1	0	0	0	1	0
ENSGACG00000013071	ChrVIII	16932446	16936340	tmem161a	1	0	0	0	1	0
ENSGACG00000013153	ChrXII	18049614	18054108		1	0	0	0	1	0
ENSGACG00000013156	ChrXV	15523603	15528254	commd8	1	0	0	0	1	0
ENSGACG00000013168	ChrIII	815615	833164	CDH2 (1 of many)	1	0	0	0	0	1
ENSGACG00000013215	ChrXIII	16093221	16101732	mxd1	1	1	1	0	0	0
ENSGACG00000013228	ChrI	17134277	17138229		1	1	0	1	0	0
ENSGACG00000013248	ChrXVIII	15399767	15421191	nkain2	1	1	1	0	0	0
ENSGACG00000013291	ChrXIII	16174644	16177810	znf703	1	0	0	0	1	0
ENSGACG00000013297	ChrVIII	17242910	17245367	rab11ba	1	0	0	0	0	1

ENSGACG00000013330	ChrXX	16121368	16124233	tspan13a	1	0	0	0	1	0
ENSGACG00000013346	ChrXI	12863133	12864817		1	0	0	0	1	0
ENSGACG00000013389	ChrXVIII	15662633	15665757	0ga	1	0	0	0	1	0
ENSGACG00000013413	ChrXVIII	15692426	15698913	si:ch211-57i17.1	1	1	0	1	0	0
ENSGACG00000013478	scaffold_196	78142	81595		1	0	0	0	1	0
ENSGACG00000013492	ChrIII	1536538	1543454		1	1	0	1	0	0
ENSGACG00000013537	ChrVIII	17456886	17459393	s1pr1	1	0	0	0	1	0
ENSGACG00000013691	ChrXX	17223600	17228134	rspo1	1	0	0	0	0	1
ENSGACG00000013737	ChrXX	17284737	17296628		1	0	0	0	1	0
ENSGACG00000013866	ChrXX	17717428	17731718	inpp5b	1	0	0	0	1	0
ENSGACG00000013905	ChrXIII	17465408	17466387		1	0	0	0	0	1
ENSGACG00000013933	ChrI	20491288	20509048	xirp2a	1	0	0	0	1	0
ENSGACG00000013943	ChrIII	2351398	2375649		1	0	0	0	1	0
ENSGACG00000013973	ChrXIII	17562120	17593351	notch1b	1	0	0	0	0	1
ENSGACG00000013977	ChrVIII	18160785	18167767	SCML2 (1 of many)	1	1	1	0	0	0
ENSGACG00000014007	ChrXIII	17704841	17747886	col27a1b	1	0	0	0	0	1
ENSGACG00000014040	ChrVIII	18275004	18275726		1	0	0	0	1	0
ENSGACG00000014173	ChrXIII	18210034	18254621	ksr2	1	0	0	0	1	0
ENSGACG00000014216	ChrI	21155272	21181371	farp1	1	0	0	0	0	1
ENSGACG00000014252	ChrXIII	18343004	18348481	suds3	1	1	1	0	0	0
ENSGACG00000014284	ChrXIII	18582295	18583087		1	0	0	0	1	0
ENSGACG00000014303	ChrIII	3571387	3812973	astn1	1	1	1	0	0	0
ENSGACG00000014314	scaffold_112	27482	36338	HTRA1	1	0	0	0	0	1
ENSGACG00000014334	ChrXIII	18869454	18873205	tmem175	1	0	0	0	1	0
ENSGACG00000014343	ChrVIII	18848370	18849326		1	0	0	0	1	0
ENSGACG00000014350	ChrXIII	18889876	18895161	ascc2	1	1	1	0	0	0
ENSGACG00000014353	ChrI	21861214	21861551		1	0	0	0	1	0
ENSGACG00000014471	ChrXIII	19003720	19035311	slc4a4a	1	0	0	0	1	0

ENSGACG00000014505	scaffold_112	343720	345162	aqp8a,2	1	0	0	0	0	1
ENSGACG00000014620	scaffold_76	287090	304975	pitx3	1	0	0	0	1	0
ENSGACG00000014736	ChrXIII	19874116	19881489		1	0	0	0	0	1
ENSGACG00000014814	ChrII	5076191	5081585		1	0	0	0	1	0
ENSGACG00000014832	ChrII	5125390	5127551	gnrhr2	1	0	0	0	1	0
ENSGACG00000014892	ChrIII	6063746	6077226		1	0	0	0	0	1
ENSGACG00000014899	scaffold_74	165635	168241	gpd1c	1	0	0	0	1	0
ENSGACG00000014963	ChrI	23565974	23566795		1	0	0	0	1	0
ENSGACG00000014987	ChrXI	16469732	16473523	tsen54	1	0	0	0	0	1
ENSGACG00000015056	ChrI	24829395	24935529		1	0	0	0	1	0
ENSGACG00000015086	scaffold_68	309394	354193	CDH4 (1 of many)	1	0	0	0	1	0
ENSGACG00000015165	ChrIII	7054385	7057639	PRKAB2	1	0	0	0	1	0
ENSGACG00000015177	ChrI	25571569	25595585	map4k4	1	0	0	0	1	0
ENSGACG00000015260	ChrII	6665264	6668296	pcdh9	1	0	0	0	1	0
ENSGACG00000015262	ChrII	7099598	7102887	PCDH20	1	0	0	0	1	0
ENSGACG00000015264	ChrII	7115287	7128545	tdrd3	1	0	0	0	1	0
ENSGACG00000015273	ChrIII	7416267	7419629	map2k2b	1	0	0	0	1	0
ENSGACG00000015281	ChrII	7632856	7635853	trmt10c	1	1	1	0	0	0
ENSGACG00000015302	ChrII	7675747	7702944	rasa3	1	0	0	0	1	0
ENSGACG00000015374	ChrII	7942950	8012640		1	0	0	0	1	0
ENSGACG00000015377	ChrIII	7710663	7721459		1	0	0	0	1	0
ENSGACG00000015381	ChrIII	7730160	7749741	epha4a	1	0	0	0	1	0
ENSGACG00000015490	ChrXIV	174415	177418	ufc1	1	0	0	0	1	0
ENSGACG00000015516	ChrI	27641497	27646007		1	0	0	0	0	1
ENSGACG00000015581	ChrI	27801691	27803793	sumo1	1	0	0	0	1	0
ENSGACG00000015617	ChrI	27906006	27925118	ahr2	1	0	0	0	1	0
ENSGACG00000015651	ChrXIV	608441	613228	mat2al	1	1	0	1	0	0
ENSGACG00000015736	ChrXIV	723869	725546	mzt2b	1	0	0	0	0	1
ENSGACG00000015775	ChrXIV	1031417	1038996	ARSB	1	1	0	1	0	0

ENSGACG00000015791	ChrII	11127315	11148839	PHLPP2	1	0	0	0	1	0
ENSGACG00000015831	ChrII	11422567	11478619	tcf12	1	0	0	0	1	0
ENSGACG00000015862	ChrXIV	1681568	1684183	npffr2b	1	1	1	0	0	0
ENSGACG00000015884	ChrXIV	1751442	1766482	erbin	1	0	0	0	1	0
ENSGACG00000015902	ChrXIV	1944466	1968159	fnbp1b	1	1	0	1	0	0
ENSGACG00000015915	ChrXIV	2174485	2181252	ttll11	1	0	0	0	1	0
ENSGACG00000015949	ChrXIV	2711343	2720198	usp20	1	0	0	0	0	1
ENSGACG00000015999	ChrIX	791090	795086		1	0	0	0	1	0
ENSGACG00000016065	ChrIII	9230992	9232131	b3gnt5b	1	0	0	0	1	0
ENSGACG00000016073	ChrIX	1334639	1357056		1	0	0	0	0	1
ENSGACG00000016089	ChrXIV	3138466	3139400	rpl28	1	0	0	0	0	1
ENSGACG00000016100	ChrIX	1429086	1441309	polq	1	0	0	0	1	0
ENSGACG00000016150	ChrII	13614114	13615977	psmc3	1	0	0	0	1	0
ENSGACG00000016142	ChrXIV	3217562	3221176	atp5fa1 (1 of many)	1	1	1	0	0	0
ENSGACG00000016163	ChrIII	9641348	9647872	gpbp1l1	1	0	0	0	1	0
ENSGACG00000016169	ChrXIV	3251935	3256350		1	0	0	0	0	1
ENSGACG00000016193	ChrIX	1782128	1785348	chr09	1	0	0	0	1	0
ENSGACG00000016222	ChrXIV	3365899	3371406	kcmf1	1	0	0	0	1	0
ENSGACG00000016347	ChrIX	3127102	3143261	p2rx3b	1	0	0	0	1	0
ENSGACG00000016360	ChrIV	472586	477561	pcdh1b	1	0	0	0	0	1
ENSGACG00000016426	ChrIV	1302365	1304893	fgfrl1a	1	1	1	0	0	0
ENSGACG00000016451	ChrII	15348070	15353934	CTSH	1	0	0	0	1	0
ENSGACG00000016473	ChrXIV	3993506	3998272	prlra	1	0	0	0	1	0
ENSGACG00000016521	ChrII	15654499	15662194	fam214a	1	0	0	0	0	1
ENSGACG00000016606	ChrIX	4603846	4604358		1	0	0	0	1	0
ENSGACG00000016616	ChrIX	4624437	4630228	mief2	1	0	0	0	1	0
ENSGACG00000016624	ChrIV	2464056	2509522	rapgef2	1	0	0	0	1	0
ENSGACG00000016708	ChrIX	4885702	4887717		1	0	0	0	1	0

ENSGACG00000016723	ChrIX	5098704	5108431	g012a	1	0	0	0	1	0
ENSGACG00000016746	ChrII	18045594	18048728	gldn	1	1	1	0	0	0
ENSGACG00000016846	ChrIX	5456262	5476760		1	1	1	0	0	0
ENSGACG00000016916	ChrXIV	6417172	6426532	sgsm1b	1	0	0	0	1	0
ENSGACG00000016918	ChrIII	12322870	12341345		1	1	1	0	0	0
ENSGACG00000016952	ChrII	19109323	19110312	rxfp3,3a2	1	0	0	0	0	1
ENSGACG00000016957	ChrII	19149179	19152927	calml4a	1	1	0	1	0	0
ENSGACG00000016959	ChrXIV	6533608	6537690	svopb	1	0	0	0	0	1
ENSGACG00000016992	ChrII	19281893	19298785	znf609b	1	0	0	0	1	0
ENSGACG00000017000	ChrII	19349048	19401226	myo9aa	1	0	0	0	1	0
ENSGACG00000017063	ChrII	19650101	19656297		1	0	0	0	1	0
ENSGACG00000017074	ChrIV	5485973	5494737	efemp2a	1	0	0	0	1	0
ENSGACG00000017077	ChrIV	5517696	5539310	mrpl11	1	0	0	0	1	0
ENSGACG00000017096	ChrII	20027174	20041230	slc6a5	1	0	0	0	1	0
ENSGACG00000017124	ChrIX	6068561	6084066		1	0	0	0	1	0
ENSGACG00000017160	ChrII	21080854	21084821		1	0	0	0	0	1
ENSGACG00000017209	ChrXIV	6969603	6972066	pcyox1	1	0	0	0	1	0
ENSGACG00000017255	ChrIX	7204599	7294154	inpp4b	1	0	0	0	1	0
ENSGACG00000017313	ChrII	21709930	21734771	MEGF11 (1 of many)	1	1	0	1	0	0
ENSGACG00000017319	ChrII	21769057	21771819	socs4	1	1	1	0	0	0
ENSGACG00000017384	ChrII	22035294	22037750	ndufs3	1	0	0	0	1	0
ENSGACG00000017389	ChrXIV	7665108	7673135	urm1	1	0	0	0	1	0
ENSGACG00000017407	ChrXIV	7749442	7751978	fam163b	1	0	0	0	1	0
ENSGACG00000017464	ChrII	22240099	22241218		1	0	0	0	1	0
ENSGACG00000017476	ChrII	22301954	22308632	cpeb1b	1	0	0	0	0	1
ENSGACG00000017506	ChrII	22373022	22375772	nqo1 (1 of many)	1	0	0	0	1	0
ENSGACG00000017504	ChrIV	7553451	7640413	enox2	1	0	0	0	1	0
ENSGACG00000017542	ChrII	22570349	22571189		1	0	0	0	1	0

ENSGACG00000017563	ChrIV	8075659	8076747		1	0	0	0	1	0
ENSGACG00000017568	ChrIII	14440090	14454692	PFKP (1 of many)	1	0	0	0	0	1
ENSGACG00000017613	ChrII	22859068	22883630	ppfia1	1	0	0	0	1	0
ENSGACG00000017647	ChrXIV	8597952	8633437	sfswap	1	0	0	0	0	1
ENSGACG00000017689	ChrIV	8520482	8545266	si:ch211-220e11.3	1	0	0	0	1	0
ENSGACG00000017699	ChrXIV	8996130	9029956	ank1b	1	0	0	0	0	1
ENSGACG00000017713	ChrIII	15794470	15804479		1	0	0	0	1	0
ENSGACG00000017773	ChrIV	9228627	9233379	rad9a	1	0	0	0	1	0
ENSGACG00000017893	ChrIII	16251876	16277818	cac01eb	1	0	0	0	1	0
ENSGACG00000017924	ChrXIV	10388550	10398180	ghrb	1	0	0	0	1	0
ENSGACG00000017934	ChrIV	10439115	10505618	si:dkey-201i24.6	1	1	1	0	0	0
ENSGACG00000017983	ChrIII	16717673	16723180	atg9b	1	1	1	0	0	0
ENSGACG00000017998	ChrIV	10730374	10732632	arsia	1	0	0	0	1	0
ENSGACG00000018028	ChrIX	9645774	9651002	spock3	1	1	1	0	0	0
ENSGACG00000018121	ChrXIV	11304434	11333562	grin1a	1	0	0	0	1	0
ENSGACG00000018145	ChrXIV	11471663	11476209	barhl1b	1	0	0	0	1	0
ENSGACG00000018172	ChrXIV	11597815	11602791	adra1ab	1	0	0	0	0	1
ENSGACG00000018179	ChrXIV	11850201	11893984	vav2	1	0	0	0	1	0
ENSGACG00000018233	ChrIX	10713754	10725152	pdcd11	1	1	1	0	0	0
ENSGACG00000018231	ChrIV	12013740	12034920	abcb7	1	0	0	0	1	0
ENSGACG00000018322	ChrIV	12941555	12943706		1	0	0	0	1	0
ENSGACG00000018323	ChrXIV	13873722	13900598	lmx1ba	1	0	0	0	0	1
ENSGACG00000018335	ChrXIV	13922425	13930656	mapkap1	1	0	0	0	1	0
ENSGACG00000018341	ChrIX	11059538	11064667	ogal	1	1	1	0	0	0
ENSGACG00000018360	ChrIX	11194695	11200674	lrpap1	1	0	0	0	1	0
ENSGACG00000018375	ChrXIV	14135402	14160309		1	1	0	1	0	0
ENSGACG00000018409	ChrIX	11666673	11682015	pcdh10a	1	0	0	0	1	0
ENSGACG00000018461	ChrIV	14219527	14248769	ablim3	1	0	0	0	1	0
ENSGACG00000018600	ChrVII	242336	245534		1	1	1	0	0	0

ENSGACG00000018633	ChrVII	365776	372621		1	0	0	0	1	0
ENSGACG00000018652	ChrVII	494053	497077		1	0	0	0	1	0
ENSGACG00000018663	ChrIV	16118651	16126776	tmem173	1	0	0	0	1	0
ENSGACG00000018759	ChrVII	835005	836654		1	0	0	0	0	1
ENSGACG00000018788	ChrVII	1108957	1111978	tmem185	1	1	0	1	0	0
ENSGACG00000018802	ChrVII	1133367	1137045		1	0	0	0	1	0
ENSGACG00000018836	ChrIV	18419217	18424359	sypl1	1	1	1	0	0	0
ENSGACG00000018841	ChrIV	18438511	18458268	gsap	1	0	0	0	1	0
ENSGACG00000018867	ChrVII	1452630	1459173	khnyn	1	0	0	0	0	1
ENSGACG00000018876	ChrVII	1481900	1484089	emc4	1	0	0	0	0	1
ENSGACG00000018929	ChrIV	19626362	19645224		1	0	0	0	1	0
ENSGACG00000018991	ChrVII	2259206	2267772	zgc:55262	1	0	0	0	0	1
ENSGACG00000019026	ChrIV	20833178	20835191	alg10	1	0	0	0	0	1
ENSGACG00000019034	ChrIV	20945694	20956175	abcd2	1	0	0	0	1	0
ENSGACG00000019043	ChrIX	15241216	15246931	pycr1b	1	1	1	0	0	0
ENSGACG00000019101	ChrVII	2678788	2682285	rgp1	1	0	0	0	1	0
ENSGACG00000019117	ChrVII	2701856	2708450	si:dkeyp-75h12.2	1	0	0	0	1	0
ENSGACG00000019125	ChrIV	21818906	21878888	SYN3	1	0	0	0	0	1
ENSGACG00000019138	ChrVII	2808940	2812270		1	0	0	0	1	0
ENSGACG00000019176	ChrIV	22147684	22153447	pfkfb3	1	0	0	0	1	0
ENSGACG00000019227	ChrIV	22899577	22913555	mkrn1	1	0	0	0	0	1
ENSGACG00000019253	ChrIV	23209606	23250489		1	1	1	0	0	0
ENSGACG00000019286	ChrVII	3160049	3162174	si:dkey-19b23.7	1	0	0	0	1	0
ENSGACG00000019353	ChrIX	16630442	16684711	MGAT5B (1 of many)	1	0	0	0	1	0
ENSGACG00000019362	ChrVII	4248635	4260779	neurl4	1	0	0	0	1	0
ENSGACG00000019440	ChrIV	24711763	24712875		1	0	0	0	1	0
ENSGACG00000019460	ChrIX	17631571	17632626	dand5	1	0	0	0	1	0
ENSGACG00000019472	ChrIV	25318371	25416178	0v3	1	0	0	0	1	0

ENSGACG00000019498	ChrVII	5325617	5432198	ADGRL3	1	0	0	0	0	1
ENSGACG00000019674	ChrIV	28658720	28716045	wnk1b	1	0	0	0	1	0
ENSGACG00000019679	ChrIV	28725325	28735256		1	0	0	0	1	0
ENSGACG00000019805	ChrIV	30481780	30482190	btg1	1	0	0	0	1	0
ENSGACG00000019945	ChrIV	31129826	31165134	ptn	1	0	0	0	1	0
ENSGACG00000019970	ChrIV	31495299	31507946	si:dkey-97m3.1	1	0	0	0	1	0
ENSGACG00000020022	ChrVII	9404415	9405080	zgc:113305	1	0	0	0	1	0
ENSGACG00000020033	ChrVII	10133090	10158972	si:cabz01078036.1	1	0	0	0	1	0
ENSGACG00000020045	ChrIV	32117996	32125903	parpbp	1	0	0	0	1	0
ENSGACG00000020050	ChrIV	32208749	32220861	nrip2	1	0	0	0	1	0
ENSGACG00000020051	ChrIV	32222974	32224772	klhl42	1	0	0	0	1	0
ENSGACG00000020174	ChrVII	12750327	12755673	capns1a	1	0	0	0	1	0
ENSGACG00000020311	ChrVII	16430416	16478058		1	0	0	0	0	1
ENSGACG00000020325	ChrVII	17024309	17031165		1	0	0	0	1	0
ENSGACG00000020369	ChrVII	17649875	17653814		1	0	0	0	1	0
ENSGACG00000020401	ChrVII	17997085	18001531	si:ch211-137i24.10	1	1	0	1	0	0
ENSGACG00000020426	ChrVII	18408108	18413324	taf6	1	0	0	0	0	1
ENSGACG00000020448	ChrVII	18690919	18691724		1	1	0	1	0	0
ENSGACG00000020556	ChrVII	20054776	20129917	nbeaa	1	0	0	0	1	0
ENSGACG00000020577	ChrVII	20565349	20569644	eva1c	1	0	0	0	1	0
ENSGACG00000020658	ChrVII	22028152	22042272	ppp3ca	1	0	0	0	1	0
ENSGACG00000020675	ChrVII	22206068	22209023		1	0	0	0	0	1
ENSGACG00000020726	ChrVII	23208162	23253092	ARHGAP26	1	0	0	0	1	0
ENSGACG00000020747	ChrVII	23962472	23988220	dpysl3	1	0	0	0	1	0
ENSGACG00000020803	ChrVII	25380828	25384895		1	0	0	0	1	0
ENSGACG00000020805	ChrVII	25418091	25465731	dbn1	1	1	1	0	0	0
ENSGACG00000020811	ChrVII	25541259	25547799		1	0	0	0	1	0
ENSGACG00000020824	ChrVII	26193351	26195752	cnot8	1	1	0	1	0	0

ENSGACG00000020842	ChrVII	26490578	26493742	rbm41	1	0	0	0	1	0
ENSGACG00000020848	ChrVII	26791004	26845216	doc2b	1	0	0	0	0	1
ENSGACG00000020849	ChrVII	26855161	26860699	gatsl2	1	0	0	0	1	0
ENSGACG00000021308	ChrIII	9959732	9959817		1	0	0	0	1	0
ENSGACG00000021339	ChrXIII	17724932	17725023		1	0	0	0	1	0
ENSGACG00000021543	ChrVIII	10666396	10666480		1	0	0	0	0	1
ENSGACG00000022289	ChrVII	19110984	19111069		1	0	0	0	1	0
ENSGACG00000022499	ChrX	9755442	9755535		1	0	0	0	1	0
ENSGACG00000022573	ChrI	4299459	4299517		1	0	0	0	0	1
ENSGACG00000022581	ChrX	9041304	9041363		1	1	1	0	0	0
ENSGACG00000022669	ChrVIII	10778399	10778538	RF00191	1	0	0	0	1	0
ENSGACG00000022764	ChrIX	5186427	5186513	RF00271	1	0	0	0	1	0

Table S2B. Differentially methylated genes between populations from KIE (20 PSU) and SYL (33 PSU). For genes associated with DMS, Ensembl gene ID and gene name as well as the position on the chromosome are listed. The numbers refer to the numbers of DMS in the population comparison (wild), these DMS were classified into 'inducible', 'inconclusive' and 'stable' sites according to their behavior in a two-generation salinity acclimation experiment with laboratory bred sticklebacks from Kiel (20 PSU) exposed to experimental salinity increase (33 PSU) (see Methods for details). Further, inducible sites were distinguished whether they matched methylation levels of the locally adapted population ('*expected*') or not ('*opposite*').

Ensembl gene ID	chromosome	start position	end position	gene name	wild	inducible	expected inducible	opposite inducible	stable	inconclusive
ENSGACG00000020323	ChrVII	17010160	17011176		23	0	0	0	22	1
ENSGACG00000013229	ChrXVIII	15327717	15352321		15	10	10	0	1	4
ENSGACG00000013359	ChrXI	12960883	12968110	sec14l1	15	0	0	0	12	3

ENSGACG00000019416	ChrVII	4451892	4453656	HMX1 orthologue	15	3	3	0	5	7
ENSGACG00000002948	ChrVIII	218240	221355	ddx10	14	0	0	0	6	8
ENSGACG00000016350	ChrXIV	3603545	3604923		14	1	0	1	7	6
ENSGACG00000006636	ChrXVIII	4780893	4786820	ZC3H12D	13	0	0	0	3	10
ENSGACG00000004667	ChrXII	4273498	4286193	tti1	12	0	0	0	12	0
ENSGACG00000015566	ChrII	9043062	9051779	casc4	10	0	0	0	10	0
ENSGACG00000004433	ChrXVII	2127457	2211376	igsf21a	9	9	9	0	0	0
ENSGACG00000006772	ChrXV	3284481	3316132	dlgap2a	9	0	0	0	7	2
ENSGACG00000008805	ChrX	13142212	13171897	ST3GAL1 (1 of many)	9	0	0	0	8	1
ENSGACG00000008919	ChrX	13502303	13521318	kcnk9	8	0	0	0	0	8
ENSGACG00000020706	ChrVII	22666912	22672421		8	0	0	0	8	0
ENSGACG00000004770	ChrXVI	10045160	10049909	SP3	7	0	0	0	5	2
ENSGACG00000014483	ChrXI	15438933	15454835	gas7a	7	0	0	0	7	0
ENSGACG00000015862	ChrXIV	1681568	1684183	npffr2b	7	0	0	0	7	0
ENSGACG00000016341	ChrIV	229120	240408	si:dkeyp-110e4.11	7	0	0	0	0	7
ENSGACG00000019897	ChrIV	31023275	31026361	ada2b	7	0	0	0	0	7
ENSGACG00000006040	ChrXV	2368176	2420656	RYR3 (1 of many)	6	3	0	3	0	3
ENSGACG00000012193	ChrXVIII	13344808	13352902		6	0	0	0	6	0
ENSGACG00000015057	scaffold_74	485843	502285	pkp4	6	0	0	0	6	0
ENSGACG00000017845	ChrIX	8726948	8730779	lgi2b	6	0	0	0	0	6
ENSGACG00000018110	ChrIX	10035146	10041439	krt222	6	0	0	0	6	0
ENSGACG00000018576	ChrIX	13131695	13177741	tusc3	6	0	0	0	0	6
ENSGACG00000001675	scaffold_120	284160	295979	LAMC2	5	0	0	0	2	3
ENSGACG00000002963	ChrXX	130883	136797		5	2	2	0	0	3
ENSGACG00000005061	ChrXVI	10329406	10372640	myo3b	5	0	0	0	0	5
ENSGACG00000009482	ChrXV	7346370	7363708	rcan3	5	0	0	0	3	2
ENSGACG00000010846	ChrVIII	13598498	13605960	lhx4	5	1	1	0	1	3
ENSGACG00000011118	ChrI	12219197	12219822		5	0	0	0	2	3

ENSGACG00000011657	ChrXX	12630689	12639183	cyp11c1	5	3	0	3	1	1
ENSGACG00000011995	ChrXV	11740267	11769069	trim9	5	0	0	0	2	3
ENSGACG00000020047	ChrIV	32137226	32150243	th2	5	0	0	0	5	0
ENSGACG00000001000	scaffold_108	236983	240230	hvcn1	4	0	0	0	4	0
ENSGACG00000002150	scaffold_56	991932	1000901	b4galt6	4	0	0	0	0	4
ENSGACG00000003831	ChrXVII	1268650	1270064		4	1	1	0	1	2
ENSGACG00000003932	ChrX	5372498	5374253		4	0	0	0	4	0
ENSGACG00000008558	ChrVIII	9695227	9702494	si:ch211-43f4.1	4	0	0	0	4	0
ENSGACG00000008986	ChrX	13667723	13673241	ube2ql1	4	0	0	0	3	1
ENSGACG00000011019	ChrXI	9080011	9104314	cyth1a	4	0	0	0	4	0
ENSGACG00000017534	ChrXIV	8089235	8092718		4	0	0	0	3	1
ENSGACG00000001150	scaffold_237	44323	48391		3	1	1	0	1	1
ENSGACG00000002270	ChrX	1627559	1629451	tekt1	3	0	0	0	1	2
ENSGACG00000002707	ChrXII	560982	576138	grip2a	3	0	0	0	3	0
ENSGACG00000002865	ChrXII	847025	855389	prickle2a	3	0	0	0	2	1
ENSGACG00000005778	ChrXVI	11091546	11093393	dap1b	3	0	0	0	3	0
ENSGACG00000007036	ChrXIII	6072435	6095546		3	0	0	0	3	0
ENSGACG00000009367	ChrV	11265977	11279015	baiap2l1a	3	0	0	0	2	1
ENSGACG00000010367	ChrXVIII	10300965	10314744	acss1	3	0	0	0	3	0
ENSGACG00000010998	ChrXVII	11632756	11638904	cldn19	3	0	0	0	0	3
ENSGACG00000012122	ChrXV	12090680	12101068	map3k9	3	0	0	0	3	0
ENSGACG00000014293	ChrVIII	18757229	18761036	tmem165	3	0	0	0	3	0
ENSGACG00000015715	ChrII	10395592	10421700	tead1b	3	0	0	0	3	0
ENSGACG00000016957	ChrII	19149179	19152927	calml4a	3	2	0	2	0	1
ENSGACG00000017210	ChrIII	13216739	13218719	zgc:113531	3	0	0	0	1	2
ENSGACG00000017236	ChrII	21381632	21396647		3	0	0	0	3	0
ENSGACG00000018330	ChrIX	10996164	11001903	KCNIP2	3	0	0	0	1	2
ENSGACG00000020808	ChrVII	25496687	25519233	grk6	3	0	0	0	3	0
ENSGACG00000021523	scaffold_80	401870	401961		3	0	0	0	3	0

ENSGACG00000000253	scaffold_114	74933	109669	itpr3	2	0	0	0	2	0
ENSGACG00000000935	scaffold_1182	4446	4961		2	1	0	1	1	0
ENSGACG00000000972	scaffold_37	1733384	1743429	lancl2	2	0	0	0	2	0
ENSGACG00000001842	scaffold_600	385	1591		2	0	0	0	0	2
ENSGACG00000002193	ChrV	298074	298954	DUSP13	2	0	0	0	2	0
ENSGACG00000002344	ChrX	1751605	1763088	nedd9	2	0	0	0	2	0
ENSGACG00000003215	ChrV	2545786	2550169	prph2a	2	0	0	0	2	0
ENSGACG00000003249	ChrXII	1592910	1631790	scube3	2	0	0	0	1	1
ENSGACG00000003868	ChrXV	24	4126		2	0	0	0	2	0
ENSGACG00000003922	ChrVI	4019342	4043269		2	0	0	0	2	0
ENSGACG00000004040	ChrXII	3116439	3118541		2	0	0	0	0	2
ENSGACG00000004212	ChrXIII	1654417	1666382	si:dkey-178e17.1	2	0	0	0	0	2
ENSGACG00000004339	ChrVIII	2537139	2541472		2	0	0	0	1	1
ENSGACG00000004526	ChrX	6917318	6925218	ef03a	2	0	0	0	2	0
ENSGACG00000004657	ChrXVIII	1241770	1243679		2	0	0	0	2	0
ENSGACG00000005316	ChrXII	5810740	5816656	fam131c	2	0	0	0	2	0
ENSGACG00000005631	ChrXI	1709563	1713578	hoxb3a	2	0	0	0	2	0
ENSGACG00000006038	ChrXVII	4531155	4544248	znfx1	2	0	0	0	1	1
ENSGACG00000006245	ChrXVIII	3473616	3494661	bach2b	2	0	0	0	0	2
ENSGACG00000006328	ChrXX	5969700	5976203		2	0	0	0	2	0
ENSGACG00000006387	ChrXX	6166089	6172879	entpd3	2	1	0	1	0	1
ENSGACG00000006456	ChrXVII	5282637	5335165		2	0	0	0	2	0
ENSGACG00000006638	ChrI	3984198	4032562	gramd1bb	2	0	0	0	2	0
ENSGACG00000007503	ChrXVI	14554631	14687685	lrp1bb	2	0	0	0	2	0
ENSGACG00000007773	ChrX	11796739	11880195	ptprua	2	1	0	1	1	0
ENSGACG00000008092	ChrX	12446407	12460395	eepd1	2	0	0	0	2	0
ENSGACG00000008187	ChrV	10034443	10040862		2	0	0	0	2	0
ENSGACG00000008829	ChrVI	10053208	10156710	kcnma1a	2	0	0	0	2	0
ENSGACG00000009156	ChrXVI	17703064	17704594		2	2	2	0	0	0

ENSGACG00000010158	ChrXII	12648568	12734883	ERC2 (1 of many)	2	2	2	0	0	0
ENSGACG00000010489	ChrVIII	13247354	13252601	use1	2	0	0	0	2	0
ENSGACG00000010661	ChrXI	8377436	8379317	znf750	2	0	0	0	2	0
ENSGACG00000011608	ChrXVIII	12075826	12137714	stxbp5a	2	0	0	0	1	1
ENSGACG00000011935	ChrXVIII	12659540	12662849	r0seh1	2	0	0	0	2	0
ENSGACG00000014285	ChrII	2430586	2434229	slc7a6os	2	0	0	0	2	0
ENSGACG00000014667	ChrXIII	19641934	19644791	coq5	2	0	0	0	2	0
ENSGACG00000016544	ChrIV	2203221	2209876	neil3	2	0	0	0	2	0
ENSGACG00000016953	ChrII	19113447	19146003		2	0	0	0	1	1
ENSGACG00000017000	ChrII	19349048	19401226	myo9aa	2	2	0	2	0	0
ENSGACG00000017077	ChrIV	5517696	5539310	mrpl11	2	1	1	0	1	0
ENSGACG00000017327	ChrIII	13672383	13693975	rock1	2	0	0	0	1	1
ENSGACG00000017644	ChrIII	14929551	14958963		2	0	0	0	2	0
ENSGACG00000018082	ChrXIV	11064738	11154619	whr0	2	0	0	0	2	0
ENSGACG00000019224	ChrIX	16048022	16056648	cep170ab	2	2	0	2	0	0
ENSGACG00000019481	ChrIV	25424476	25433284	krr1	2	0	0	0	1	1
ENSGACG00000000075	scaffold_99	201535	229070		1	0	0	0	1	0
ENSGACG00000000307	scaffold_27	48405	52111		1	0	0	0	1	0
ENSGACG00000000381	scaffold_131	232303	234144	rbm46	1	0	0	0	1	0
ENSGACG00000000554	scaffold_67	141528	172215	DPP6	1	1	1	0	0	0
ENSGACG00000000557	scaffold_27	1192061	1196661		1	0	0	0	0	1
ENSGACG00000000582	scaffold_67	364680	406885	CDH18	1	0	0	0	1	0
ENSGACG00000000634	scaffold_90	226420	229275	cradd	1	0	0	0	1	0
ENSGACG00000000950	scaffold_157	146047	149073	si:dkey-201i6.2	1	0	0	0	1	0
ENSGACG00000001063	scaffold_27	3053896	3067281	dstyk	1	0	0	0	0	1
ENSGACG00000001165	scaffold_129	135238	139395	tfr2	1	0	0	0	1	0
ENSGACG00000001382	scaffold_27	4081612	4132724	cadpsb	1	0	0	0	1	0
ENSGACG00000001468	scaffold_111	190733	194077	mgat1b	1	0	0	0	0	1
ENSGACG00000001581	scaffold_120	126130	128131	si:ch1073-184j22.2	1	0	0	0	0	1

ENSGACG00000001580	scaffold_169	57700	104630	nrp1b	1	0	0	0	1	0
ENSGACG00000001633	scaffold_120	253516	254412		1	0	0	0	0	1
ENSGACG00000001637	scaffold_106	301947	326476	aco1	1	1	0	1	0	0
ENSGACG00000001734	ChrXVI	2405778	2407622	kbtbd7	1	0	0	0	1	0
ENSGACG00000001745	scaffold_122	182930	185817	tlr18	1	0	0	0	0	1
ENSGACG00000001883	ChrXVI	2628609	2645144	klf12a	1	0	0	0	1	0
ENSGACG00000001892	ChrXVI	2776645	2777646	gpr18	1	1	0	1	0	0
ENSGACG00000001980	scaffold_149	22071	23010		1	1	0	1	0	0
ENSGACG00000001990	ChrX	1252132	1253742	zgc:91910	1	0	0	0	0	1
ENSGACG00000001974	ChrXXI	2122593	2149047	prkdc	1	0	0	0	1	0
ENSGACG00000002007	ChrXVI	3402359	3407495		1	0	0	0	0	1
ENSGACG00000002044	scaffold_214	65170	78666	fam69c	1	0	0	0	1	0
ENSGACG00000002216	ChrXVI	4428375	4457157	ADARB1	1	0	0	0	1	0
ENSGACG00000002328	ChrXVI	5502519	5557728	d0h7	1	0	0	0	1	0
ENSGACG00000002439	ChrVI	624617	631220	degs1	1	0	0	0	0	1
ENSGACG00000002542	ChrV	1182211	1207003	CHST15	1	1	1	0	0	0
ENSGACG00000002640	ChrV	1488301	1595530	dock1	1	0	0	0	1	0
ENSGACG00000002655	ChrXII	409174	434561		1	0	0	0	1	0
ENSGACG00000002755	ChrXVI	6491791	6504029	gpr39	1	0	0	0	1	0
ENSGACG00000002766	ChrXVI	6578389	6579905		1	0	0	0	1	0
ENSGACG00000002798	ChrXVI	6698167	6721328	cmss1	1	0	0	0	1	0
ENSGACG00000002794	ChrXXI	6761590	6775206	trpa1b	1	0	0	0	1	0
ENSGACG00000002797	ChrXII	657639	663452	rbm39b	1	0	0	0	1	0
ENSGACG00000002805	ChrXII	664631	666046	rh50	1	0	0	0	1	0
ENSGACG00000002820	ChrVI	1604348	1655313	INPP5A	1	0	0	0	1	0
ENSGACG00000002821	ChrXVI	6947968	6950857	r0seh2b	1	0	0	0	1	0
ENSGACG00000002889	ChrXVI	7108006	7109040	sox1a	1	0	0	0	1	0
ENSGACG00000002982	ChrXII	1118299	1128392	TMCC1 (1 of many)	1	0	0	0	0	1

ENSGACG00000003024	ChrXII	1350285	1355534	ddx23	1	0	0	0	1	0
ENSGACG00000003039	ChrXVII	106908	109258		1	0	0	0	1	0
ENSGACG00000003210	ChrXVII	474804	485236		1	0	0	0	1	0
ENSGACG00000003216	ChrVI	2793166	2802270	pik3ap1	1	0	0	0	1	0
ENSGACG00000003244	ChrX	4431116	4431815		1	0	0	0	0	1
ENSGACG00000003280	ChrX	4476813	4485782	rprd2a	1	0	0	0	1	0
ENSGACG00000003349	ChrXII	1948134	1950471		1	0	0	0	0	1
ENSGACG00000003467	ChrXX	759236	760746	apoa2	1	0	0	0	0	1
ENSGACG00000003513	ChrXVII	772640	787599	rims4	1	1	1	0	0	0
ENSGACG00000003533	ChrX	4941823	4965131	jarid2b	1	0	0	0	1	0
ENSGACG00000003552	ChrX	5093819	5117753	pard6gb	1	0	0	0	1	0
ENSGACG00000003560	ChrXIII	768298	822763	NR6A1	1	0	0	0	1	0
ENSGACG00000003616	ChrXII	2350366	2351344	pdyn	1	0	0	0	1	0
ENSGACG00000003621	ChrXXI	8263488	8269613	gyg1b	1	0	0	0	1	0
ENSGACG00000003642	ChrXIII	971041	971448	mrpl41	1	0	0	0	1	0
ENSGACG00000003641	ChrXVII	1100095	1107153	ahcy	1	0	0	0	0	1
ENSGACG00000003673	ChrVIII	1742873	1762035	crocc2	1	0	0	0	1	0
ENSGACG00000003793	ChrXVII	1220142	1250579	slmapa	1	0	0	0	1	0
ENSGACG00000003830	ChrXVI	8743067	8776300	ANOS1	1	0	0	0	1	0
ENSGACG00000003851	ChrXVI	8803555	8808879	gpr143	1	0	0	0	1	0
ENSGACG00000003974	ChrVI	4172491	4201696	b3gat2	1	0	0	0	1	0
ENSGACG00000004154	ChrVI	4697513	4739277	sipa1l2	1	0	0	0	1	0
ENSGACG00000004161	ChrX	6444050	6447473	cyp51	1	0	0	0	0	1
ENSGACG00000004237	ChrXVI	9502200	9533558	DOCK9 (1 of many)	1	0	0	0	0	1
ENSGACG00000004387	ChrX	6719206	6725533	znf687b	1	0	0	0	1	0
ENSGACG00000004411	ChrV	5205599	5280399	snx29	1	0	0	0	1	0
ENSGACG00000004431	ChrXII	3958137	3978532	ntsr1	1	1	0	1	0	0
ENSGACG00000004453	ChrXVIII	1071040	1077095	si:ch211-63o20.7	1	0	0	0	1	0

ENSGACG00000004448	ChrXVII	2225924	2233495	dhrs3b	1	0	0	0	1	0
ENSGACG00000004636	ChrI	372834	374001		1	0	0	0	1	0
ENSGACG00000004635	ChrXIII	2105329	2109391	si:dkey-193c22.1	1	0	0	0	1	0
ENSGACG00000004738	ChrXVII	2595419	2644876		1	0	0	0	1	0
ENSGACG00000004752	ChrXVII	2653110	2664476	si:ch211-286o17.1	1	0	0	0	1	0
ENSGACG00000004716	ChrX	7178557	7197519	si:dkeyp-120h9.1	1	0	0	0	0	1
ENSGACG00000004743	ChrXX	2589138	2594554	sqlea	1	0	0	0	1	0
ENSGACG00000004766	ChrXX	2597737	2628658	oxr1b	1	1	1	0	0	0
ENSGACG00000004773	ChrXII	4621502	4622140		1	0	0	0	1	0
ENSGACG00000004780	ChrXII	4743053	4896679	plx01a	1	1	1	0	0	0
ENSGACG00000004808	ChrXVI	10104986	10129378		1	0	0	0	1	0
ENSGACG00000004873	ChrXXI	10500632	10511464	CDH19	1	1	1	0	0	0
ENSGACG00000004940	ChrXVIII	1504942	1509134	serpi01l	1	0	0	0	0	1
ENSGACG00000005016	ChrVIII	3619176	3623161		1	0	0	0	1	0
ENSGACG00000005034	ChrXVII	3261730	3295915	0v1b	1	0	0	0	0	1
ENSGACG00000005087	ChrXXI	10930916	10935699	mastl	1	0	0	0	0	1
ENSGACG00000005138	ChrXI	818949	822342		1	0	0	0	1	0
ENSGACG00000005222	ChrXVII	3437445	3438929	kc02a	1	0	0	0	1	0
ENSGACG00000005224	ChrXVIII	2150130	2155377	gtf2a1	1	0	0	0	1	0
ENSGACG00000005280	ChrXVIII	2211718	2416099	nrxn3b	1	1	1	0	0	0
ENSGACG00000005395	ChrXIII	3541942	3609276	unc5db	1	0	0	0	1	0
ENSGACG00000005473	ChrXVII	3704985	3708788	ncoa5	1	0	0	0	0	1
ENSGACG00000005498	ChrXII	6282953	6382409		1	0	0	0	1	0
ENSGACG00000005579	ChrXII	6456672	6459151	casp9	1	1	1	0	0	0
ENSGACG00000005585	ChrI	1594281	1664168	si:ch211-276c2.2	1	0	0	0	0	1
ENSGACG00000005642	ChrXII	6730343	6739230		1	0	0	0	0	1
ENSGACG00000005690	ChrVIII	5061230	5065590	fetub	1	0	0	0	0	1
ENSGACG00000005826	ChrXV	2081586	2088261	pomt2	1	0	0	0	1	0
ENSGACG00000005871	ChrXI	2407492	2415100	0t15	1	1	1	0	0	0

ENSGACG00000005912	ChrXVII	4162499	4184565	eya2	1	0	0	0	1	0
ENSGACG00000005959	ChrXIII	4441375	4446248	CKMT2	1	0	0	0	1	0
ENSGACG00000005959	ChrXIII	4441375	4446248	CKMT2	1	0	0	0	0	1
ENSGACG00000006073	ChrXVI	11690439	11697554	SMARCAL1	1	0	0	0	1	0
ENSGACG00000006136	ChrI	2281697	2283639		1	0	0	0	1	0
ENSGACG00000006109	ChrXV	2485491	2488657	ACTA1 (1 of many)	1	0	0	0	1	0
ENSGACG00000006168	ChrXVIII	3331871	3333721	col10a1b	1	1	1	0	0	0
ENSGACG00000006159	ChrXII	7573896	7590952		1	0	0	0	1	0
ENSGACG00000006162	ChrV	8116939	8120965		1	0	0	0	0	1
ENSGACG00000006227	ChrI	2824077	2830150	rap1gap2a	1	0	0	0	1	0
ENSGACG00000006220	ChrXIII	4822900	4865643	DMXL1	1	0	0	0	1	0
ENSGACG00000006268	ChrXVIII	3894826	3965753	epha7	1	1	1	0	0	0
ENSGACG00000006305	ChrXIII	4975851	4979063	mapkapk5	1	0	0	0	1	0
ENSGACG00000006314	ChrXVI	12033164	12044262		1	1	1	0	0	0
ENSGACG00000006321	ChrI	3060604	3065797	p2rx8	1	0	0	0	1	0
ENSGACG00000006409	ChrXI	2907304	2924089	plppr2a	1	1	1	0	0	0
ENSGACG00000006410	ChrXVII	5112878	5123495	shmt2	1	0	0	0	1	0
ENSGACG00000006443	ChrXII	7864313	7874314	ppih	1	0	0	0	1	0
ENSGACG00000006475	ChrXVIII	4611333	4622904		1	0	0	0	0	1
ENSGACG00000006571	ChrX	9279918	9287935	casd1	1	1	0	1	0	0
ENSGACG00000006656	ChrXV	2935681	2938985		1	0	0	0	1	0
ENSGACG00000006652	ChrX	9301880	9334968	ppp1r9a	1	0	0	0	1	0
ENSGACG00000006740	ChrXI	3465800	3467727	si:dkeyp-69e1.8	1	0	0	0	0	1
ENSGACG00000006849	ChrX	9509713	9515132	mpp6b	1	0	0	0	0	1
ENSGACG00000006931	ChrXV	3674888	3699659		1	0	0	0	1	0
ENSGACG00000006954	ChrX	9600905	9605308		1	0	0	0	1	0
ENSGACG00000007068	ChrI	4543685	4582641	KC0B1	1	0	0	0	1	0
ENSGACG00000007130	ChrXX	8083957	8088889	cdcp1a	1	0	0	0	1	0

ENSGACG00000007220	ChrV	9430229	9431432	zgc:113090	1	0	0	0	0	1
ENSGACG00000007241	ChrXV	4150772	4153424	fam167b	1	0	0	0	1	0
ENSGACG00000007260	ChrXV	4158259	4161687	tdh2	1	0	0	0	1	0
ENSGACG00000007146	ChrXVI	14092977	14114066	dars	1	0	0	0	1	0
ENSGACG00000007270	ChrVIII	8208428	8335563		1	0	0	0	1	0
ENSGACG00000007280	ChrXV	4171868	4184504	clic4	1	0	0	0	0	1
ENSGACG00000007343	ChrX	10666995	10679875	col9a2	1	0	0	0	0	1
ENSGACG00000007372	ChrX	10807926	10961030	csmd2	1	0	0	0	0	1
ENSGACG00000007442	ChrXVII	6294895	6297552		1	0	0	0	0	1
ENSGACG00000007613	ChrI	5854274	5864978	baz1b	1	0	0	0	1	0
ENSGACG00000007693	ChrI	5937570	5939340	zgc:103681	1	0	0	0	1	0
ENSGACG00000007711	ChrXX	9099195	9126875	nphs1	1	0	0	0	1	0
ENSGACG00000007746	ChrXV	4848926	4850768	tmed8	1	0	0	0	0	1
ENSGACG00000007745	ChrXIII	7495399	7509368	EGFLAM	1	0	0	0	0	1
ENSGACG00000007819	ChrX	12069833	12099979	foxo6b	1	1	1	0	0	0
ENSGACG00000007825	ChrI	6236909	6237643	a0pc15	1	0	0	0	1	0
ENSGACG00000007835	ChrV	9774074	9779340		1	0	0	0	1	0
ENSGACG00000007863	ChrVIII	8897020	8952064	nek7	1	0	0	0	1	0
ENSGACG00000007953	ChrI	6283524	6297921	NOX4	1	0	0	0	1	0
ENSGACG00000008143	ChrXVI	15980825	15981403	arl4cb	1	0	0	0	1	0
ENSGACG00000008201	ChrVIII	9458245	9463856		1	0	0	0	1	0
ENSGACG00000008207	ChrI	6847350	6887280	si:ch211-248g20.5	1	0	0	0	1	0
ENSGACG00000008217	ChrVIII	9502643	9506528		1	0	0	0	0	1
ENSGACG00000008225	ChrX	12619123	12643081	grb10a	1	1	1	0	0	0
ENSGACG00000008252	ChrX	12695029	12695737		1	0	0	0	0	1
ENSGACG00000008350	ChrXIII	8375895	8401616	smtnb	1	0	0	0	1	0
ENSGACG00000008540	ChrXVI	16966117	16967188		1	0	0	0	0	1
ENSGACG00000008628	ChrXVII	7986609	8018375		1	1	1	0	0	0
ENSGACG00000008689	ChrV	10760247	10779914		1	1	1	0	0	0

ENSGACG00000008798	ChrX	13132413	13138599	si:ch1073-296d18.1	1	0	0	0	0	1
ENSGACG00000009135	ChrVI	10670867	10727803	pcdh15a	1	0	0	0	0	1
ENSGACG00000009146	ChrXIII	9082014	9083453	fam222a	1	1	1	0	0	0
ENSGACG00000009211	ChrXVIII	8793962	8796629	si:dkey-65b13.13	1	0	0	0	1	0
ENSGACG00000009251	ChrVIII	11476798	11480609	htr2b	1	0	0	0	1	0
ENSGACG00000009248	ChrXVIII	8940535	8942987		1	0	0	0	1	0
ENSGACG00000009346	ChrVIII	11764463	11771444	srsf11	1	1	1	0	0	0
ENSGACG00000009393	ChrXVII	9412190	9495385	FAM19A1	1	0	0	0	1	0
ENSGACG00000009627	ChrXX	11403589	11405982	si:ch73-54f23.4	1	0	0	0	1	0
ENSGACG00000009653	ChrX	14747980	14771097	znf976	1	1	0	1	0	0
ENSGACG00000009650	ChrXV	7669664	7725769	qkia	1	0	0	0	1	0
ENSGACG00000009733	ChrXIII	9474478	9479229	D0JB5	1	1	0	1	0	0
ENSGACG00000009746	ChrX	14996770	15014430		1	0	0	0	1	0
ENSGACG00000009796	ChrXVIII	9441961	9443603	kcns3b	1	0	0	0	1	0
ENSGACG00000009870	ChrXIII	9641184	9654500	serinc5	1	0	0	0	1	0
ENSGACG00000009920	ChrXV	8067468	8068457		1	0	0	0	0	1
ENSGACG00000009923	ChrX	15274191	15282234		1	0	0	0	1	0
ENSGACG00000009968	ChrXV	8210188	8222680		1	0	0	0	1	0
ENSGACG00000009983	ChrXVII	10033214	10065567	cdh4	1	0	0	0	0	1
ENSGACG00000009991	ChrXVIII	9665614	9671172	tagapb	1	0	0	0	0	1
ENSGACG00000010108	ChrVI	12267753	12268331	grem2a	1	0	0	0	1	0
ENSGACG00000010176	ChrXIII	9941933	9943709		1	0	0	0	1	0
ENSGACG00000010260	ChrXV	8712213	8715682	manea	1	0	0	0	1	0
ENSGACG00000010297	ChrVI	12537633	12538976		1	0	0	0	0	1
ENSGACG00000010299	ChrXVIII	10233449	10270176	daam1b	1	0	0	0	1	0
ENSGACG00000010494	ChrVIII	13255573	13285202	si:zfos-588f8.1	1	0	0	0	1	0
ENSGACG00000010507	ChrXV	9224970	9273325	zgc:154061	1	0	0	0	1	0
ENSGACG00000010532	ChrXV	9468781	9481444	spred1	1	0	0	0	1	0

ENSGACG00000010542	ChrXV	9490435	9501192	ptpn21	1	0	0	0	1	0
ENSGACG00000010528	ChrVIII	13335579	13342295	ncln	1	0	0	0	1	0
ENSGACG00000010643	ChrXVII	10920133	10925973		1	0	0	0	0	1
ENSGACG00000010835	ChrXV	9862819	9867105	slc35g2a	1	0	0	0	0	1
ENSGACG00000010886	ChrXIII	12045266	12049108	si:dkey-32e23.6	1	0	0	0	1	0
ENSGACG00000010951	ChrI	12038391	12056617	si:dkey-243i1.1	1	0	0	0	1	0
ENSGACG00000011003	ChrVI	14099672	14126488	kif20ba	1	0	0	0	1	0
ENSGACG00000011145	ChrVIII	13948367	13950370	dmrta2	1	1	1	0	0	0
ENSGACG00000011191	ChrVI	14358268	14389292	slc30a6	1	0	0	0	1	0
ENSGACG00000011235	ChrXV	10608343	10624532	crip2	1	0	0	0	1	0
ENSGACG00000011269	ChrXV	10677665	10678117	tmem229b	1	1	1	0	0	0
ENSGACG00000011225	ChrVI	14477471	14520782	SPTBN1	1	0	0	0	1	0
ENSGACG00000011313	ChrXI	9901388	9903421	vasnb	1	0	0	0	1	0
ENSGACG00000011355	ChrXVIII	11589919	11605386	nhsl1b	1	0	0	0	1	0
ENSGACG00000011353	ChrXII	15159858	15171195	mical1	1	0	0	0	0	1
ENSGACG00000011470	ChrVIII	14490182	14491442		1	0	0	0	1	0
ENSGACG00000011556	ChrXIII	13107695	13111326	rorb	1	1	1	0	0	0
ENSGACG00000011602	ChrXVII	12652011	12662065	LAMB3	1	0	0	0	1	0
ENSGACG00000011628	ChrVI	15521491	15543739	nckap1	1	0	0	0	1	0
ENSGACG00000011643	ChrXI	10486001	10490341	cacng5b	1	0	0	0	1	0
ENSGACG00000011670	ChrXX	12649677	12655617	REC8	1	1	1	0	0	0
ENSGACG00000011679	ChrI	13725256	13739469	trim3a	1	0	0	0	0	1
ENSGACG00000011694	ChrXI	10642924	10653747	axin2	1	0	0	0	1	0
ENSGACG00000011697	ChrI	13774012	13834603	dchs1a	1	0	0	0	1	0
ENSGACG00000011710	ChrXV	11490059	11493449		1	0	0	0	0	1
ENSGACG00000011711	ChrVIII	15092507	15103135	olfml2ba	1	0	0	0	0	1
ENSGACG00000011770	ChrXVII	13695917	13762426	pdzrn3b	1	0	0	0	0	1
ENSGACG00000011933	ChrVI	16265135	16268160	tmem41ab	1	0	0	0	0	1
ENSGACG00000012010	ChrXVIII	12857870	12861754		1	0	0	0	0	1

ENSGACG00000012063	ChrXV	11951816	11991325	actn1	1	0	0	0	1	0
ENSGACG00000012128	ChrI	14736944	14767457		1	0	0	0	1	0
ENSGACG00000012153	ChrXIII	13909513	13913900	asb6	1	0	0	0	1	0
ENSGACG00000012241	ChrXVIII	13379496	13382944	rad51	1	0	0	0	0	1
ENSGACG00000012270	ChrXVIII	13405473	13406295		1	0	0	0	1	0
ENSGACG00000012283	ChrI	15424209	15453953	cdon	1	0	0	0	1	0
ENSGACG00000012322	ChrXVIII	13649915	13680123	runx2b	1	1	0	1	0	0
ENSGACG00000012330	ChrVIII	15635360	15643570	rgl1	1	0	0	0	0	1
ENSGACG00000012385	ChrXVIII	13772879	13784829	mfsd2b	1	0	0	0	1	0
ENSGACG00000012455	ChrXI	11660812	11665052		1	0	0	0	1	0
ENSGACG00000012442	ChrVIII	15749226	15764692	notch2	1	0	0	0	1	0
ENSGACG00000012512	ChrXIII	14598285	14599898	lrrtm4l2	1	1	0	1	0	0
ENSGACG00000012503	ChrXVIII	14203391	14223968	PLCB4	1	0	0	0	1	0
ENSGACG00000012587	ChrXI	11922124	11924367	wfikkn2a	1	0	0	0	1	0
ENSGACG00000012609	ChrXI	12065873	12068920		1	0	0	0	1	0
ENSGACG00000012641	ChrXX	14272032	14305104		1	0	0	0	0	1
ENSGACG00000012641	ChrXX	14272032	14305104		1	0	0	0	1	0
ENSGACG00000012655	scaffold_98	371516	372691		1	0	0	0	1	0
ENSGACG00000012760	ChrVIII	16214608	16225148	si:dkey-110c1.10	1	1	1	0	0	0
ENSGACG00000012876	ChrVIII	16469522	16475075	rgmd	1	0	0	0	1	0
ENSGACG00000012938	ChrXVIII	14983710	14985734	chac1	1	0	0	0	0	1
ENSGACG00000012995	ChrVIII	16649364	16651755	fstl3	1	0	0	0	1	0
ENSGACG00000012997	ChrXII	17297684	17327439	plch2a	1	0	0	0	1	0
ENSGACG00000013040	ChrXVIII	15062591	15066857	stx7l	1	0	0	0	0	1
ENSGACG00000013071	ChrVIII	16932446	16936340	tmem161a	1	0	0	0	1	0
ENSGACG00000013118	ChrXV	15401988	15432525	BCL11B	1	0	0	0	1	0
ENSGACG00000013228	ChrI	17134277	17138229		1	0	0	0	0	1
ENSGACG00000013248	ChrXVIII	15399767	15421191	nkain2	1	0	0	0	1	0
ENSGACG00000013252	ChrXVIII	15429123	15433946	rnf217	1	1	0	1	0	0

ENSGACG00000013255	ChrXV	15759319	15781086	MYT1L	1	0	0	0	1	0
ENSGACG00000013218	ChrXI	12798318	12805251		1	0	0	0	1	0
ENSGACG00000013273	ChrXIII	16131688	16140819	arhgap25	1	0	0	0	1	0
ENSGACG00000013297	ChrVIII	17242910	17245367	rab11ba	1	1	1	0	0	0
ENSGACG00000013422	scaffold_61	545965	563244	fancl	1	0	0	0	1	0
ENSGACG00000013463	ChrXIII	16423554	16426950	rad9b	1	0	0	0	1	0
ENSGACG00000013478	scaffold_196	78142	81595		1	0	0	0	0	1
ENSGACG00000013488	ChrXI	13112136	13116786	lrrc4ba	1	0	0	0	0	1
ENSGACG00000013504	ChrXX	16295857	16300154	hormad1	1	1	1	0	0	0
ENSGACG00000013506	ChrXI	13157415	13172421	syt3	1	0	0	0	1	0
ENSGACG00000013523	ChrIII	1578911	1598618		1	1	1	0	0	0
ENSGACG00000013636	ChrVIII	17679353	17685427	zbtb11	1	0	0	0	1	0
ENSGACG00000013639	ChrXX	16926113	16927065	fabp10a	1	0	0	0	0	1
ENSGACG00000013787	ChrXI	13506470	13516007	tyk2	1	0	0	0	0	1
ENSGACG00000013869	ChrXI	13640461	13641149		1	0	0	0	0	1
ENSGACG00000013977	ChrVIII	18160785	18167767	SCML2 (1 of many)	1	0	0	0	0	1
ENSGACG00000014073	ChrXIII	18076604	18087798	nf2a	1	0	0	0	1	0
ENSGACG00000014104	ChrVIII	18357770	18360435	cbr4	1	0	0	0	1	0
ENSGACG00000014252	ChrXIII	18343004	18348481	suds3	1	1	1	0	0	0
ENSGACG00000014384	ChrXI	14979751	14982832		1	0	0	0	1	0
ENSGACG00000014435	ChrIII	4386571	4396709	tgm1l1	1	0	0	0	1	0
ENSGACG00000014548	scaffold_240	819	11444	dirc2	1	1	1	0	0	0
ENSGACG00000014576	ChrI	22242655	22244658		1	0	0	0	1	0
ENSGACG00000014580	ChrI	22269225	22284819	GPM6B (1 of many)	1	0	0	0	1	0
ENSGACG00000014627	ChrI	22403687	22410614	cbsa	1	0	0	0	1	0
ENSGACG00000014832	ChrII	5125390	5127551	gnrhr2	1	0	0	0	1	0
ENSGACG00000014892	ChrIII	6063746	6077226		1	0	0	0	1	0
ENSGACG00000014902	ChrII	5427953	5521857	itfg1	1	0	0	0	1	0

ENSGACG00000014926	ChrI	23288291	23341962	zranb3	1	0	0	0	1	0
ENSGACG00000014935	ChrXI	16311211	16319300		1	1	0	1	0	0
ENSGACG00000015001	ChrXI	16612089	16615925	sgf29	1	0	0	0	0	1
ENSGACG00000015011	ChrXI	16647398	16653948		1	0	0	0	0	1
ENSGACG00000015054	ChrI	24715179	24716868		1	0	0	0	1	0
ENSGACG00000015079	ChrI	25173862	25177412	cyyr1	1	0	0	0	0	1
ENSGACG00000015135	ChrI	25487311	25491104	prkag3a	1	1	1	0	0	0
ENSGACG00000015240	ChrI	26613808	26622627	adarb1a	1	0	0	0	1	0
ENSGACG00000015269	scaffold_48	760666	799826	myo15b	1	0	0	0	1	0
ENSGACG00000015412	scaffold_48	1674248	1685118	ipmkb	1	0	0	0	1	0
ENSGACG00000015490	ChrXIV	174415	177418	ufc1	1	0	0	0	1	0
ENSGACG00000015736	ChrXIV	723869	725546	mzt2b	1	0	0	0	0	1
ENSGACG00000015791	ChrII	11127315	11148839	PHLPP2	1	1	1	0	0	0
ENSGACG00000015813	ChrXIV	1195794	1214842		1	0	0	0	0	1
ENSGACG00000015831	ChrII	11422567	11478619	tcf12	1	0	0	0	1	0
ENSGACG00000015862	ChrXIV	1681568	1684183	npffr2b	1	0	0	0	0	1
ENSGACG00000015920	ChrXIV	2455047	2525326	lamc3	1	0	0	0	1	0
ENSGACG00000016065	ChrIII	9230992	9232131	b3gnt5b	1	0	0	0	0	1
ENSGACG00000016073	ChrIX	1334639	1357056		1	1	1	0	0	0
ENSGACG00000016204	ChrIX	1923297	1930876	ncapg	1	0	0	0	1	0
ENSGACG00000016262	ChrII	14069329	14225725	ush2a	1	0	0	0	1	0
ENSGACG00000016328	ChrIX	3038147	3051468		1	0	0	0	1	0
ENSGACG00000016451	ChrII	15348070	15353934	CTSH	1	0	0	0	0	1
ENSGACG00000016473	ChrXIV	3993506	3998272	prlra	1	0	0	0	1	0
ENSGACG00000016484	ChrII	15471688	15483464		1	0	0	0	1	0
ENSGACG00000016513	ChrIV	2044920	2048363	tdo2a	1	0	0	0	1	0
ENSGACG00000016529	ChrIII	10691525	10715730	st6gal0c3	1	0	0	0	0	1
ENSGACG00000016530	ChrIX	4476469	4478563	rdh8b	1	1	0	1	0	0
ENSGACG00000016539	ChrII	15944730	15950252	scamp5b	1	0	0	0	1	0

ENSGACG00000016553	ChrII	16198253	16219272	SHF	1	0	0	0	1	0
ENSGACG00000016589	ChrIX	4587599	4591165		1	0	0	0	0	1
ENSGACG00000016634	ChrIV	2582164	2682609	FSTL5	1	0	0	0	1	0
ENSGACG00000016731	ChrIV	3340848	3411728	SPATA5	1	0	0	0	0	1
ENSGACG00000016800	ChrIX	5319576	5323725	pdia2	1	0	0	0	1	0
ENSGACG00000016846	ChrIX	5456262	5476760		1	1	1	0	0	0
ENSGACG00000016849	ChrXIV	6012778	6022500		1	0	0	0	0	1
ENSGACG00000016943	ChrXIV	6450832	6461836	cabp7b	1	0	0	0	1	0
ENSGACG00000016997	ChrII	19330861	19331520	senp8	1	0	0	0	1	0
ENSGACG00000017036	ChrXIV	6661932	6668110	rnf34b	1	0	0	0	1	0
ENSGACG00000017075	ChrII	19771601	19780222	zdhhc13	1	0	0	0	1	0
ENSGACG00000017087	ChrII	19884513	19943913	0v2a	1	0	0	0	1	0
ENSGACG00000017093	ChrXIV	6818762	6821900	trim69	1	0	0	0	0	1
ENSGACG00000017160	ChrII	21080854	21084821		1	0	0	0	1	0
ENSGACG00000017179	ChrIII	13106071	13116555	fyco1b	1	0	0	0	1	0
ENSGACG00000017190	ChrIV	5872021	5884796	mcf2a	1	0	0	0	1	0
ENSGACG00000017209	ChrXIV	6969603	6972066	pcyox1	1	0	0	0	1	0
ENSGACG00000017250	ChrIX	7131393	7145303	gab1	1	0	0	0	1	0
ENSGACG00000017287	ChrIII	13454527	13465167	mmp16b	1	1	1	0	0	0
ENSGACG00000017291	ChrIX	7339426	7343930	elmod2	1	0	0	0	1	0
ENSGACG00000017300	ChrIII	13558657	13562089	gata6	1	0	0	0	1	0
ENSGACG00000017313	ChrII	21709930	21734771	MEGF11 (1 of many)	1	1	1	0	0	0
ENSGACG00000017331	ChrII	21831393	21842872	samd4a	1	0	0	0	1	0
ENSGACG00000017352	ChrII	21934736	21940961		1	0	0	0	1	0
ENSGACG00000017402	ChrIII	13870170	13884783	pth1ra	1	0	0	0	1	0
ENSGACG00000017480	ChrIII	14138063	14147391	WDR37 (1 of many)	1	0	0	0	1	0
ENSGACG00000017537	ChrXIV	8099548	8156144	bcr	1	0	0	0	1	0
ENSGACG00000017598	ChrXIV	8297103	8367291	si:dkey-112m2.1	1	0	0	0	1	0

ENSGACG00000017645	ChrII	23237993	23278096	vps13c	1	0	0	0	1	0
ENSGACG00000017680	ChrXIV	8907355	8911332	stx2a	1	0	0	0	1	0
ENSGACG00000017696	ChrIII	15339421	15358805		1	0	0	0	1	0
ENSGACG00000017662	ChrIX	8582121	8588508	acsl1a	1	0	0	0	1	0
ENSGACG00000017748	scaffold_137	157404	162580	plekhf2	1	0	0	0	1	0
ENSGACG00000017791	ChrXIV	9332963	9357146		1	0	0	0	1	0
ENSGACG00000017788	ChrIV	9240071	9244174	p2rx3a	1	0	0	0	1	0
ENSGACG00000017924	ChrXIV	10388550	10398180	ghrb	1	0	0	0	1	0
ENSGACG00000018029	ChrIV	11001491	11005265	myoz3a	1	0	0	0	0	1
ENSGACG00000018030	ChrIV	11007288	11014249		1	0	0	0	1	0
ENSGACG00000018174	ChrXIV	11642479	11658752		1	0	0	0	1	0
ENSGACG00000018228	ChrIX	10649611	10672955		1	0	0	0	1	0
ENSGACG00000018233	ChrIX	10713754	10725152	pdcd11	1	1	1	0	0	0
ENSGACG00000018231	ChrIV	12013740	12034920	abcb7	1	1	0	1	0	0
ENSGACG00000018319	ChrIV	12891471	12904168	ergic1	1	0	0	0	1	0
ENSGACG00000018371	ChrXIV	14084346	14091899	thap1	1	0	0	0	1	0
ENSGACG00000018389	ChrIV	13479246	13481484		1	1	1	0	0	0
ENSGACG00000018400	ChrIX	11461137	11465372		1	0	0	0	0	1
ENSGACG00000018496	ChrIV	15032996	15039498	si:ch211-160j14,2	1	0	0	0	0	1
ENSGACG00000018568	ChrIX	13057908	13059965	si:dkeyp-110c7.8	1	0	0	0	0	1
ENSGACG00000018572	ChrIV	15735707	15737284	p2ry10	1	0	0	0	1	0
ENSGACG00000018663	ChrIV	16118651	16126776	tmem173	1	0	0	0	1	0
ENSGACG00000018722	scaffold_208	83156	90702	iffo1a	1	1	1	0	0	0
ENSGACG00000018779	ChrVII	1036400	1060359	col4a6	1	0	0	0	1	0
ENSGACG00000018798	ChrIX	14552729	14553871		1	0	0	0	0	1
ENSGACG00000018802	ChrVII	1133367	1137045		1	0	0	0	1	0
ENSGACG00000018819	ChrVII	1327429	1330995		1	1	1	0	0	0
ENSGACG00000018862	ChrIV	18656262	18660392	gxylt1b	1	0	0	0	1	0
ENSGACG00000018867	ChrVII	1452630	1459173	khnyn	1	0	0	0	0	1

ENSGACG00000018927	ChrIV	19504962	19515715		1	0	0	0	0	1
ENSGACG00000018991	ChrVII	2259206	2267772	zgc:55262	1	1	1	0	0	0
ENSGACG00000019043	ChrIX	15241216	15246931	pycr1b	1	0	0	0	1	0
ENSGACG00000019057	ChrVII	2467974	2472162	mfap3l	1	0	0	0	1	0
ENSGACG00000019125	ChrIV	21818906	21878888	SYN3	1	0	0	0	1	0
ENSGACG00000019166	ChrVII	2903189	2904509		1	0	0	0	1	0
ENSGACG00000019183	ChrIV	22236334	22237384		1	0	0	0	1	0
ENSGACG00000019227	ChrIV	22899577	22913555	mkrn1	1	0	0	0	0	1
ENSGACG00000019253	ChrIV	23209606	23250489		1	1	1	0	0	0
ENSGACG00000019295	ChrVII	3222487	3232052	tbc1d19	1	0	0	0	0	1
ENSGACG00000019316	ChrVII	4025500	4027085	cnpy4	1	0	0	0	1	0
ENSGACG00000019362	ChrVII	4248635	4260779	neurl4	1	0	0	0	1	0
ENSGACG00000019440	ChrIV	24711763	24712875		1	0	0	0	1	0
ENSGACG00000019443	ChrVII	4842549	4846809	mfsd8	1	0	0	0	1	0
ENSGACG00000019462	ChrIX	17689001	17750587		1	0	0	0	1	0
ENSGACG00000019485	ChrIV	25436299	25450435	cmah	1	0	0	0	1	0
ENSGACG00000019509	ChrIV	25585353	25601311	ANO4	1	0	0	0	1	0
ENSGACG00000019513	ChrIX	17973555	17980601		1	1	1	0	0	0
ENSGACG00000019526	ChrIX	18022842	18029582		1	1	1	0	0	0
ENSGACG00000019550	ChrIX	18092917	18100457		1	1	1	0	0	0
ENSGACG00000019679	ChrIV	28725325	28735256		1	0	0	0	1	0
ENSGACG00000019704	ChrIX	18828407	18830791	btbd3a	1	1	0	1	0	0
ENSGACG00000019718	ChrIV	29140176	29301388	plx04	1	0	0	0	1	0
ENSGACG00000019746	ChrIV	29700807	29758980	iqsec3a	1	0	0	0	0	1
ENSGACG00000019759	ChrIV	29797492	29817516	tmtc2a	1	0	0	0	0	1
ENSGACG00000019772	ChrIV	29858075	29865955	PRMT8 (1 of many)	1	1	1	0	0	0
ENSGACG00000019855	ChrIV	30901709	30906129	arfgap3	1	0	0	0	0	1
ENSGACG00000019956	ChrIV	31331071	31344595	sema3c	1	0	0	0	1	0

ENSGACG00000020002	ChrIX	20150078	20153043		1	0	0	0	1	0
ENSGACG00000020034	ChrIV	31999298	32000748	tnnt2c	1	1	1	0	0	0
ENSGACG00000020043	ChrVII	10180846	10198706		1	0	0	0	1	0
ENSGACG00000020068	ChrIV	32329916	32332578	ERI1	1	0	0	0	0	1
ENSGACG00000020174	ChrVII	12750327	12755673	capns1a	1	0	0	0	1	0
ENSGACG00000020401	ChrVII	17997085	18001531	si:ch211-137i24.10	1	0	0	0	0	1
ENSGACG00000020403	ChrVII	18007253	18010078	slc25a35	1	0	0	0	0	1
ENSGACG00000020493	ChrVII	19246409	19250932	NIPS0P2	1	0	0	0	0	1
ENSGACG00000020569	ChrVII	20326685	20369400	frem2a	1	0	0	0	0	1
ENSGACG00000020634	ChrVII	21614701	21623583	nectin1a	1	0	0	0	1	0
ENSGACG00000020724	ChrVII	23139706	23143482	yipf5	1	0	0	0	1	0
ENSGACG00000020725	ChrVII	23164545	23201619	NR3C1 (1 of many)	1	1	0	1	0	0
ENSGACG00000020726	ChrVII	23208162	23253092	ARHGAP26	1	0	0	0	1	0
ENSGACG00000020734	ChrVII	23362870	23385166	aff4	1	0	0	0	1	0
ENSGACG00000020754	ChrVII	24052097	24062908	mybbp1a	1	1	1	0	0	0
ENSGACG00000020770	ChrVII	24240056	24244002	htr3a	1	0	0	0	1	0
ENSGACG00000020803	ChrVII	25380828	25384895		1	0	0	0	0	1
ENSGACG00000020838	ChrVII	26287744	26291180		1	0	0	0	1	0
ENSGACG00000020848	ChrVII	26791004	26845216	doc2b	1	1	1	0	0	0
ENSGACG00000020852	ChrVII	26876308	26878683		1	0	0	0	1	0
ENSGACG00000021530	ChrIII	13882346	13882414		1	0	0	0	1	0
ENSGACG00000022165	ChrV	11138845	11138931		1	0	0	0	1	0

Table S3A. Tukey post hoc test results for survival rate. Significant comparisons are highlighted in bold.

Comparison	Estimate	Std. Error	z value	Pr(>|z|)
**WG_33 - control**	**-0.73765**	**0.26233**	**-2.812**	**0.03876**
**WG_6 - control**	-0.40963	0.27564	-1.486	0.56738
**TG_33 - control**	**-144.421**	**0.25161**	**-5.740**	**< 0.001**
**TG_06 - control**	-0.07637	0.26508	-0.288	0.99847
**WG_06 - WG_33**	0.32802	0.24052	1.364	0.64713
**TG_33 - WG_33**	**-0.70656**	**0.21261**	**-3.323**	**0.00769**
**TG_6 - WG_33**	**0.66128**	**0.22859**	**2.893**	**0.03051**
**TG_33 - WG_6**	**-103.457**	**0.23058**	**-4.487**	**< 0.001**
**TG_6 - WG_6**	0.33327	0.24674	1.351	0.65554
**TG_6 - TG_33**	**136.784**	**0.19398**	**7.052**	**< 0.001**

Table S3B. Tukey post hoc test results for SDL. Significant comparisons are highlighted in bold.

Comparison	Estimate	Std. Error	z value	Pr(>|z|)
**WG_33 - control**	-0.002895	0.056225	-0.051	10.000
**WG_6 - control**	0.074989	0.055932	1.341	0.6649
**TG_33 - control**	0.029907	0.059175	0.505	0.9868
**TG_06 - control**	**0.172371**	**0.059205**	**2.911**	**0.0296**
**WG_06 - WG_33**	0.077884	0.056181	1.386	0.6357
**TG_33 - WG_33**	0.032802	0.059405	0.552	0.9816
**TG_6 - WG_33**	**0.175265**	**0.059467**	**2.947**	**0.0265**
**TG_33 - WG_6**	-0.045082	0.058994	-0.764	0.9406
**TG_6 - WG_6**	0.097382	0.058991	1.651	0.4640
**TG_6 - TG_33**	0.142464	0.055749	2.555	0.0786

Table S3C. Tukey post hoc test results for HSI. Significant comparisons are highlighted in bold.

Comparison	Estimate	Std. Error	z value	Pr(>|z|)
**WG_33 - control**	-0.13514	0.11511	-1.174	0.76590
**WG_6 - control**	0.26415	0.11417	2.314	0.14025
**TG_33 - control**	-0.05518	0.11950	-0.462	0.99064
**TG_06 - control**	**0.40834**	**0.11958**	**3.415**	**0.00574**
**WG_06 - WG_33**	**0.39929**	**0.11504**	**3.471**	**0.00464**
**TG_33 - WG_33**	0.07996	0.12033	0.665	0.96384
**TG_6 - WG_33**	**0.54348**	**0.12049**	**4.511**	**< 0.001**
**TG_33 - WG_6**	-0.31933	0.11909	-2.681	0.05654
**TG_6 - WG_6**	0.14419	0.11907	1.211	0.74460
**TG_6 - TG_33**	**0.46352**	**0.11373**	**4.076**	**< 0.001**

Table S3D. Tukey post hoc test results for total weight. Significant comparisons are highlighted in bold.

Comparison	Estimate	Std. Error	z value	Pr(>|z|)
**WG_33 - control**	0.004969	0.015050	0.330	0.9974
**WG_6 - control**	0.025076	0.014925	1.680	0.4456
**TG_33 - control**	0.001846	0.015738	0.117	10.000
**TG_06 - control**	**0.045953**	**0.015752**	**2.917**	**0.0290**
**WG_06 - WG_33**	0.020106	0.015039	1.337	0.6674
**TG_33 - WG_33**	-0.003123	0.015840	-0.197	0.9997
**TG_6 - WG_33**	0.040984	0.015871	2.582	0.0734
**TG_33 - WG_6**	-0.023230	0.015661	-1.483	0.5727
**TG_6 - WG_6**	0.020877	0.015659	1.333	0.6697
**TG_6 - TG_33**	**0.044107**	**0.014845**	**2.971**	**0.0247**

Table S4. Summary statistics for whole-genome resequencing of wild-caught sticklebacks. Six stickleback populations (KIE = Kiel, NYN = Nynäshamn, SYL= Sylt, FAL = Falsterbo, LET = Letipea, BAR = Barsta) were sampled at the corresponding latitude and longitude and salinity. Fish ID is given, along with standard length and sex.

ID	sex	site name	latitude	longitude	salinity	standard length (mm)	total reads	reads aligend	aligned reads in pairs	high quality reads	mean coverage	sd coverage	mean insert size
S1	female	FAL	55.412955	12.931189	9.8	31	53099898	51454273	51059422	46005389	13.77	8.14	389.83
S10	male	SYL	55.016166	8.439550	28.9	24	59249088	57721239	57309340	51392222	15.29	8.75	378.09
S11	male	LET	59.5521	26.60826	4.3	24	64732112	62992502	62514646	55977942	16.47	9.16	379.76
S12	male	LET	59.5521	26.60826	4.3	28	60505260	58806659	58363258	52747595	15.54	8.74	368.95
S13	female	LET	59.5521	26.60826	4.3	40	46451078	45136076	44795792	40774872	12.08	7.18	371.11
S14	female	LET	59.5521	26.60826	4.3	28	52566384	51070498	50676508	46196326	13.6	7.77	361.36
S15	female	FAL	55.412955	12.931189	9.8	32	53175370	51740418	51356498	45760642	13.58	8.07	367.76
S16	female	FAL	55.412955	12.931189	9.8	31	54746754	53085246	52694874	47758257	14.11	8.07	368.2
S17	female	SYL	55.016166	8.439550	28.9	25	42687172	41590244	41276164	37539956	11.26	6.83	383.5
S18	female	SYL	55.016166	8.439550	28.9	23	55674944	54215662	53789138	48770957	14.52	8.22	374
S19	female	KIE	54.436621	10.17228	18	33	51339478	49896745	49534292	44841451	13.45	7.83	378.17
S2	female	FAL	55.412955	12.931189	9.8	31	67830816	65879708	65386846	59023635	17.5	9.33	383.05
S20	female	KIE	54.436621	10.17228	18	35	45388352	44131200	43780040	39724153	11.81	7.19	363.19
S21	male	NYN	58.8790698	17.935061	6	25	62740566	60992914	60552808	55067013	16.42	8.8	378.55
S22	male	NYN	58.8790698	17.935061	6	27	58127744	56500828	56070618	51359209	15.33	8.33	380.72
S23	male	BAR	62.8630833	18.3975	4.6	17	66400620	64557384	64076342	57948807	17.25	9.29	381.66
S24	male	BAR	62.8630833	18.3975	4.6	19	52888922	51354675	50965362	45750005	13.66	8.04	377.71
S25	female	BAR	62.8630833	18.3975	4.6	19	55355880	53867446	53461396	48579009	14.63	8.13	383.57
S26	female	BAR	62.8630833	18.3975	4.6	18	60545964	58904170	58457402	52851197	15.76	8.69	382.32
S27	female	LET	59.5521	26.60826	4.3	27	48256944	46901465	46558408	42198012	12.62	7.42	376.13
S28	female	LET	59.5521	26.60826	4.3	27	48321396	46997345	46655746	42196863	12.59	7.47	372.53

S29	female	FAL	55.412955	12.931189	9.8	31	53423426	51720960	51334050	46751683	14.07	7.85	382.41
S3	female	KIE	54.436621	10.17228	18	33	51511534	50109217	49751260	44785482	13.23	7.75	371.95
S30	female	FAL	55.412955	12.931189	9.8	31	62572564	60802237	60355732	54743424	16.31	8.85	382.79
S31	female	SYL	55.016166	8.439550	28.9	24	52336108	51000812	50630182	45718213	13.74	7.89	383.56
S32	female	SYL	55.016166	8.439550	28.9	25	50659258	49422266	49071064	44058329	13.21	7.87	376.03
S33	female	KIE	54.436621	10.17228	18	29	59992552	58304582	57854052	52465334	15.72	8.69	390.69
S34	female	KIE	54.436621	10.17228	18	29	52958328	51617586	51231202	46633547	14.08	7.96	395.11
S35	male	NYN	58.8790698	17.935061	6	39	54066258	52535615	52111994	46944359	13.96	8.12	385.2
S36	male	NYN	58.8790698	17.935061	6	26	58979382	57446489	57021398	52137658	15.58	8.42	377.63
S37	female	NYN	58.8790698	17.935061	6	53	60917462	59237862	58787416	53314193	15.88	8.72	386.29
S38	female	NYN	58.8790698	17.935061	6	32	66539062	64563355	64037068	57547546	17.02	9.34	379.2
S39	female	BAR	62.8630833	18.3975	4.6	18	53386772	51907116	51493034	46685925	13.92	7.93	383.5
S4	female	KIE	54.436621	10.17228	18	34	53563882	52156214	51776690	46822429	13.9	8.02	373.12
S40	female	BAR	62.8630833	18.3975	4.6	17	64856498	62844156	62366656	56901784	16.85	8.92	384.81
S41	female	LET	59.5521	26.60826	4.3	33	46380788	45084548	44741886	40738791	12.03	7.27	369.47
S42	female	LET	59.5521	26.60826	4.3	36	46539888	44942891	44542668	40288199	11.93	7.56	371.35
S43	female	FAL	55.412955	12.931189	9.8	29	44422988	43191463	42867562	38818162	11.62	7.04	387
S44	female	FAL	55.412955	12.931189	9.8	28	44980018	43519423	43174784	39637313	11.95	7.17	386.08
S45	male	SYL	55.016166	8.439550	28.9	39	60809022	59192864	58753924	53143246	15.73	8.56	373.84
S46	male	SYL	55.016166	8.439550	28.9	26	57211502	55688535	55274846	49799828	14.74	8.4	374.03
S47	male	KIE	54.436621	10.17228	18	32	52163506	50721768	50346708	45023344	13.33	8.14	380.26
S48	male	KIE	54.436621	10.17228	18	33	59561780	57906928	57451994	52022840	15.51	8.69	381.48
S49	male	KIE	54.436621	10.17228	18	31	50842586	49392770	49017606	44150548	13.15	7.88	380.16
S5	male	NYN	58.8790698	17.935061	6	24	48136868	46740050	46372224	42197802	12.59	7.32	376.67
S50	male	KIE	54.436621	10.17228	18	32	70840292	68994230	68484716	61556269	18.36	9.95	393.8
S51	female	NYN	58.8790698	17.935061	6	28	57912144	55709732	55285338	49719403	14.83	8.35	387.28
S52	female	NYN	58.8790698	17.935061	6	43	45198976	43755361	43409824	39073539	11.53	7.18	376.31
S53	female	BAR	62.8630833	18.3975	4.6	18	53204092	51613898	51197438	46606570	13.98	7.87	397.72
S54	female	BAR	62.8630833	18.3975	4.6	17	67193056	65208735	64691376	58220165	17.28	9.31	399

S55	female	LET	59.5521	26.60826	4.3	27	45627856	44381119	44043566	40020911	11.82	7.15	371.47
S56	female	LET	59.5521	26.60826	4.3	27	49241638	47879579	47518742	42867415	12.74	7.61	382.12
S57	male	FAL	55.412955	12.931189	9.8	32	53853956	52186204	51764358	46379606	13.81	8.25	396.87
S58	male	FAL	55.412955	12.931189	9.8	31	48604482	47119809	46753202	42028951	12.58	7.77	394.99
S59	male	SYL	55.016166	8.439550	28.9	23	52963100	51536646	51142932	45914061	13.66	8.05	394.22
S6	male	NYN	58.8790698	17.935061	6	23	53610228	52153201	51764414	47154571	13.92	7.82	373.57
S60	male	SYL	55.016166	8.439550	28.9	23	44376314	43229355	42902698	38295557	11.43	7.27	393.46
S61	male	SYL	55.016166	8.439550	28.9	23	50150228	48842901	48471972	43611845	13.01	7.81	395.23
S62	male	SYL	55.016166	8.439550	28.9	24	53219332	51778288	51372236	46356208	13.87	8.07	397.38
S63	male	KIE	54.436621	10.17228	18	31	45157340	43911400	43576258	39284752	11.79	7.4	393.17
S64	male	KIE	54.436621	10.17228	18	30	64161760	62357886	61833264	55332302	16.39	9.25	393.29
S65	female	NYN	58.8790698	17.935061	6	24	57552266	55939246	55491846	50139709	15.04	8.57	393.1
S66	female	NYN	58.8790698	17.935061	6	21	41545924	40452609	40146890	36478915	11	6.79	393.16
S67	female	BAR	62.8630833	18.3975	4.6	18	40847930	39735959	39425668	35714454	10.71	6.78	385
S68	female	BAR	62.8630833	18.3975	4.6	17	52104600	50722288	50345550	45557562	13.67	8.04	385.78
S69	male	LET	59.5521	26.60826	4.3	39	54058430	52458887	52051058	46460762	13.62	8.3	368.57
S7	male	BAR	62.8630833	18.3975	4.6	20	41735988	40611287	40308232	36537433	10.92	6.84	376.44
S70	male	LET	59.5521	26.60826	4.3	32	49486134	48035380	47659980	42943215	12.68	7.72	378.01
S71	male	FAL	55.412955	12.931189	9.8	33	53822154	52317878	51917988	46837175	13.99	8.22	388.15
S72	male	FAL	55.412955	12.931189	9.8	30	59383166	57356093	56921304	50808323	15.14	8.79	383.64
S73	female	SYL	55.016166	8.439550	28.9	26	41531640	40400645	40088336	36348810	10.87	6.81	388.05
S74	female	SYL	55.016166	8.439550	28.9	24	48253694	46856163	46505036	42070531	12.48	7.41	386.77
S75	male	FAL	55.412955	12.931189	9.8	28	48901972	47102565	46734180	42471863	12.77	7.62	398.07
S76	male	FAL	55.412955	12.931189	9.8	30	54727256	52172085	51768210	46714855	13.99	8.21	394.86
S77	male	KIE	54.436621	10.17228	18	27	54971942	53397990	52993618	47682831	14,25	8.39	391.34
S78	male	KIE	54.436621	10.17228	18	29	74182672	72307267	71771586	65049831	19.4	10.26	391.66
S79	female	NYN	58.8790698	17.935061	6	27	51125432	49844267	49475822	44547647	13.29	7.89	384.18
S8	male	BAR	62.8630833	18.3975	4.6	18	41776818	40605236	40286314	36225955	10.81	6.98	378.65
S80	female	NYN	58.8790698	17.935061	6	31	53096178	51557239	51160266	46567931	13.96	8	392.39

S81	male	BAR	62.8630833	18.3975	4.6	20	46138468	44815095	44443792	40083115	11.95	7.4	394.98
S82	male	BAR	62.8630833	18.3975	4.6	18	46487280	45206943	44852368	40245067	12.01	7.49	391.06
S83	male	LET	59.5521	26.60826	4.3	39	56123940	54610807	54200548	48467659	14.28	8.38	382.43
S84	male	LET	59.5521	26.60826	4.3	24	55394104	53829494	53424150	47684275	14.08	8.33	383.97
S85	male	NYN	58.8790698	17.935061	6	28	57048792	55082164	54657306	48888229	14.51	8.43	393.84
S86	male	NYN	58.8790698	17.935061	6	22	58154884	56657681	56204760	51137618	15.29	8.43	401.78
S87	female	SYL	55.016166	8.439550	28.9	26	62919890	61286537	60817766	54723790	16.32	9.02	389.66
S88	female	SYL	55.016166	8.439550	28.9	23	65075134	63336434	62821978	56786117	16.7	8.92	381.06
S89	female	KIE	54.436621	10.17228	18	33	48427088	47150565	46794656	41828226	12.37	7.56	382.58
S9	male	SYL	55.016166	8.439550	28.9	23	54509806	52932957	52517236	47354023	14.03	8.06	378.54
S90	female	KIE	54.436621	10.17228	18	31	37045098	36076704	35816298	32277497	9.59	6.29	379.6
S91	male	FAL	55.412955	12.931189	9.8	30	36778390	35393559	35095258	31680643	9.49	6.22	389.75
S92	male	FAL	55.412955	12.931189	9.8	27	38582222	34925224	34645566	30986369	9.27	6.39	388.5
S93	male	LET	59.5521	26.60826	4.3	24	60554766	58849331	58393636	52673246	15.51	8.83	380.96
S94	male	LET	59.5521	26.60826	4.3	33	51305380	49375369	48965854	43399599	12.4	8.55	349.06
S95	male	BAR	62.8630833	18.3975	4.6	17	70846994	68870460	68353680	61093010	18.09	9.81	389.65
S96	male	BAR	62.8630833	18.3975	4.6	18	44379656	43138160	42810028	38388253	11.46	7.31	386.37

Table S5A. Summary statistics for the RRBS of experimental fish. Samples of the two-generation salinity acclimation experiment with sticklebacks from the Kiel population (20 PSU) are listed with their respective sex (f = female, m = male), parental and offspring salinity treatment condition and standard length. To align quality filtered reads we used *Bismark v0.17.0* (51) with *Bowtie2 v2.3.3*. The conversion rates were calculated using the *cegxQC* software (50) on the unfiltered data (see Methods for details).

ID	sex	parental salinity (PSU)	offspring salinity (PSU)	standard length (mm)	total reads	unique aligned	unaligned	% mapping efficiency	% methylated Cs in CpGs	% methylated Cs in CHGs	% methylated Cs in CHHs	conversion rate of Cs	conversion rate of mCs
RS12	f	6	6	34	19,193,500	13,653,401	3,387,767	71.1	47.6	0.4	0.4	99.7	2.4
RS28	f	6	6	32	21,245,180	14,488,176	4,014,433	68.2	55.4	0.5	0.4	99.6	2.4
RS34	m	6	6	34	21,776,314	14,731,355	3,937,799	67.6	55.8	0.5	0.4	99.7	2.3
RS39	m	6	6	35	15,295,670	10,362,419	2,886,758	67.7	53	0.4	0.4	99.6	2.4
RS47	m	6	6	33	21,588,032	15,209,910	4,024,708	70.5	54.1	0.5	0.4	99.6	2.3
RS55	f	6	6	32	14,812,705	10,392,489	2,728,068	70.2	57.9	0.5	0.4	99.6	2.3
RS6	f	6	6	39	20,614,412	12,789,884	3,554,303	62	56.1	0.5	0.4	99.6	2.5
RS68	f	6	6	35	15,441,713	11,117,645	2,848,520	72	56.7	0.5	0.4	99.6	2.5
RS69	m	6	6	34	22,005,986	15,131,296	4,024,938	68.8	57.6	0.5	0.4	99.6	2.3
RS73	f	6	6	35	19,651,081	13,388,904	3,536,242	68.1	54.8	0.5	0.4	99.6	2.5
RS78	m	6	6	36	19,552,515	13,575,577	3,811,237	69.4	52.7	0.5	0.4	99.5	2.3
RS9	m	6	6	36	18,521,081	11,376,729	3,324,153	61.4	48.6	0.4	0.3	99.6	2.5
RS1	f	20	6	33	19,410,063	12,672,156	3,393,105	65.3	59.8	0.5	0.4	99.6	2.4
RS18	m	20	6	33	22,139,187	14,733,925	4,023,405	66.6	58.3	0,5	0.4	99.5	2.5
RS19	m	20	6	34	25,669,395	17,692,358	4,727,760	68.9	57.8	0.5	0.4	99.5	2.3
RS22	m	20	6	34	21,431,022	14,848,843	3,781,126	69.3	60.6	0.5	0.4	99.7	2.5
RS24	f	20	6	34	18,563,526	12,700,457	3,099,110	68.4	64.9	0.5	0.4	99.5	2.4
RS33	f	20	6	31	19,219,593	12,738,183	3,475,010	66.3	57	0.5	0.4	99.7	2.4
RS5	f	20	6	39	18,474,615	12,876,991	3,113,033	69.7	50.8	0.5	0.4	99.6	2.4
RS52	m	20	6	34	19,884,602	14,145,657	3,763,333	71.1	53.7	0.5	0.4	99.7	2.6
RS61	f	20	6	35	20,691,948	14,272,889	3,761,703	69	55.4	0.5	0.4	99.6	2.2
RS77	m	20	6	34	17,922,261	12,485,632	3,351,014	69.7	50.8	0.5	0.4	99.6	2.5

RS79	m	20	6	33	11,936,993	7,973,063	2,435,528	66.8	52.1	0.5	0.4	99.6	2.4
RS11	f	20	20	31	20,243,439	14,268,055	3,663,464	70.5	46.8	0.4	0.4	99.6	2.4
RS14	m	20	20	33	18,299,908	12,488,117	3,243,269	68.2	57.8	0.5	0.4	99.6	2.1
RS2	f	20	20	31	20,137,550	13,212,235	3,509,171	65.6	61.3	0.5	0.4	99.5	2.4
RS27	m	20	20	34	17,234,518	11,807,735	3,147,777	68.5	47.9	0.4	0.4	99.6	2.4
RS30	f	20	20	33	20,652,539	14,071,954	3,717,746	68.1	56.5	0.5	0.4	99.6	2.4
RS36	f	20	20	33	18,842,860	13,035,496	3,439,400	69.2	59.2	0.5	0.4	99.6	2.5
RS46	f	20	20	31	14,955,973	10,166,362	2,653,749	68	51.4	0.5	0.4	99.4	2.4
RS48	m	20	20	36	23,172,203	16,234,065	4,254,154	70.1	53.6	0.4	0.4	99.6	2.5
RS59	f	20	20	29	19,697,111	14,065,491	3,437,252	71.4	57.5	0.5	0.4	99.7	2.4
RS62	m	20	20	34	21,508,796	14,858,533	4,028,818	69.1	52.6	0.5	0.4	99.5	2.4
RS7	m	20	20	34	15,890,812	10,638,953	3,121,759	67	55.1	0.5	0.4	99.6	2.4
RS75	m	20	20	33	22,640,803	14,627,178	4,154,518	64.6	50.6	0.5	0.4	99.6	2.3
RS16	m	20	33	36	23,379,805	16,349,654	4,392,411	69.9	50.3	0.4	0.3	99.7	2.5
RS17	f	20	33	33	15,365,773	10,794,519	2,861,846	70.3	53.4	0.4	0.3	99.6	2.5
RS23	m	20	33	38	23,257,469	15,971,106	4,134,190	68.7	63.9	0.5	0.4	99.4	2.3
RS31	f	20	33	34	18,197,686	12,270,798	3,370,685	67.4	55.4	0.5	0.4	99.6	2.4
RS45	m	20	33	34	19,293,724	13,575,990	3,709,491	70.4	46.6	0.4	0.4	99.6	2.4
RS49	f	20	33	35	22,586,319	16,247,681	3,991,975	71.9	51.5	0.4	0.4	99.6	2.5
RS56	m	20	33	35	12,837,376	8,486,651	2,417,279	66.1	56.7	0.5	0.4	99.7	2.7
RS65	f	20	33	37	14,645,372	10,073,076	2,768,136	68.8	56	0.4	0.3	99.6	2.5
RS66	m	20	33	32	20,732,423	14,118,267	3,957,707	68.1	60	0.5	0.4	99.6	2.4
RS72	f	20	33	30	23,099,522	15,824,314	4,015,465	68.5	55.8	0.5	0.4	99.6	2.4
RS8	m	20	33	30	14,834,881	9,428,882	2,726,520	63.6	59.9	0.5	0.4	99.5	2.4
RS10	f	33	33	29	17,199,844	11,861,027	3,060,336	69	49.1	0.4	0.4	99.5	2.6
RS13	m	33	33	32	19,322,020	12,524,534	3,508,974	64.8	49	0.4	0.4	99.6	2.5
RS26	m	33	33	35	18,323,492	12,085,071	3,485,139	66	53.2	0.5	0.4	99.7	2.4
RS29	f	33	33	32	20,178,134	13,061,973	3,634,233	64.7	50.1	0.5	0.4	99.6	2.4
RS38	f	33	33	33	19,425,374	12,864,087	3,481,184	66.2	55.1	0.5	0.4	99.6	2.4
RS42	m	33	33	35	16,966,704	11,139,312	3,146,420	65.7	54.9	0.5	0.4	99.7	2.6
RS44	m	33	33	37	15,454,040	10,661,431	2,861,799	69	47.3	0.4	0.3	99.6	2.5
RS50	f	33	33	32	22,655,597	15,608,421	3,964,156	68.9	60.2	0.5	0.4	99.7	2.4
RS58	f	33	33	37	19,602,242	13,608,515	3,279,118	69.4	60.2	0.5	0.4	99.6	2.3
RS60	m	33	33	36	19,446,537	13,374,949	3,584,079	68.8	56.8	0.5	0.4	99.6	2.4
RS63	f	33	33	35	18,322,127	12,880,934	3,404,665	70.3	52.9	0.5	0.4	99.6	2.5
RS71	m	33	33	37	16,379,683	11,242,454	3,138,323	68.6	58.1	0.5	0.4	99.6	2.4

Table S5B. Summary statistics for the RRBS of wild-caught fish. Samples of three populations (KIE = Kiel, NYN = Nynäshamn, SYL = Sylt) collected in- and outside of the Baltic Sea salinity gradient are listed with their respective sex (f = female, m = male) and salinity of origin. To align quality filtered reads we used *Bismark v0.17.0* (51) with *Bowtie2 v2.3.3*. The conversion rates were calculated using the *cegxQC* software (50) on the unfiltered data (see Methods for details).

ID	sex	location	salinity	total reads	unique aligned	unaligned	% mapping effiency	% methylated Cs in CpG	% methylated Cs in CHG	% methylated Cs in CHH	Conversion rate of Cs	Conversion rate of mCs
S3	f	KIE	18	8,753,702	4,874,582	2,438,154	55.7	57.3	0.7	0.6	99.1	2.56
S4	f	KIE	18	8,571,146	5,056,022	2,321,568	59.0	63.4	0.8	0.7	99.2	2.49
S5	m	NYN	6	7,232,265	4,454,780	1,997,286	61.6	62.6	0.7	0.7	99.3	2.50
S6	m	NYN	6	8,181,388	4,890,678	2,307,322	59.8	59.1	0.7	0.7	99.2	2.57
S9	m	SYL	28.9	7,791,070	4,420,213	2,295,644	56.7	58.1	0.7	0.6	99.2	2.50
S10	m	SYL	28.9	7,753,903	4,145,109	2,254,765	53.5	57.6	0.7	0.7	99.3	2.57
S17	f	SYL	28.9	11,091,390	7,159,358	2,352,179	64.5	56.4	0.6	0.5	99.5	2.48
S18	f	SYL	28.9	11,411,538	7,481,972	2,501,782	65.6	59.3	0.6	0.5	99.4	2.39
S19	f	KIE	18	5,744,795	3,662,505	1,369,060	63.8	58.4	0.6	0.5	99.4	2.49
S20	f	KIE	18	10,006,121	6,408,060	2,200,372	64.0	58.3	0.6	0.5	99.4	2.43
S21	m	NYN	6	10,724,717	6,639,862	2,498,410	61.9	58.5	0.6	0.5	99.4	2.39
S22	m	NYN	6	9,966,267	6,734,287	2,092,066	67.6	64.5	0.6	0.5	99.3	2.30
S32	f	SYL	28.9	8,570,141	4,986,526	2,135,955	58.2	63.1	0.8	0.7	99.2	2.47
S33	f	KIE	18	9,332,513	5,401,129	2,435,485	57.9	57.8	0.7	0.6	99.3	2.52
S34	f	KIE	18	7,902,135	4,901,313	2,126,350	62.0	57.3	0.7	0.6	99.3	2.44
S35	m	NYN	6	9,525,328	5,327,975	2,421,326	55.9	58.9	0.7	0.6	99.3	2.39

S36	m	NYN	6	14,623,815	9,020,345	3,974,462	61.7	57.2	0.7	0.6	99.4	2.43
S37	f	NYN	6	11,349,272	6,980,832	2,928,035	61.5	57.8	0.6	0.6	99.4	2.40
S38	f	NYN	6	8,248,343	4,698,496	2,183,045	57.0	60.4	0.7	0.6	99.4	2.41
S45	m	SYL	28.9	11,310,329	7,263,715	2,830,149	64.2	55.6	0.6	0.5	99.4	2.48
S46	m	SYL	28.9	8,604,268	5,288,360	2,123,524	61.5	57.5	0.6	0.5	99.4	2.41
S47	m	KIE	18	9,263,216	5,258,462	2,438,100	56.8	60.6	0.6	0.6	99.4	2.41
S48	m	KIE	18	11,163,270	7,214,590	2,669,147	64.6	56.1	0.6	0.5	99.5	2.43
S49	m	KIE	18	8,255,526	4,765,556	2,502,234	57.7	60.5	0.8	0.7	99.2	2.45
S50	m	KIE	18	7,028,398	3,784,613	2,259,871	53.8	62.5	0.8	0.7	99.3	2.56
S51	f	NYN	6	10,333,052	5,838,362	2,893,012	56.5	57.4	0.7	0.6	99.4	2.48
S52	f	NYN	6	8,266,988	4,418,322	2,705,975	53.4	60.9	0.8	0.7	99.3	2.51
S59	m	SYL	28.9	8,506,803	4,689,790	2,438,004	55.1	58.1	0.8	0.7	99.3	2.45
S60	m	SYL	28.9	7,561,005	4,028,161	2,350,333	53.3	57.6	0.7	0.7	99.3	2.49
S61	m	SYL	28.9	11,144,581	6,658,021	2,667,871	59.7	57.2	0.6	0.5	99.5	2.44
S62	m	SYL	28.9	10,998,006	6,742,826	2,506,560	61.3	57.4	0.6	0.5	99.4	2.44
S63	m	KIE	18	11,152,063	6,896,108	2,623,103	61.8	56.2	0.6	0.5	99.5	2.50
S64	m	KIE	18	10,905,488	6,409,362	2,634,685	58.8	56.2	0.6	0.5	99.5	2.46
S65	f	NYN	6	12,485,843	7,555,803	2,926,648	60.5	53.8	0.6	0.5	99.5	2.49
S66	f	NYN	6	13,455,950	8,490,473	3,088,221	63.1	59.1	0.6	0.5	99.5	2.39
S73	f	SYL	28.9	8,901,147	5,117,469	2,560,420	57.5	56.6	0.8	0.7	99.4	2.50
S74	f	SYL	28.9	6,650,655	3,652,399	2,007,162	54.9	59.5	0.8	0.7	99.5	2.32
S77	m	KIE	18	9,014,946	5,232,127	2,587,729	58.0	58.7	0.8	0.7	99.3	2.37
S78	m	KIE	18	9,650,434	5,788,601	2,744,058	60.0	58.6	0.8	0.7	99.3	2.53
S80	f	NYN	6	8,622,807	5,206,629	2,510,145	60.4	57.0	0.8	0.7	99.4	2.50
S85	m	NYN	6	10,321,779	5,874,747	2,665,411	56.9	57.7	0.6	0.5	99.4	2.46
S86	m	NYN	6	8,879,732	5,427,464	2,286,757	61.1	58.5	0.6	0.5	99.5	2.45
S87	f	SYL	28.9	10,835,874	6,552,344	2,484,095	60.5	55.5	0.6	0.5	99.5	2.51

S88	f	SYL	28.9	11,103,294	6,802,303	2,640,248	61.3	58.5	0.6	0.5	99.4	2.50
S89	f	KIE	18	13,340,852	7,663,569	2,949,110	57.4	57.2	0.6	0.5	99.4	2.40
S90	f	KIE	18	12,725,951	7,916,399	2,971,993	62.2	55.7	0.6	0.5	99.4	2.45

Table S6. The number of DMS for each of the two pairwise population comparisons (pop-DMS). The overlap with the DMS obtained from the field (KIE) versus experiment (control group from KIE population) comparison, which were filtered out to remove potential laboratory artifacts. The resulting number of pop-DMS, and their associated number of genes.

Comparison	# of pop-DMS	overlap with field (KIE) vs. experiment (control group) comparison	# of pop-DMS used in downstream analysis	# of pop-DMS associated to genes	# of genes associated to pop-DMS
KIE vs. NYN (20 vs. 6 PSU)	1,990	520	1,470	1,098	655
KIE vs. SYL (20 vs. 33 PSU)	1,663	505	1,158	871	510
